# An Efficient Method for the Synthesis and In Silico Study of Novel Oxy-Camalexins

**DOI:** 10.3390/molecules30092049

**Published:** 2025-05-04

**Authors:** Maria Bachvarova, Yordan Stremski, Donyo Ganchev, Stela Statkova-Abeghe, Plamen Angelov, Iliyan Ivanov

**Affiliations:** 1 Department of Organic Chemistry, University of Plovdiv “Paisii Hilendarski”, 24 Tsar Asen Str., 4000 Plovdiv, Bulgaria; bychvarova@uni-plovdiv.bg (M.B.); stab@uni-plovdiv.bg (S.S.-A.); angelov@uni-plovdiv.bg (P.A.); iiiliyan@abv.bg (I.I.); 2 Department of General Chemistry, Agricultural University of Plovdiv, 12 Mendeleev Blvd, 4000 Plovdiv, Bulgaria; d_ganchev@au-plovdiv.bg

**Keywords:** camalexin, methoxycamalexins, thiazole, indole, multicomponent reactions, *N*-acyliminium reagents, α-amidoalkylation, in silico study, RDKit, MolecularGraph

## Abstract

Methoxycamalexins are close structural derivatives of the indolic phytoalexin *Camalexin*, which is a well-known drug lead with an antiproliferative and antioxidant profile. 6-methoxycamalexin, 7-methoxycamalexin, and 6,7-dimethoxycamalexin are natural bioactive products, and there is significant interest in the development of efficient methods for the synthesis of structurally related analogues. Herein, we describe an efficient and high-yielding method for the synthesis of variously substituted hydroxy-, bezyloxy, and methoxycamalexins. A set of methoxy-, hydroxy-, and benzyloxy-indoles were successfully amidoalkylated with *N*-acyliminium reagents derived in situ from the reaction of thiazole or methylthiazoles with Troc chloride. Eleven novel *N*-acylated analogues were synthesized, with yields ranging from 77% to 98%. Subsequent oxidative reactions with *o*-chloranil or DDQ led to 10 novel oxy-camalexins in 62–98% yield. This two-step approach allowed the synthesis of two 4,6-dimethoxy camalexins, which are difficult to obtain using published methods. The structure of the obtained products was unequivocally determined by ^1^H-, ^13^C{^1^H}-, HSQC-NMR, FTIR, and HRMS spectral analyses. An in silico assay was carried out on the obtained products to assess their general toxicity and physicochemical properties, including their compliance with Lipinski’s rule of five. The results indicate that all compounds have good potential to be developed as drugs or agrochemicals.

## 1. Introduction

Plants produce a variety of antimicrobial secondary metabolites as part of their defence strategy against pathogens [1,2]. Examples include resveratrol, a stilbenoid synthesized by grapevine (*Vitis vinifera*) during fungal infections [1,3], and sakuranetin, a flavonoid induced in rice (*Oryza sativa*) under various stress conditions [2,4]. These compounds highlight the importance of plant metabolites as the first line of defence [5].

Phytoalexins have diverse structures and play a key role in the defence mechanisms of plants against various pathogens and pests [6,7,8,9,10]. The protective mechanism of action of phytoalexins, including camalexins, involves the inhibition of bacterial and fungal growth by disrupting cell membranes [11], induction of apoptosis in fungal cells, and impact on signalling pathways responsible for pathogen resistance [7,12]. In inhibiting key enzymes (catalase and oxiperoxidase), hydrogen peroxide is released, leading to an oxidative burst, which is a part of Systemic Acquired Resistance (SAR) [7,12,13]. This can be artificially stimulated using chemical substances (natural or synthetic) or physical agents such as electricity, ultrasound, and ultraviolet light. Such mechanisms allow the protection of plants from adverse abiotic factors like extreme temperatures and drought, as well as from pests (insects, mites, pathogens, viruses, and others). This approach in plant protection is called the Induction of Systemic Resistance (ISR) [14,15,16]. The combination of ISR and SAR can enhance protection against pathogens that are resistant to both pathways [17]. This is the main mechanism by which plants defend themselves, and also a strategy that can be applied in the development of new substances with fungicidal action.

The indole moiety finds wide application in medicinal chemistry [18], particularly in the design of antitumor agents [19]. A number of indole derivatives have been approved for clinical use [19]. Indole alkaloids belong to one of the largest classes of compounds found in various natural sources [20]. Many plant species generate indole alkaloids, known as phytoalexins [9,21]. They were first described by Müller and Börger [22] and represent specific chemical compounds that plants synthesize in response to infection or stress factors [9,23]. They are characterized by significant structural diversity [6] and exhibit a wide range of biological properties—antimicrobial [7,24,25], antifungal [26,27,28,29], antiviral [29], inhibitory [30,31,32,33], antioxidant [34], anti-inflammatory [35,36], antitumor [36,37,38,39,40,41], antiproliferative [41,42,43], and cytotoxic [44,45], along with other biological functions [8,23,37].

Camalexin is known to exhibit some toxicity towards plant cells, with a concentration of 100 μg/mL being sufficient to induce cell death in suspension cultures. However, it is not the primary cause of plant cell death during pathogenic infection [46].

**Figure 1:**
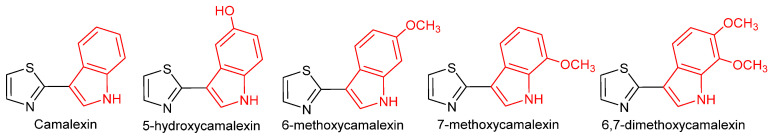
Natural indole phytoalexins.

The literature data indicate that some 5-substituted camalexins possess higher biological activity than camalexin itself [26,31,47], and their fungicidal effectiveness is highly dependent on the structural modifications of camalexin [29]. Figure 1 visualizes natural indole phytoalexins, structural analogues of camalexin (2-(1*H*-indol-3-yl)thiazole). Various synthetic approaches have been developed for its synthesis [44,47,48,49,50,51].

These compounds have been isolated from plant sources [26,28,30], listed in Table 1.

The biological activity of these compounds is highly dependent on the position and type of substituents in their molecular structure [26,28,30]. Natural camalexins and their structural analogues exhibit remarkable fungicidal activity, making them valuable for the development of new antifungal agents [9,12].

Several methods for the synthesis of methoxycamalexins have been published, with the first reported by Ayer et al. in 1992, including the preparation of 5-methoxycamalexin and 6-methoxycamalexin. One of the reactions proceeds for 43 h with 50% recovery of the starting 5-methoxyindole [47]. Pedras et al. also synthesized various methoxy- and dimethoxycamalexins, concluding that the presence of two methoxy groups leads to reaction in the activated benzene ring. Thus, 4,6-dimethoxycamalexin (yield 50%) and 5,7-dimethoxycamalexin (yield 37%) were obtained. The remaining target dimethoxy derivatives were synthesized using the Hantzsch reaction from the corresponding dimethoxyindole-3-thiocarboxamides. Despite its effectiveness, the Hantzsch reaction is not applicable for the synthesis of 4,6-dimethoxycamalexin and 5,7-dimethoxycamalexin [50].

Another method for the synthesis of methoxycamalexins involves the application of the Suzuki reaction and requires an expensive catalyst—palladium-tetrakis (triphenylphosphine) [Pd (PPh_3_)_4_]. This method was developed by Tasch et al. and its utility was demonstrated with the synthesis of 5-methoxycamalexin and 6-methoxycamalexin [49]. In the last decade, other alternative methods for the synthesis of variously substituted camalexins have been proposed [29,51,52,53].

In our previous studies, we successfully synthesized camalexin, benzocamalexin, and aza-camalexin using a combination of α-amidoalkylation and oxidation for heterocyclic ring coupling. This method is versatile and has proven its reliability on a gram-scale preparation of camalexin [51,52]. In continuation of this work, the current study is aimed at expanding the scope of our method to variously oxygenated indole substrates in order to provide access to novel oxy-camalexins.

**Figure 2:**
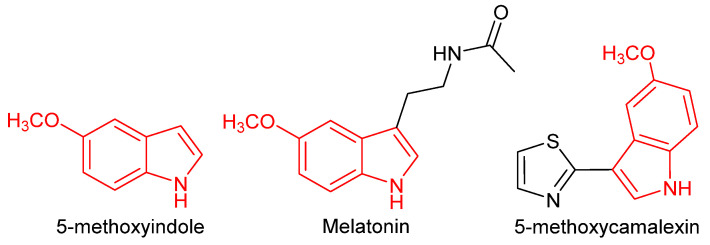
Antifungal agents [29,50,54].

*Fusarium graminearum* is a destructive fungal pathogen that threatens the production and quality of wheat. Controlling it is of great importance to producers [54]. Therefore, it is important to develop alternative methods and eco-friendly fungicidal agents with low toxicity to plants that can provide effective control of *Fusarium*. Kong et al. presented an extensive report on 5-methoxyindole, which is a cost-effective simplified melatonin analogue (Figure 2).

This study shows that 5-methoxy-1*H*-indole (Figure 2) possesses higher antifungal activity against *Fusarium graminearum* compared to melatonin. In addition, its mechanism of action was also studied, and it was found that 5-methoxy-1*H*-indole inhibits the growth of *F. graminearum* at concentrations ≥ 2 mM. Through the release of H_2_O_2_, the normal morphology of *Fusarium graminearum* is disrupted, and this leads to structural anomalies in the hyphae and spores, resulting in cell death [54]. This study clearly reveals and emphasizes the mechanism of the antifungal action of 5-methoxy-1*H*-indole.

Building on the high bioactivity of 5-substituted indole derivatives [29], the established fungicidal action of 5-methoxyindole [54] and 5-methoxycamalexin [26,29,50], a successful synthesis of novel oxy-camalexins would provide access to hybrid molecules with potential agrochemical application and promising candidates for a new generation of plant protection agents.

## 2. Results and Discussion

### 2.1. Synthesis of Novel Analogues of Oxy-Camalexins by Multicomponent Amidoalkylation of Oxy-Indoles

The amidoalkylation reaction is a synthetic tool used to generate new carbon–carbon (C–C) bonds and to functionalize various heterocycles. This approach finds wide application for synthetic modifications of heterocyclic compounds, including five- and six-membered systems such as thiophenes, pyrroles, furans, and indoles [55]. The main classes of amidoalkylating reagents, the synthetic pathways by which they are obtained, and their reactivity towards various nucleophiles were thoroughly reviewed and described by Mazurkiewicz et al. [56].

In our previous studies, it was found that *N*-acyl benzothiazolium reagents are less reactive than *N*-acyl thiazolium reagents in α-amidoalkylation reactions with indoles. The rapid release of hydrochloric acid leads to the decomposition of the target products. Control over the reaction to ensure optimal conditions is achieved through the gradual addition of triethylamine (Et_3_N). Conducting the reactions in the absence of Et_3_N leads to the formation of triindolylmethane as the main product according to our previous investigations [51].

**Scheme 1:**
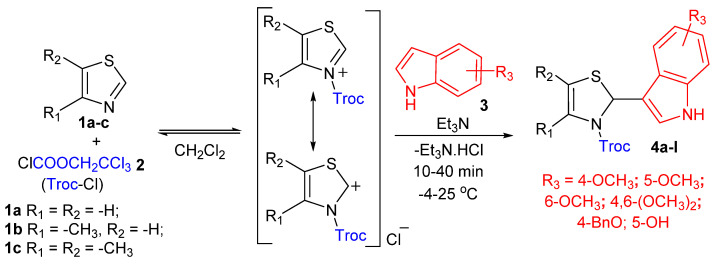
*α*−amidoalkylation of various oxy-indoles.

The present study expands the scope of the α-amidoalkylation reaction using variously substituted oxy-indoles—4-, 5-, and 6-methoxyindoles, 4,6-dimethoxyindole, 5-hydroxyindole, and 4-benzyloxyindole (Scheme 1).

In the examined reactions, *N*-acyl-thiazolium reagents were successfully applied, derived in situ by mixing thiazole/4-methylthiazole/4,5-dimethylthiazole (**1a–c**) with 2,2,2-trichloroethyl chloroformate (Troc-Cl, **2**). 2,2,2-Trichloroethyl chloroformate was preferred due to the faster reaction rate and higher yields of the target products, compared to ethyl chloroformate.

The substituents in the thiazole ring influenced the reactivity of *N*-acyliminium reagents. Methylated thiazoles showed slightly higher reaction rates, probably because of the electron-donating properties of the methyl groups, which increase the nucleophilicity of the nitrogen and stabilize the *N*-acyliminium ions, thus facilitating their formation. The experimental results presented in (Table 2) show the difference in the reaction rate, depending on the thiazole used (**1a–c**). Reactions with 4,5-dimethylthiazole proceeded generally faster, within 10 to 30 min. The position of the methoxy group in the indole substrate did not significantly affect the reaction time and the yield of the final products. The presence of a second methoxy substituent in 4,6-dimethoxyindole, however, led to faster reaction, with the reaction time not depending on the type of thiazole. In the amidoalkylation of 5-hydroxyindole, a second product was observed, leading to a decrease in the yield of **4l** (77%). It is likely that a competing amidoalkylation reaction also occurs in the benzene ring of indole because of the strong electron-donating properties of the OH group.

To determine the working scope and applicability of this two-step method, it was necessary to test its feasibility for synthesis in larger amounts. For this purpose, we chose a target product—2,2,2-trichloroethyl 5-methoxy-3- (thiazol-2-yl)-1*H*-indole-1-carboxylate (**4e**), a synthetic analogue of 5-methoxycamalexin, which has proven fungicidal activity. The reaction in this case was easily scaled up to 3 mmol and gave the product **4e** in 96% yield (1.255 g). On this scale, the product was isolated by recrystallization, instead of chromatography, thus confirming the efficiency of the method on a gram scale. The reaction was followed by oxidative aromatization with an equimolar amount of *o*-chloranil in acetonitrile, providing 5-methoxycamalexin (**5e**) in 90% yield (0.6975 g).

The structures of the obtained products were determined on the basis of their ^1^H-, ^13^C{^1^H}-, HSQC-NMR, FTIR, and HRMS spectra. The ^1^H-NMR spectra of the analyzed compounds indicate singlets in the range of *δ* = 6.80–7.35 ppm for the proton on the *sp*^3^ C-2 atom in the thiazole ring. The ^1^H-, ^13^C{^1^H}-HSQC measurements confirmed the exact location of these characteristic signals (see the Supplementary Materials section, HSQC-NMR spectrum of **4c**, Figure S11). Recording the proton NMR spectra of the *N*-acylated products at room temperature in DMSO-d_6_ resulted in the registration of highly broadened signals, making their correct integration and subsequent interpretation impossible (see the Supplementary Materials section, ^1^H-NMR spectrum of **4i**, Figure S34). In the carbon spectra recorded under these conditions, the absence of important characteristic signals was also observed. To average out the rotamers observed in *N*-acylated oxy-camalexin analogues and achieve adequate peak assignment and structure determination, the NMR spectra were measured at 80 °C in DMSO-d_6_. Under these conditions, averaged signals were successfully observed, fully corresponding to the expected ones, spectrally confirming the analyzed structures of the newly synthesized products. Original spectra are available in the Supplementary Materials section (Figures S1–S96).

### 2.2. Synthesis of Oxy-Camalexins

**Scheme 2:**
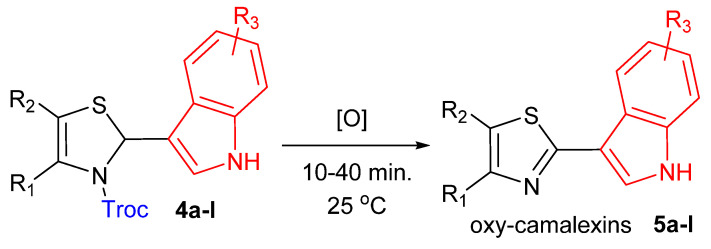
Oxidative rearomatization reactions for obtaining oxy-camalexins **5a–l**.

In the next stage, the obtained *N*-acylated compounds **4a–l** were successfully oxidized with an equivalent amount of *o*-chloranil (2,3,5,6-tetrachloro-1,2-benzoquinone) or DDQ (2,3-dichloro-5,6-dicyano-benzoquinone). The reaction conditions for oxidative rearomatization were successfully optimized (Scheme 2).

The optimization of reaction conditions for the synthesis of oxy-camalexins includes varying the type and amount of solvent, as well as using different oxidizing reagents. The oxidative reaction with *o*-chloranil (3,4,5,6-tetrachloro-1,2-benzoquinone) proceeded in acetonitrile at room temperature for 10 to 40 min. Ten novel oxy-camalexins were obtained with high yields ranging from 62% to 98%, presented in a general scheme (Scheme 2).

The oxidative rearomatization was also tested using the oxidant DDQ (2,3-dichloro-5,6-dicyano-1,4-benzoquinone). The synthetic transformations of *N*-acyl methoxy-indoles (**4b** and **4i**) were carried out at room temperature in dichloromethane, using an equimolar amount of the oxidizing agent (see Supplementary Materials, proposed mechanism of oxidative reaction, Scheme S1). The obtained products (**5b** and **5i**) were isolated with approximately 10% higher yields compared to the reactions performed with *o*-chloranil (Table 3). Despite the higher yields and reduced reaction time, the oxidizing agent *o*-chloranil was preferred due to environmental considerations and the possibility of more efficient chromatographic separation and isolation of the obtained oxy-camalexins.

## 3. In Silico QSAR Analysis

### In Silico Predictions of Oxy-Camalexins Toxicity, LogP, and Various Other Parameters

In silico toxicology is dynamically developing as a field focused on assessing the safety of chemical substances and predicting their toxicity [57]. The use of QSAR models to evaluate the parameters of synthetic compounds significantly reduces experimental tests with living organisms [58]. In silico analysis of synthetic compounds is essential for biopharmaceutical properties and ecotoxicological studies [59,60]. By applying the in silico machine learning method based on the nearest neighbour, implemented in the software system Toxicity Estimation Software Tool (T.E.S.T.)—version 5.1.2. [61], the following properties of the compounds presented in Table 4 were successfully predicted: oral rat LD_50_, *Tetrahymena pyriformis* IGC_50_, *Daphnia magna* LC_50_, and water solubility at 25 °C.

The obtained predictive results for oral rat LD_50_ values in rats range from 524.03 mg/kg to 3999.40 mg/kg. Compounds **p2**, **p3**, **p5**, **p6**, **p8**, **p9**, **p10**, **p11**, **p12**, **p13**, and **p18** have predictive results for oral rat LD_50_ > 2000 mg/kg, classifying them as compounds with low toxicity (Table 4).

*Daphnia magna* is widely used in ecotoxicological studies. The acute toxicity test with this species is standardized by the U.S. Environmental Protection Agency (EPA), REACH (Registration, Evaluation, Authorization, and Restriction of Chemicals), and OECD Test Guidelines [62,63,64,65]. All compounds have acute toxicity toward *Daphnia magna* between 0.78 and 15.16 mg/L, which means moderate toxicity towards this test organism (Table 4).

Compounds **p1**, **p8**, **p10**, **p14**, **p16**, and **p17** were not synthesized in this work (see Supplementary Materials, Table S1).

In previous in silico studies, Mounika et al. found that camalexin exhibits a good balance between lipophilicity (LogP—3.17), low polar surface area (PSA—20.28 Å^2^), and molar refractivity (MR—59.4), suggesting its favourable properties for crossing cell membranes and interacting with biomolecules. Additionally, all analyzed derivatives maintain high gastrointestinal absorption (GI Absorption) and meet key pharmacokinetic criteria [60]. The above establishes camalexin as a stable structural basis for the synthesis of new analogues with improved biopharmacological characteristics. Building on the established pharmacokinetic and physicochemical profile of this phytoalexin, the current study aims to enhance these parameters through the synthesis and in silico QSAR evaluation of novel analogues. As our main focus is on the potential applications of newly synthesized oxy-camalexins in agriculture. In this regard, we used various predictive models to forecast key physicochemical properties, including their compliance with Lipinski’s rule of five. Lipinski’s rule of five has the following requirements for drug candidate molecules: molecular weight (MW) < 500 Da, number of hydrogen bond acceptors < 10, number of hydrogen bond donors < 5, and octanol/water partition coefficient (LogP) < 5 [66,67,68]. All tested molecules have molecular weight below 500 g/mol (Table 4). The octanol/water coefficient logP (log ow) was calculated using several computational tools: Python programming language and modules RDkit and PadelPy [69,70], EPI Suite [71,72], and VEGA [73] (Table 5).

MolecularGraph.jl [74] was used to check if the tested molecules fall into the rule of five (Lipinski’s rule of five). Most of them have HBD = 1, some of them (**p14** and **p15**) = 0, and **p16**, **p17**, and **p18** = 2, which also fell under the rule of five. All molecules have HBA = 3, except **p10**, **p11**, and **p12** = 4, which are also in accordance with the rule. The calculated logP for all molecules was under 5 (in accordance with the rule) except for **p15**, where all in silico tools established logP between 5.1 and 5.45. However, the logP calculated with the Python language PadelPy module was 4.13. The number of rotatable bonds was between 1 and 4. The results are presented in Table S2, Supplementary Materials.

MolecularGraph.jl [74] is a library in the Julia programming language, specialized for cheminformatics and QSAR analysis. Julia language MolecularGraph.jl was used to calculate atom hybridization in the tested molecules and the number of unpaired and π-electrons (Table S3, Supplementary Materials). There was no *sp* hybridization in the molecules; the *sp*^2^ hybridization was mostly 12, except **p13**, **p14**, and **p15** = 18. The same results were observed for the number of π-electrons. However, *sp*^3^ hybridization was diverse, ranging between 3 and 8. The number of unpaired electrons was 16-17-18 except for **p12**, which had 20, **p13** with 22, **p14** with 23, and **p15** with the highest number = 24 (Table S3).

In the present work, EPI Suite [71] was used to predict the ecotoxicological, physicochemical properties, and DT_50_ (half-life in days) [75]. The persistence of oxy-camalexins in soil, water, and air was assessed (Table 6). EPI Suite shows that all molecules are non-persistent in the air, moderately persistent in the water (except **p16**, which was found to be non-persistent), moderately persistent in the soil (except **p15**, which was found to be persistent in this medium), and persistent in the sediments (except **p15**, which was found to be very persistent).

With the Rdkit module [76], we calculated various molecular descriptors of the tested molecules (Table 7). Regarding the synthetic accessibility score (SA Score) [77] for all molecules, this index was approximately 3, indicating good synthetic accessibility. **p13** has the lowest value of 2.749. According to the values of the Natural Product-likeness Score (NP_Score) [78], all molecules have negative values. The highest value (−0.24) was for **p16**, and the lowest value was for **p5** = −0.89. However, all of the values were in the range of this index for synthetic compounds. The LabuteASA [79] descriptor represents the solvent-accessible surface area (SASA) for molecules. The values ranged between 90.72 (**p16**) and 145.23 (**p15**). With RDkit, we calculated three indexes, BertzCT, AvgIpc, and BalabanJ, showing the complexity of the molecules. [80,81,82]. The BertzCT index values were approximately 580–600, but **p13**, **p14**, and **p15** had values of 789, 822, and 858, indicating more complex structures. The same results were achieved with the AvgIpc index. However, regarding the BalabanJ, these molecules had values 1.63–1.6,5 while the rest of compoundshad values 2.03–2.16 (Table 7).

The TPSA (topological polar surface area) [83] of the most of tested molecules was between 34.48 and 36.22, except for **p10**, **p11**, and **p12** (45.45) and **p16**, **p16**, and **p18** (47.22 Å^2^), which actually determine the excellent ability of molecules to permeate cells. Molar refractivity (Mol MR) [84] varied between 63.98 and 98.8 and was in the range of other drugs (between 40 and 130). The established Quantitative Estimate of Drug-Likeness (QED) [85] was lowest for **p15** and **p14**—0.65 and 0.68—and very high (0.96) for **p11** and **p12**. The rest of the molecules had values of approximately 0.8 (Table 8). The SPS score (Special Score Descriptor), which evaluates the topological complexity of molecules, showed that the tested structures were almost identical in this aspect, except **p14** and **p15**, which had higher score and, respectively, more complexity (Table 7).

The computational models show that all compounds have good potential to be developed as drugs and pesticides according to their QED indexes with excellent permeability into cells towards TPSA values. The QSAR analysis shows that with a few exceptions, the molecules are likely to how no or low persistence in the environment, except, however, sediments. All substances comply with Lipinski’s rule of five. The fraction Csp^3^ descriptor calculates the fraction of C atoms that are sp^3^ hybridized, and the results show a lot more variations towards the values of other molecular descriptors. Compounds **p1**, **p4**, and **p7** have the same values of molecular descriptors listed in Table 8.

Although in silico tools and predictive models, such as RDKit, VEGA, and T.E.S.T., are essential for early screening, they may not fully reflect the in vivo behaviour of the molecules. In this regard, despite the predictive in silico results for ecotoxicity and important physicochemical properties, additional experimental studies are necessary for an accurate assessment of their safety and effective application as agrochemicals.

## 4. Materials and Methods

### 4.1. Chemistry

#### 4.1.1. General Information

The starting materials, *N*-heterocycles (4-methylthiazole, 4,5-dimethylthiazole, thiazole), all differently substituted methoxy-, hydroxy-, and benzyloxyindoles, alkyl chloroformate (2,2,2-trichloro ethyl chloroformate), and triethylamine, and all solvents were obtained from commercial suppliers (Merck) and used without further purification. The melting point of the synthesized compounds was determined on a Kruss M5000 melting point metre (A.Krüss Optronic GmbH, Hamburg, Germany). IR spectra were measured on an ALPHA II FT-IR spectrometer (Bruker Optics GmbH, Ettlingen, Germany) in KBr tablets or attenuated total reflection (ATR) accessories, with absorption frequencies given in reverse centimetres (cm^−1^). High-resolution mass spectral measurements were performed on a Bruker mass spectrometer. ^1^H-, ^13^C{^1^H}-NMR spectra were measured on Bruker Avance AV600 and Bruker Avance NEO AV400 spectrometers (Bruker, Billerica, MA, USA). Deuterated dimethyl sulfoxide (DMSO-d_6_) or chloroform (CDCl_3_) was used as solvent. Chemical shifts (*δ*, ppm) were downfield from tetramethylsilane (TMS) as an internal standard, and *J*-constants were given in Hz. For convenient reaction monitoring—thin-layer chromatography (TLC) plates—Merck Silica gel 60, 0.2 mm, and Al were applied. The aluminum oxide 90 active—neutral (0.063–0.200 mm) (Al_2_O_3_) or silica gel 60 (0.063–0.200 mm, 70–230 mesh ASTM)—were used for column chromatographic separation and purification.

#### 4.1.2. General Procedure for the Synthesis of N-Acylated Oxy-Camalexin Analogues

2,2,2-trichloroethyl chloroformate (1.2 equiv.) was slowly added with magnetic stirring to a solution of the corresponding thiazole/4-methylthiazole/4,5-dimethylthiazole (1 mmol) in dry dichloromethane (CH_2_Cl_2_, 8 mL) at the temperature indicated in Table 2. Immediately after that oxy-indole (1 equiv.) was added to the reaction mixture, gradually, in the course of 15–40 min, triethylamine (Et_3_N, 1 equiv.) dissolved in dry dichloromethane (2 mL) was added. The reaction mixture was stirred for the time and at the temperature specified in Table 2. After the completion of the reaction (Monitored by TLC), the mixture was transferred to a separatory funnel with dichloromethane (5–10 mL) and consecutively extracted with equal volumes of aqueous HCl (8%) and Na_2_CO_3_ (3%). The combined organic layer was dried (Na_2_SO_4_), and the solvent was removed under reduced pressure. Analytically pure samples were obtained through recrystallization or column chromatography on neutral aluminum oxide (Al_2_O_3_), using mixtures of diethyl ether/petroleum ether as eluents.

The synthesis of product **4e** was examined on a gram-scale scope, starting with 4,5-dimethylthiazole (3 mmol), Troc-Cl (3.6 mmol), and 5-methoxyindole (3 mmol). In the course of the reaction, triethylamine (3 mmol) was slowly added dropwise. At this scale, 1.255 g of the desired product was isolated by recrystallization with a mixture of petroleum/diethyl ether (96% yield).

Compound **4a** 2,2,2-trichloroethyl 2-(4-methoxy-1H-indol-3-yl)-4-methylthiazole-3 (2H)-carboxylate was isolated by recrystallization with a mixture of petroleum/diethyl ether (1:1), yield of 98%, melting point (m.p.) = 175–177 °C, and molecular weight (MW) = 421.72 g/mol.

**^1^H-NMR** (600 MHz, 80 °C, DMSO-d_6_, *δ* ppm, *J* Hz): 2.50 (s, 3H, -CH_3_), 4.02 (s, 3H, -OCH_3_), 4.98 (d, *J* = 12.3, 1H, -COOCH_2_C (Cl)_3_), 5.11 (d, *J* = 12.3, 1H, -COOCH_2_C (Cl)_3_), 5.79 (brs, 1H, -CH), 6.66 (d, *J* = 7.0, 1H, -CH), 7.08 (d, *J* = 1.8, 1H, -CH), 7.12 (d, *J* = 8.2, 1H, -CH), 7.17 (t, *J* = 7.6, 1H, -CH), 7.35 (s, 1H, *CH), 10.89 (s, 1H, -NH).

**^13^C{^1^H}-NMR** (150 MHz, 80 °C, DMSO-d_6_, *δ* ppm): 16.7 (-CH_3_), 55.9 (OCH_3_), 64.2 (*CH), 75.1 (COOCH_2_C (Cl)_3_), 96.1 (COOCH_2_C (Cl)_3_), 100.3 (C-Ar), 104.5 (C-Ar), 105.6 (C-Ar), 115.1 (C-Ar), 116.6 (C-Ar), 119.7 (C-Ar), 123.0 (C-Ar), 131.1 (C-Ar), 138.9 (C-Ar), 151.9 (C=O), 154.3 (C-O).

**FTIR** (KBr, cm^−1^): ν (N-H)—3378; ν (C=O)—1702; ν (Csp^2^-H)—2925, 2950; ν (C
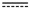
C)—1435, 1507, 1587; ν (C-O)—1039, 1083, 1261; ν (C-O-C)—1331; ν (C-N)—814, 852, 1389; ν (C-S-C)—730.

**HRMS** *m*/*z* (ESI): calcd for C_16_H_15_Cl_3_N_2_NaO_3_S^+^ [M + Na]^+^ 442.9761, found 442.9764.

Compound **4b** 2,2,2-trichloroethyl 2-(4-methoxy-1H-indol-3-yl)-4,5-dimethylthiazole-3(2H)-carboxylate was isolated by recrystallization with a mixture of petroleum/diethyl ether (8:1), yield of 97%, m.p. = 191–193 °C, and MW = 435.74 g/mol.

**^1^H-NMR** (600 MHz, 80 °C, DMSO-d_6_, *δ* ppm, *J* Hz): 2.04 (s, 3H, -CH_3_), 2.50 (s, 3H, -CH_3_), 4.10 (s, 3H, -OCH_3_), 5.04 (d, *J* = 12.3, 1H, -COOCH_2_C (Cl)_3_), 5.20 (d, *J* = 12.3, 1H, -COOCH_2_C (Cl)_3_), 6.73 (d, *J* = 7.6, 1H, -CH), 7.13 (d, *J* = 2.4, 1H, -CH), 7.19 (d, *J* = 8.2, 1H, -CH), 7.24 (t, *J* = 7.6, 1H, -CH), 7.31 (s, 1H, *CH), 10.94 (s, 1H, -NH).

**^13^C{^1^H}-NMR** (150 MHz, 80 °C, DMSO-d_6_, *δ* ppm): 13.4 (-CH_3_), 14.2 (-CH_3_), 55.9 (OCH_3_), 62.5 (*CH), 75.0 (COOCH_2_C (Cl)_3_), 96.2 (COOCH_2_C (Cl)_3_), 100.3 (C-Ar), 105.6 (C-Ar), 114.4 (C-Ar), 115.3 (C-Ar), 116.6 (C-Ar), 119.6 (C-Ar), 123.0 (C-Ar), 124.5 (C-Ar), 138.9 (C-Ar), 152.1 (C=O), 154.3 (C-O).

**FTIR** (KBr, cm^−1^): ν (N-H)—3369; ν (C=O)—1704; ν (Csp^2^-H)—2926, 2949; ν (C
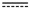
C)—1434, 1508, 1587; ν (C-O)—1039, 1083, 1260; ν (C-O-C)—1336; ν (C-N)—837, 1399; ν (C-S-C)—732.

**HRMS** *m*/*z* (ESI): calcd for C_17_H_17_Cl_3_N_2_NaO_3_S^+^ [M + Na]^+^ 456.9918, found 456.9915.

Compound **4c** 2,2,2-trichloroethyl 2-(5-methoxy-1H-indol-3-yl)thiazole-3 (2H)-carboxylate was isolated and purified by column chromatography on neutral aluminum oxide (Al_2_O_3_) with mixtures of petroleum/diethyl ether (1:1), yield of 93%, m.p. = 162–164 °C, and MW = 407.69 g/mol.

**^1^H-NMR** (400 MHz, 80 °C, DMSO-d_6_, *δ* ppm, *J* Hz): 3.77 (s, 3H, -OCH_3_), 4.83 (s, 2H, -COOCH_2_C (Cl)_3_), 6.19 (d, *J* = 4.8 Hz, 1H, -CH), 6.72 (d, *J* = 4.8, 1H, -CH), 6.79 (dd, ^2^*J* = 8.8, ^3^*J* = 2.5, 1H, -CH), 6.95 (s, 1H, *CH), 7.27–7.32 (m, 2H, -2xCH), 10.76 (s, 1H, -NH).

**^13^C{^1^H}-NMR** (100 MHz, 80 °C, DMSO-d_6_, *δ* ppm): 56.1 (OCH_3_), 60.9 (*CH), 75.1 (COOCH_2_C (Cl)_3_), 95.9 (COOCH_2_C (Cl)_3_), 102.7 (C-Ar), 106.4 (C-Ar), 111.8 (C-Ar), 112.8 (C-Ar), 115.5 (C-Ar), 124.7 (C-Ar), 125.2 (C-Ar), 132.6 (C=O), 154.0 (C-O).

**ATR-FTIR** (cm^−1^): ν (N-H)—3364; ν (C=O)—1709; ν (Csp^2^-H)—3116, 3091; ν (C
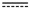
C)—1437, 1484, 1578; ν (C-O)—1035, 1066, 1129; ν (C-O-C)—1287; ν (C-N)—879, 1400; ν (C-S-C)—799.

**HRMS** *m*/*z* (ESI): calcd for C_15_H_13_Cl_3_N_2_NaO_3_S^+^ [M + Na]^+^ 428.9605, found 428.9609.

Compound **4d** 2,2,2-trichloroethyl 2-(5-methoxy-1H-indol-3-yl)-4-methylthiazole-3(2H)-carboxylate was isolated by recrystallization with petroleum ether, yield of 96%, m.p. = 122–124 °C, and MW = 421.72 g/mol.

**^1^H-NMR** (600 MHz, 80 °C, DMSO-d_6_, *δ* ppm, *J* Hz): 2.50 (s, 3H, -CH_3_), 3.96 (s, 3H, -OCH_3_), 5.01 (d, *J* = 11.7, 1H, -COOCH_2_C (Cl)_3_), 5.12 (d, *J* = 12.3, 1H, -COOCH_2_C (Cl)_3_), 6.13 (s, 1H, -CH), 6.97 (dd, ^2^*J* = 8.8, ^3^*J* = 2.4, 1H, -CH), 7.16 (s, 1H, *CH), 7.30 (s, 1H, -CH), 7.44 (d, *J* = 2.9, 1H, -CH), 7.46 (d, *J* = 8.8, 1H, -CH), 10.91 (s, 1H, -NH).

**^13^C{^1^H}-NMR** (150 MHz, 80 °C, DMSO-d_6_, *δ* ppm): 16.9 (CH_3_), 56.1 (OCH_3_), 63.1 (*CH), 75.2 (COOCH_2_C (Cl)_3_), 96.0 (COOCH_2_C (Cl)_3_), 102.3 (C-Ar), 105.5 (C-Ar), 112.0 (C-Ar), 112.9 (C-Ar), 116.1 (C-Ar), 124.3 (C-Ar), 125.1 (C-Ar), 130.7 (C-Ar), 132.6 (C-Ar), 151.9 (C=O), 154.1 (C-O).

**FTIR** (KBr, cm^−1^): ν (N-H)—3106; ν (C=O)—1629; ν (Csp^2^-H)—2884, 2922; ν (C
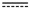
C)—1454, 1485, 1539; ν (C-O)—1035, 1083, 1290; ν (C-O-C)—1305; ν (C-N)—838, 1341; ν (C-S-C)—795.

**HRMS** *m*/*z* (ESI): calcd for C_16_H_15_Cl_3_N_2_NaO_3_S^+^ [M + Na]^+^ 442.9761, found 442.9764.

Compound **4e** 2,2,2-trichloroethyl 2-(5-methoxy-1*H*-indol-3-yl)-4,5-dimethylthiazole-3(2H)-carboxylate was isolated by recrystallization with a mixture of petroleum/diethyl ether (8:1), yield of 98%, m.p. = 143–145 °C, and MW = 435.74 g/mol.

**^1^H-NMR** (600 MHz, 80 °C, DMSO-d_6_, *δ* ppm, *J* Hz): 1.98 (s, 3H, -CH_3_), 2.23 (s, 3H, -CH_3_), 3.76 (s, 3H, -OCH_3_), 4.81 (d, *J* = 12.3, 1H, -COOCH_2_C (Cl)_3_), 4.96 (d, *J* = 12.3, 1H, -COOCH_2_C (Cl)_3_), 6.77 (dd, ^2^*J* = 8.8, ^3^*J* = 2.4, 1H, -CH), 6.85 (s, 1H, *CH), 7.01 (d, *J* = 2.4, 1H, -CH), 7.24 (d, *J* = 2.4, 1H, -CH), 7.27 (d, *J* = 8.8, 1H, -CH), 10.70 (s, 1H, -NH).

**^13^C{^1^H}-NMR** (150 MHz, 80 °C, DMSO-d_6_, *δ* ppm): 13.3 (-CH_3_), 14.3 (-CH_3_), 56.1 (OCH_3_), 61.5 (*CH), 75.2 (COOCH_2_C (Cl)_3_), 96.1 (COOCH_2_C (Cl)_3_), 101.9 (C-Ar), 112.1 (C-Ar), 112.9 (C-Ar), 115.6 (C-Ar), 116.0 (C-Ar), 124.3 (C-Ar), 124.4 (C-Ar), 125.2 (C-Ar), 132.6 (C-Ar), 152.1 (C=O), 154.1 (C-O).

**FTIR** (KBr, cm^−1^): ν (N-H)—3394; ν (C=O)—1736; ν (Csp^2^-H)—2950, 2999; ν (C
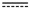
C)—1485, 1531, 1581; ν (C-O)—1026, 1063, 1280; ν (C-O-C)—1337; ν (C-N)—821, 842, 1395; ν (C-S-C)—732.

**HRMS** *m*/*z* (ESI): calcd for C_17_H_17_Cl_3_N_2_NaO_3_S^+^ [M + Na]^+^ 456.9918, found 456.9920.

Compound **4f** 2,2,2-trichloroethyl 2-(6-methoxy-1*H*-indol-3-yl)thiazole-3(2H)-carboxylate was isolated and purified by column chromatography on neutral aluminum oxide (Al_2_O_3_) with mixtures of petroleum/diethyl ether (1:1), yield of 96%, oil, and MW = 407.69 g/mol.

**^1^H-NMR** (400 MHz, 80 °C, DMSO-d_6_, *δ* ppm, *J* Hz): 3.78 (s, 3H, -OCH_3_), 4.82 (s, 2H, -COOCH_2_C (Cl)_3_), 6.15 (d, *J* = 4.7, 1H, -CH), 6.67–6.71 (m, 2H, -2xCH), 6.91 (d, *J* = 2.3, 1H, -CH), 6.92 (s, 1H, *CH), 7.15–7.26 (m, 1H, -CH), 7.49 (d, *J* = 8.30, 1H, -CH), 10.69 (s, 1H).

**^13^C{^1^H}-NMR** (100 MHz, 80 °C, DMSO-d_6_, *δ* ppm): 55.9 (OCH_3_), 60.9 (*CH), 75.1 (COOCH_2_C (Cl)_3_), 95.9 (COOCH_2_C (Cl)_3_), 106.5 (C-Ar), 109.7 (C-Ar), 110.2 (C-Ar), 115.7 (C-Ar), 119.1 (C-Ar), 120.2 (C-Ar), 120.6 (C-Ar), 122.9 (C-Ar), 123.3 (C-Ar), 138.2 (C=O), 156.5 (C-O).

**ATR-FTIR** (cm^−1^): ν (N-H)—3401; ν (C=O)—1699; ν (Csp^2^-H)—2948; ν (C
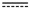
C)—1451, 1539; ν (C-O)—1025, 1129, 1160; ν (C-O-C)—1236; ν (C-N)—830, 1408; ν (C-S-C)—725.

**HRMS** *m*/*z* (ESI): calcd for C_15_H_13_Cl_3_N_2_NaO_3_S+ [M + Na]^+^ 428.9605, found 428.9608.

Compound **4g** 2,2,2-trichloroethyl 2-(6-methoxy-1*H*-indol-3-yl)-4,5-dimethylthiazole-3(2H)-carboxylate was isolated and purified by column chromatography on neutral aluminum oxide (Al_2_O_3_) with mixtures of petroleum/diethyl ether (8:1), yield of 93%, m.p. = 66–68 °C, and MW = 435.74 g/mol.

**^1^H-NMR** (400 MHz, 80 °C, DMSO-d_6_, *δ* ppm, *J* Hz): 1.96 (s, 3H, -CH_3_), 2.20 (s, 3H, -CH_3_), 3.78 (s, 3H, -OCH_3_), 4.83 (d, *J* = 12.3, 1H, -COOCH_2_C (Cl)_3_), 4.97 (d, *J* = 12.3, 1H, -COOCH_2_C (Cl)_3_), 6.70 (dd, ^2^*J* = 8.7, ^3^*J* = 2.3, 1H, -CH), 6.84 (s, 1H, *CH), 6.91 (d, *J* = 2.2, 1H, -CH), 7.15 (d, *J* = 2.5, 1H, -CH), 7.39 (d, *J* = 8.7, 1H, -CH), 10.65 (s, 1H, -NH).

**^13^C{^1^H}-NMR** (100 MHz, 80 °C, DMSO-d_6_, *δ* ppm): 13.4 (-CH_3_), 14.3 (-CH_3_), 55.9 (OCH_3_), 61.5 (*CH), 75.1 (COOCH_2_C (Cl)_3_), 96.0 (COOCH_2_C (Cl)_3_), 96.1 (C-Ar), 109.7 (C-Ar), 115.6 (C-Ar), 116.1 (C-Ar), 119.3 (C-Ar), 119.9 (C-Ar), 122.4 (C-Ar), 124.3 (C-Ar), 138.3 (C-Ar), 152.1 (C=O), 156.4 (C-O).

**ATR-FTIR** (cm^−1^): ν (N-H)—3413; ν (C=O)—1701; ν (Csp^2^-H)—2944; ν (C
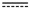
C)—1451, 1539; ν (C-O)—1025, 1102, 1158; ν (C-O-C)—1236; ν (C-N)—824, 1408; ν (C-S-C)—725.

**HRMS** *m*/*z* (ESI): calcd for C_17_H_17_Cl_3_N_2_NaO_3_S^+^ [M + Na]^+^ 456.9918, found 456.9913.

Compound **4h** 2,2,2-trichloroethyl 2-(4,6-dimethoxy-1*H*-indol-3-yl)-4-methylthiazole-3(2H)-carboxylate: isolated by column chromatography on neutral aluminum oxide (Al_2_O_3_) with mixtures of petroleum/diethyl ether (1:1), yield of 92%, m.p. = 171–173 °C, and MW = 451.74 g/mol.

**^1^H-NMR** (600 MHz, 80 °C, DMSO-d_6_, *δ* ppm, *J* Hz): 2.50 (s, 3H, -CH_3_), 3.93 (s, 3H, -OCH_3_), 4.01 (s, 3H, -OCH_3_), 4.99 (d, *J* = 12.3, 1H, -COOCH_2_C (Cl)_3_), 5.12 (d, *J* = 12.3, 1H, -COOCH_2_C (Cl)_3_), 5.80 (s, 1H, -CH), 6.36 (d, *J* = 1.7, 1H, -CH), 6.65 (d, *J* = 2.4, 1H, -CH), 6.96 (d, *J* = 2.4, 1H, -CH), 7.29 (s, 1H, *CH), 10.68 (s, 1H, -NH).

**^13^C{^1^H}-NMR** (150 MHz, 80 °C, DMSO-d_6_, *δ* ppm): 16.7 (-CH_3_), 55.9 (OCH_3_), 56.0 (OCH_3_), 64.1 (*CH), 75.1 (COOCH_2_C (Cl)_3_), 88.6 (COOCH_2_C (Cl)_3_), 92.2 (C-Ar), 96.1 (C-Ar), 104.6 (C-Ar), 109.8 (C-Ar), 116.5 (C-Ar), 118.4 (C-Ar), 131.1 (C-Ar), 138.9 (C-Ar), 151.9 (C=O), 154.5 (C-O), 157.7 (C-O).

**FTIR** (KBr, cm^−1^): ν (N-H)—3372; ν (C=O)—1685; ν (Csp^2^-H)—2935, 3005; ν (C
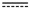
C)—1449, 1539, 1586; ν (C-O)—1036, 1091, 1263; ν (C-O-C)—1325; ν (C-N)—806, 827, 1399; ν (C-S-C)—717.

**HRMS** *m*/*z* (ESI): calcd for C_17_H_17_Cl_3_N_2_NaO_4_S^+^ [M + Na]^+^ 472.9867, found 472.9869.

Compound **4i** 2,2,2-trichloroethyl 2-(4,6-dimethoxy-1*H*-indol-3-yl)-4,5-dimethylthiazole-3(2H)-carboxylate was isolated and purified by column chromatography on neutral aluminum oxide (Al_2_O_3_) with mixtures of petroleum/diethyl ether (8:1), yield of 96%, m.p. = 183–185 °C, and MW = 465.77 g/mol.

**^1^H-NMR** (600 MHz, 80 °C, DMSO-d_6_, *δ* ppm, *J* Hz): 2.05 (s, 3H, -CH_3_), 2.50 (s, 3H, -CH_3_), 4.01 (s, 3H, -OCH_3_), 4.08 (s, 3H, -OCH_3_), 5.05 (d, *J* = 12.3, 1H, -COOCH_2_C (Cl)_3_), 5.21 (d, *J* = 12.3, 1H, -COOCH_2_C (Cl)_3_), 6.43 (d, *J* = 1.8, 1H, -CH), 6.72 (d, *J* = 1.8, 1H, -CH), 7.01 (d, *J* = 1.8, 1H, -CH), 7.25 (s, 1H, *CH), 10.73 (s, 1H, -NH).

**^13^C{^1^H}-NMR** (150 MHz, 80 °C, DMSO-d_6_, *δ* ppm): 13.5 (-CH_3_), 14.2 (-CH_3_), 55.9 (OCH_3_), 56.0 (OCH_3_), 62.5 (*CH), 75.0 (COOCH_2_C (Cl)_3_), 88.6 (COOCH_2_C (Cl)_3_), 92.2 (C-Ar), 96.2 (C-Ar), 109.9 (C-Ar), 114.5 (C-Ar), 116.6 (C-Ar), 118.3 (C-Ar), 124.5 (C-Ar), 138.9 (C-Ar), 152.1 (C=O), 154.5 (C-O), 157.7 (C-O).

**FTIR** (KBr, cm^−1^): ν (N-H)—3406; ν (C=O)—1706; ν (Csp^2^-H)—2932, 2963; ν (C
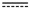
C)—1434, 1512, 1592; ν (C-O)—1036, 1090, 1270; ν (C-O-C)—1323; ν (C-N)—819, 1409; ν (C-S-C)—720.

**HRMS** *m*/*z* (ESI): calcd for C_18_H_19_Cl_3_N_2_NaO_4_S^+^ [M + Na]^+^ 487.0023, found 487.0027.

Compound **4j** 2,2,2-trichloroethyl 2-(4- (benzyloxy)-1*H*-indol-3-yl)thiazole-3(2H)-carboxylate was isolated and purified by column chromatography on neutral aluminum oxide (Al_2_O_3_) with mixtures of petroleum/diethyl ether (4:1), yield of 97%, m.p. = 54–56 °C, and MW = 483.79 g/mol.

**^1^H-NMR** (400 MHz, 80 °C, DMSO-d_6_, *δ* ppm, *J* Hz): 4.77–4.98 (m, 2H, -COOCH_2_C (Cl)_3_), 5.23 (s, 2H, -OCH_2_Ph), 5.92 (d, *J* = 4.1, 1H, -CH), 6.62 (dd, ^2^*J* = 7.0, ^3^*J* = 1.5, 1H, -CH), 6.70 (d, *J* = 4.6, 1H, -CH), 6.98–7.04 (m, 3H, -3×CH), 7.16 (s, 1H, *CH), 7.34 (t, *J* = 7.3, 1H, -CH), 7.42 (t, *J* = 7.2, 2H, -2×CH), 7.55 (d, *J* = 6.8, 2H, -2×CH), 10.84 (s, 1H, -NH).

**^13^C{^1^H}-NMR** (100 MHz, 80 °C, DMSO-d_6_, *δ* ppm): 61.7 (*CH), 70.1 (CH_2_Ph), 75.0 (COOCH_2_C (Cl)_3_), 96.0 (COOCH_2_C (Cl)_3_), 101.4 (C-Ar), 105.7 (C-Ar), 105.8 (C-Ar), 115.0 (C-Ar), 116.6 (C-Ar), 120.0 (C-Ar), 120.9 (C-Ar), 123.0 (C-Ar), 127.9 (C-Ar), 128.1 (C-Ar), 128.8 (C-Ar), 137.8 (C-Ar), 138.9 (C=O), 141.8 (C-Ar), 142.5 (C-Ar), 153.2 (C-O).

ATR-FTIR (cm^−1^): ν (N-H)—3401; ν (C=O)—1722; ν (Csp^2^-H)—2964; ν (C
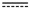
C)—1504, 1586; ν (C-O)—1090, 1117, 1164; ν (C-O-C)—1240; ν (C-N)—879, 1400; ν (C-S-C)—731.

**HRMS** *m*/*z* (ESI): calcd for C_21_H_17_Cl_3_N_2_NaO_3_S^+^ [M + Na]^+^ 504.9918, found 504.9920.

Compound **4k** 2,2,2-trichloroethyl 2-(4-(benzyloxy)-1*H*-indol-3-yl)-4,5-dimethylthiazole-3(2H)-carboxylate was isolated and purified by column chromatography on neutral aluminum oxide (Al_2_O_3_) with mixtures of petroleum/diethyl ether (1:1), yield of 93%, m.p. = 141–143 °C, and MW = 511.84 g/mol.

**^1^H-NMR** (400 MHz, 80 °C, DMSO-d_6_, *δ* ppm, *J* Hz): 1.81 (s, 3H, -CH_3_), 2.28 (s, 3H, -CH_3_), 4.76 (d, *J* = 12.2, 1H, -COOCH_2_C (Cl)_3_), 4.99 (d, *J* = 12.2, 1H, -COOCH_2_C (Cl)_3_), 5.21 (s, 2H, -OCH_2_Ph), 6.61 (dd, ^2^*J* = 6.7, ^2^*J* = 1.8, 1H, -CH), 6.93 (d, *J* = 2.5, 1H, -CH), 6.96–7.06 (m, 2H, -2xCH), 7.13 (s, 1H, *CH), 7.34 (t, *J* = 7.3, 1H, -CH), 7.41 (t, *J* = 7.2, 2H, -2xCH), 7.55 (d, *J* = 6.8, 2H, -2xCH), 10.77 (s, 1H, -NH).

**^13^C{^1^H}-NMR** (100 MHz, 80 °C, DMSO-d_6_, *δ* ppm): 13.4 (CH_3_), 14.1 (CH_3_), 62.5 (*CH), 70.1 (CH_2_Ph), 74.9 (COOCH_2_C (Cl)_3_), 96.1 (COOCH_2_C (Cl)_3_), 101.2 (C-Ar), 105.7 (C-Ar), 114.3 (C-Ar), 115.2 (C-Ar), 116.7 (C-Ar), 119.7 (C-Ar), 122.9 (C-Ar), 124.6 (C-Ar), 127.8 (C-Ar), 128.1 (C-Ar), 128.8 (C-Ar), 137.8 (C-Ar), 139.0 (C-Ar), 151.9 (C=O), 153.2 (C-O).

**ATR-FTIR** (cm^−1^): ν (N-H)—3388; ν (C=O)—1703; ν (Csp^2^-H)—2964; ν (C
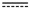
C)—1500, 1541; ν (C-O)—1092, 1135, 1191; ν (C-O-C)—1226; ν (C-N)—828, 1404; ν (C-S-C)—736.

**HRMS** *m*/*z* (ESI): calcd for C_23_H_21_Cl_3_N_2_NaO_3_S^+^ [M + Na]^+^ 533.0231, found 533.0233.

Compound **4l** 2,2,2-trichloroethyl 2-(5-hydroxy-1*H*-indol-3-yl)-4,5-dimethylthiazole-3(2H)-carboxylate was isolated and purified by column chromatography on silica gel with mixtures of petroleum/diethyl ether (4:1), yield of 77%, oil, and MW = 421.72 g/mol.

**^1^H-NMR** (400 MHz, 80 °C, DMSO-d_6_, *δ* ppm, *J* Hz): 1.97 (s, 3H, -CH_3_), 2.22 (s, 3H, -CH_3_), 4.82 (d, *J* = 12.2, 1H, -COOCH_2_C (Cl)_3_), 4.97 (d, *J* = 12.2, 1H, -COOCH_2_C (Cl)_3_), 6.65 (dd, ^2^*J* = 8.7, ^3^*J* = 2.4, 1H, -CH), 6.80 (s, 1H, *CH), 6.84 (d, *J* = 2.4, 1H, -CH), 7.17 (d, *J* = 9.3, 2H, -2xCH), 8.42 (s, 1H, -OH), 10.55 (s, 1H, -NH).

**^13^C{^1^H}-NMR** (100 MHz, 80 °C, DMSO-d_6_, *δ* ppm): 13.4 (CH_3_), 14.4 (CH_3_), 61.6 (*CH), 75.1 (COOCH_2_C (Cl)_3_), 96.1 (COOCH_2_C (Cl)_3_), 103.9 (C-Ar), 112.4 (C-Ar), 112.5 (C-Ar), 115.2 (C-Ar), 115.5 (C-Ar), 123.9 (C-Ar), 124.3 (C-Ar), 125.5 (C-Ar), 132.0 (C-Ar), 151.2 (C=O), 152.1 (C-O).

**ATR-FTIR** (cm^−1^): ν (O-H)—3400; ν (C=O)—1703; ν (Csp^2^-H)—2955, 2920; ν (C
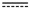
C)—1455, 1582; ν (C-O)—1053, 1096, 1149; ν (C-O-C)—1186, 1340; ν (C-N)—801, 1399; ν (C-S-C)—719.

**HRMS** *m*/*z* (ESI): calcd for C_16_H_15_Cl_3_N_2_NaO_3_S^+^ [M + Na]^+^ 442.9761, found 442.9760.

#### 4.1.3. General Procedure for Oxidative Rearomatization of Compounds **4** to Oxy-Camalexins **5**

The corresponding *N*-acylated compound **4a–l** (0.5 mmol) was dissolved in acetonitrile (CH_3_CN, 8 mL) or dichloromethane (CH_2_Cl_2_, 8 mL, for DDQ examples), and then the corresponding oxidant *o*-chloranil (0.5 mmol) or DDQ (0.5 mmol) was added. Then, the reaction mixture was magnetically stirred under the conditions specified in Table 3. After the completion of the reaction (monitored by TLC), the solvent was evaporated under reduced pressure, and the mixture was then dry-loaded onto neutral aluminum oxide (Al_2_O_3_) or silica gel. Chromatography on a short column with diethyl ether/petroleum as the eluent gave oxy-camalexins in yields indicated in Table 3.

From compound **4e**, synthesized on a gram-scale, 3 mmol was taken, and oxidative rearomatization was carried out with an equimolar amount of *o*-chloranil in acetonitrile. Here, the reaction was also found to work with an excellent yield of 90% for oxy-camalexin **5e** (0.6975 g).

Oxidative rearomatization of compound **4a** 2,2,2-trichloroethyl 2-(4-methoxy-1*H*-indol-3-yl)-4-methylthiazole-3(2H)-carboxylate. The synthesized compound **5a** 2-(4-methoxy-1*H*-indol-3-yl)-4-methylthiazole was isolated and purified by column chromatography on neutral aluminum oxide (Al_2_O_3_) with mixtures of petroleum/diethyl ether (1:1), yield of 86%, m.p. = 180–181 °C, and MW = 244.31 g/mol.

**^1^H-NMR** (600 MHz, 25 °C, DMSO-d_6_, *δ* ppm, *J* Hz): 2.50 (s, 3H, -CH_3_), 4.03 (s, 3H, -OCH_3_), 6.74 (d, *J* = 7.6, 1H, -CH), 7.18 (d, *J* = 8.2, 1H, -CH), 7.21 (brs, 1H, -CH), 7.23 (t, *J* = 7.6, 1H, -CH), 8.00 (s, 1H, -CH), 11.81 (s, 1H, -NH).

**^13^C{^1^H}-NMR** (150 MHz, 25 °C, DMSO-d_6_, *δ* ppm): 17.4 (-CH_3_), 55.1 (OCH_3_), 101.1 (C-Ar), 105.9 (C-Ar), 111.4 (C-Ar), 113.7 (C-Ar), 114.4 (C-Ar), 123.4 (C-Ar), 126.0 (C-Ar), 138.6 (C-Ar), 151.4 (C-Ar), 153.6 (C-O), 162.6 (S-C=N).

**FTIR** (KBr, cm^−1^): ν (N-H)—3108; ν (Csp^2^-H)—2916, 2967; ν (C
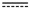
C)—1464, 1523, 1589; ν (C-O)—1109, 1252; ν (C-O-C)—1325; ν (C-N)—781, 1362; ν (C-S-C)—737.

**HRMS** *m*/*z* (ESI): calcd for C_13_H_11_N_2_OS^-^ [M-H]^−^ 243.0598, found 243.0595.

Oxidative rearomatization of compound **4b** 2,2,2-trichloroethyl 2-(4-methoxy-1*H*-indol-3-yl)-4,5-dimethylthiazole-3(2H)-carboxylate. The synthesized compound **5b** 2-(4-methoxy-1*H*-indol-3-yl)-4,5-dimethylthiazole was isolated and purified by column chromatography on neutral aluminum oxide (Al_2_O_3_) with mixtures of petroleum/diethyl ether (1:1), yield of 80%, m.p. = 212–214 °C, and MW = 258.34 g/mol.

**^1^H-NMR** (600 MHz, 25 °C, DMSO-d_6_, *δ* ppm, *J* Hz): 2.50 (s, 3H, -CH_3_), 2.58 (s, 3H, -CH_3_), 4.13 (s, 3H, -OCH_3_), 6.84 (d, *J* = 7.0, 1H, -CH), 7.27 (d, *J* = 7.6, 1H, -CH), 7.32 (t, *J* = 7.6, 1H, -CH), 8.03 (d, *J* = 2.9, 1H, -CH), 11.85 (s, 1H, -NH).

**^13^C{^1^H}-NMR** (150 MHz, 25 °C, DMSO-d_6_, *δ* ppm): 11.2 (-CH_3_), 15.0 (-CH_3_), 55.1 (OCH_3_), 101.0 (C-Ar), 105.9 (C-Ar), 111.4 (C-Ar), 114.4 (C-Ar), 123.3 (C-Ar), 125.2 (C-Ar), 125.4 (C-Ar), 138.5 (C-Ar), 146.9 (C-Ar), 153.6 (C-O), 158.5 (S-C=N).

**FTIR** (KBr, cm^−1^): ν (N-H)—3083; ν (Csp^2^-H)—2857, 2913; ν (C
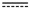
C)—1460, 1532, 1589; ν (C-O)—1093, 1252; ν (C-O-C)—1326; ν (C-N)—776, 1363; ν (C-S-C)—723.

**HRMS** *m*/*z* (ESI): calcd for C_14_H_13_N_2_OS^-^ [M-H]^−^ 257.0754, found 257.0757.

Oxidative rearomatization of compound **4c** 2,2,2-trichloroethyl 2-(5-methoxy-1*H*-indol-3-yl)thiazole-3(2H)-carboxylate. The synthesized compound **5c** 2-(5-methoxy-1*H*-indol-3-yl)thiazole was isolated and purified by column chromatography on neutral aluminum oxide (Al_2_O_3_) with mixtures of petroleum/diethyl ether (1:1), yield of 98%, m.p. = 112–114 °C, and MW = 230.29 g/mol.

**^1^H-NMR** (400 MHz, 25 °C, DMSO-d_6_, *δ* ppm, *J* Hz): 3.82 (s, 3H, -OCH_3_), 6.87 (dd, ^2^*J* = 8.7, ^2^*J* = 2.5, 1H, -CH), 7.38 (d, *J* = 9.5, 1H, -CH), 7.52 (d, *J* = 3.3, 1H, -CH), 7.70 (d, *J* = 3.0, 1H, -CH), 7.82 (d, *J* = 3.4, 1H, -CH), 8.01 (d, *J* = 2.8, 1H, -CH), 11.57 (s, 1H, -NH).

^1**3**^**C{^1^H}-NMR** (100 MHz, 25 °C, DMSO-d_6_, *δ* ppm): 55.8 (OCH_3_), 102.4 (C-Ar), 110.9 (C-Ar), 112.9 (C-Ar), 113.3 (C-Ar), 116.3 (C-Ar), 125.2 (C-Ar), 127.3 (C-Ar), 132.0 (C-Ar), 143.0 (C-Ar), 155.1 (C-O), 163.6 (S-C=N).

**ATR-FTIR** (cm^−1^): ν (N-H)—3398; ν (Csp^2^-H)—2933; ν (C
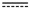
C)—1475, 1541, 1586; ν (C-O)—1076, 1213; ν (C-O-C)—1295; ν (C-N)—805, 867; ν (C-S-C)—715.

**HRMS** *m*/*z* (ESI): calcd for C_12_H_9_N_2_OS^-^ [M-H]^−^ 229.0441, found 229.0444.

Oxidative rearomatization of compound **4d** 2,2,2-trichloroethyl 2-(5-methoxy-1*H*-indol-3-yl)-4-methylthiazole-3(2H)-carboxylate. The synthesized compound **5d** 2-(5-methoxy-1*H*-indol-3-yl)-4-methylthiazole was isolated and purified by column chromatography on neutral aluminum oxide (Al_2_O_3_) with mixtures of petroleum/diethyl ether (1:1), yield of 90%, oil, and MW = 244.31 g/mol.

**^1^H-NMR** (600 MHz, 25 °C, DMSO-d_6_, *δ* ppm, *J* Hz): 2.50 (s, 3H, -CH_3_), 3.89 (s, 3H, -OCH_3_), 6.95 (dd, ^2^*J* = 8.8, ^3^*J* = 2.4, 1H, -CH), 7.14 (s, 1H, -CH), 7.46 (d, *J* = 8.8, 1H, -CH), 7.73 (d, *J* = 2.4, 1H, -CH), 8.06 (s, 1H, -CH), 11.65 (s, 1H, -NH).

**^13^C{^1^H}-NMR** (150 MHz, 25 °C, DMSO-d_6_, *δ* ppm): 17.5 (-CH_3_), 55.8 (OCH_3_), 102.6 (C-Ar), 110.5 (C-Ar), 111.0 (C-Ar), 112.6 (C-Ar), 113.3 (C-Ar), 125.2 (C-Ar), 127.0 (C-Ar), 132.0 (C-Ar), 155.0 (C-Ar), 160.0 (C-O), 162.6 (S-C=N).

**FTIR** (KBr, cm^−1^): ν (N-H)—3112; ν (Csp^2^-H)—2912, 2970; ν (C
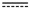
C)—1472, 1525, 1592; ν (C-O)—1112, 1255; ν (C-O-C)—1315; ν (C-N)—803, 1363; ν (C-S-C)—742.

**HRMS** *m*/*z* (ESI): calcd for C_13_H_11_N_2_OS^-^ [M-H]^−^ 243.0598, found 243.0599.

Oxidative rearomatization of compound **4e** 2,2,2-trichloroethyl 2-(5-methoxy-1*H*-indol-3-yl)-4,5-dimethylthiazole-3(2H)-carboxylate. The synthesized compound **5e** 2-(5-methoxy-1*H*-indol-3-yl)-4,5-dimethylthiazole was isolated and purified by column chromatography on neutral aluminum oxide (Al_2_O_3_) with mixtures of petroleum/diethyl ether (1:1), yield of 91%, m.p. = 169–170 °C, and MW = 258.34 g/mol.

**^1^H-NMR** (600 MHz, 25 °C, DMSO-d_6_, *δ* ppm, *J* Hz): 2.50 (s, 3H, -CH_3_), 2.54 (s, 3H, -CH_3_), 3.99 (s, 3H, -OCH_3_), 7.04 (dd, ^2^*J* = 8.8, ^3^*J* = 2.4, 1H, -CH), 7.55 (d, *J* = 8.8, 1H, -CH), 7.80 (d, *J* = 2.4, 1H, -CH), 8.07 (d, *J* = 2.9, 1H, -CH), 11.68 (s, 1H, -NH).

**^13^C{^1^H}-NMR** (150 MHz, 25 °C, DMSO-d_6_, *δ* ppm): 11.3 (-CH_3_), 15.1 (-CH_3_), 55.8 (OCH_3_), 102.5 (C-Ar), 111.0 (C-Ar), 112.6 (C-Ar), 113.3 (C-Ar), 122.2 (C-Ar), 125.1 (C-Ar), 126.4 (C-Ar), 132.0 (C-Ar), 147.6 (C-Ar), 154.9 (C-O), 158.8 (S-C=N).

**FTIR** (KBr, cm^−1^): ν (N-H)—3140; ν (Csp^2^-H)—2880, 2919; ν (C
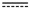
C)—1487, 1538, 1579; ν (C-O)—1079, 1211; ν (C-O-C)—1292; ν (C-N)—801, 1313; ν (C-S-C)—714.

**HRMS** *m*/*z* (ESI): calcd for C_14_H_13_N_2_OS^-^ [M-H]^−^ 257.0754, found 257.0750.

Oxidative rearomatization of compound **4f** 2,2,2-trichloroethyl 2-(6-methoxy-1*H*-indol-3-yl)thiazole-3(2H)-carboxylate. The synthesized compound **5f** 2-(6-methoxy-1*H*-indol-3-yl)thiazole was isolated and purified by column chromatography on neutral aluminum oxide (Al_2_O_3_) with mixtures of petroleum/diethyl ether (1:1), yield of 78%, m.p. = 161–163 °C, and MW = 230.29 g/mol.

**^1^H-NMR** (400 MHz, 25 °C, DMSO-d_6_, *δ* ppm, *J* Hz): 3.80 (s, 3H, -OCH_3_), 6.84 (dd, ^2^*J* = 8.7, ^3^*J* = 2.3, 1H, -CH), 6.97 (d, *J* = 2.3, 1H, -CH), 7.51 (d, *J* = 3.3, 1H, -CH), 7.79 (d, *J* = 3.3, 1H, -CH), 7.92 (s, 1H, -CH), 8.05 (d, *J* = 8.7, 1H, -CH), 11.48 (s, 1H, -NH).

**^13^C{^1^H}-NMR** (100 MHz, 25 °C, DMSO-d_6_, *δ* ppm): 55.7 (OCH_3_), 95.4 (C-Ar), 111.1 (C-Ar), 111.2 (C-Ar), 116.5 (C-Ar), 119.0 (C-Ar), 121.3 (C-Ar), 125.5 (C-Ar), 137.8 (C-Ar), 143.0 (C-Ar), 156.6 (C-O), 163.5 (S-C=N).

**ATR-FTIR** (cm^−1^): ν (N-H)—3163; ν (Csp^2^-H)—2905; ν (C
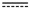
C)—1459, 1543, 1625; ν (C-O)—1084, 1170; ν (C-O-C)—1201; ν (C-N)—801, 828; ν (C-S-C)—727.

**HRMS** *m*/*z* (ESI): calcd for C_12_H_9_N_2_OS^-^ [M-H]^−^ 229.0441, found 229.0447.

Oxidative rearomatization of compound **4g** 2,2,2-trichloroethyl 2-(6-methoxy-1*H*-indol-3-yl)-4,5-dimethylthiazole-3(2H)-carboxylate. The synthesized compound **5g** 2-(6-methoxy-1*H*-indol-3-yl)-4,5-dimethylthiazole was isolated and purified by column chromatography on neutral aluminum oxide (Al_2_O_3_) with mixtures of petroleum/diethyl ether (1:1), yield of 91%, m.p. = 163–165 °C, and MW = 258.34 g/mol.

**^1^H-NMR** (400 MHz, 25 °C, DMSO-d_6_, *δ* ppm, *J* Hz): 2.30 (s, 3H, -CH_3_), 2.35 (s, 3H, -CH_3_), 3.79 (s, 3H, -OCH_3_), 6.81 (dd, ^2^*J* = 8.7, ^3^*J* = 2.4, 1H, -CH), 6.94 (d, *J* = 2.3, 1H, -CH), 7.78 (d, *J* = 2.6, 1H, -CH), 7.99 (d, *J* = 8.7, 1H, -CH), 11.38 (s, 1H, -NH).

**^13^C{^1^H}-NMR** (100 MHz, 25 °C, DMSO-d_6_, *δ* ppm): 11.3 (CH_3_), 15.1 (CH_3_), 55.7 (OCH_3_), 95.3 (C-Ar), 111.0 (C-Ar), 111.2 (C-Ar), 119.0 (C-Ar), 121.4 (C-Ar), 122.4 (C-Ar), 124.6 (C-Ar), 137.8 (C-Ar), 147.7 (C-Ar), 156.6 (C-Ar), 158.8 (C-Ar).

**ATR-FTIR** (cm^−1^): ν (N-H)—3224; ν (Csp^2^-H) –2829, 2919; ν (C
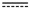
C)—1451, 1543, 1623; ν (C-O)—1084, 1164; ν (C-O-C)—1199; ν (C-N)—795, 830; ν (C-S-C)—746.

**HRMS** *m*/*z* (ESI): calcd for C_14_H_13_N_2_OS^-^ [M-H]^−^ 257.0754, found 257.0759.

Oxidative rearomatization of compound **4h** 2,2,2-trichloroethyl 2-(4,6-dimethoxy-1*H*-indol-3-yl)-4-methylthiazole-3(2H)-carboxylate. The synthesized compound **5h** 2-(4,6-dimethoxy-1*H*-indol-3-yl)-4-methylthiazole was isolated and purified by column chromatography on neutral aluminum oxide (Al_2_O_3_) with mixtures of petroleum/diethyl ether (1:1), yield of 68%, m.p. = 167–169 °C, and MW = 274.34 g/mol.

**^1^H-NMR** (600 MHz, 25 °C, DMSO-d_6_, *δ* ppm, *J* Hz): 2.50 (s, 3H, -CH_3_), 3.92 (s, 3H, -OCH_3_), 4.01 (s, 3H, -OCH_3_), 6.41 (d, *J* = 2.4, 1H, -CH), 6.68 (d, *J* = 1.8, 1H, -CH), 7.20 (s, 1H, -CH), 7.86 (d, *J* = 2.4, 1H, -CH), 11.60 (s, 1H, -NH).

**^13^C{^1^H}-NMR** (150 MHz, 25 °C, DMSO-d_6_, *δ* ppm): 17.4 (-CH_3_), 55.1 (OCH_3_), 55.7 (OCH_3_), 87.9 (C-Ar), 92.8 (C-Ar), 109.0 (C-Ar), 111.4 (C-Ar), 113.4 (C-Ar), 124.6 (C-Ar), 138.8 (C-Ar), 151.3 (C-O), 153.9 (C-Ar), 157.5 (S-C=N), 162.6 (C-O).

**FTIR** (KBr, cm^−1^): ν (N-H)—3109; ν (Csp^2^-H)—2938, 2956; ν (C
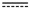
C)—1454, 1527, 1587; ν (C-O)—1113, 1160, 1201; ν (C-O-C)—1330; ν (C-N)—811, 1373; ν (C-S-C)—725.

**HRMS** *m*/*z* (ESI): calcd for C_14_H_13_N_2_O_2_S^-^ [M-H]^−^ 273.0703, found 273.0705.

Oxidative rearomatization of compound **4i** 2,2,2-trichloroethyl 2-(4,6-dimethoxy-1*H*-indol-3-yl)-4,5-dimethylthiazole-3(2H)-carboxylate. The synthesized compound **5i** 2-(4,6-dimethoxy-1*H*-indol-3-yl)-4,5-dimethylthiazole was isolated and purified by column chromatography on neutral aluminum oxide (Al_2_O_3_) with mixtures of petroleum/diethyl ether (1:1), yield of 70%, and m.p. = 188–190 °C, accompanied by decomposition and darkening. The product is slightly soluble in DMSO-d_6_; NMR spectra were taken in CDCl_3_, MW = 288.37 g/mol.

**^1^H-NMR** (600 MHz, 25 °C, CDCl_3_, *δ* ppm, *J* Hz): 2.07 (s, 3H, -CH_3_), 2.19 (s, 3H, -CH_3_), 3.77 (s, 3H, -OCH_3_), 3.86 (s, 3H, -OCH_3_), 5.23 (s, 1H, -CH), 6.21 (d, *J* = 1.8, 1H, -CH), 6.64 (d, *J* = 1.8, 1H, -CH), 8.04 (brs, 1H, -NH).

**^13^C{^1^H}-NMR** (150 MHz, 25 °C, CDCl_3_, δ ppm): 11.4 (2×CH3), 55.8 (2×OCH_3_), 96.4 (C-Ar), 100.4 (C-Ar), 110.8 (C-Ar), 127.4 (C-Ar), 128.4 (C-Ar), 129.6 (C-Ar), 137.0 (C-Ar), 154.1 (C-O), 156.8 (C-Ar), 164.5 (S-C=N), 170.0 (C-O).

**FTIR** (KBr, cm^−1^): ν (N-H)—3403; ν (Csp^2^-H)—2921, 2950; ν (C
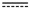
C)—1457, 1507, 1559; ν (C-O)—1151, 1202; ν (C-O-C)—1327; ν (C-N)—795, 1384; ν (C-S-C)—730.

**HRMS** *m*/*z* (ESI): calcd for C_15_H_15_N_2_O_2_S^-^ [M-H]^−^ 287.0860, found 287.0864.

Oxidative rearomatization of compound **4j** 2,2,2-trichloroethyl 2-(4- (benzyloxy)-1*H*-indol-3-yl)thiazole-3(2H)-carboxylate. The synthesized compound **5j** 2-(4-(benzyloxy)-1*H*-indol-3-yl)thiazole was isolated and purified by column chromatography on neutral aluminum oxide (Al_2_O_3_) with mixtures of petroleum/diethyl ether (1:1), yield of 82%, m.p. = 166–168 °C, and MW = 306.38 g/mol.

**^1^H-NMR** (400 MHz, 25 °C, DMSO-d_6_, *δ* ppm, *J* Hz): 5.30 (s, 2H, -OCH_2_Ph), 6.66 (dd, ^2^*J* = 5.7, ^3^*J* = 3.0, 1H, -CH), 7.02–7.08 (m, 2H, -2×CH), 7.26–7.38 (m, 3H, -3×CH), 7.42–7.52 (m, 3H, -CH), 7.72 (d, *J* = 3.3, 1H, -CH), 7.87 (s, 1H, -CH), 11.68 (s, 1H, -NH).

**^13^C{^1^H}-NMR** (100 MHz, 25 °C, DMSO-d_6_, *δ* ppm): 69.4 (CH_2_Ph), 102.5 (C-Ar), 106.0 (C-Ar), 111.1 (C-Ar), 114.8 (C-Ar), 119.2 (C-Ar), 123.3 (C-Ar), 126.4 (C-Ar), 128.1 (C-Ar), 128.2 (C-Ar), 128.7 (C-Ar), 137.5 (C-Ar), 138.8 (C-Ar), 142.3 (C-Ar), 152.5 (C-O), 163.2 (S-C=N).

**ATR-FTIR** (cm^−1^): ν (N-H)—3106; ν (Csp^2^-H)—2886, 2948; ν (C
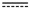
C)—1453, 1545, 1586; ν (C-O)—1070, 1119; ν (C-O-C)—1264, 1312; ν (C-N)—756, 852, 926; ν (C-S-C)—723.

**HRMS** *m*/*z* (ESI): calcd for C_18_H_13_N_2_OS^-^ [M-H]^−^ 305.0754, found 305.0759.

Oxidative rearomatization of compound **4k** 2,2,2-trichloroethyl 2-(4- (benzyloxy)-1*H*-indol-3-yl)-4,5-dimethylthiazole-3(2H)-carboxylate. The synthesized compound **5k** 2-(4-(benzyloxy)-1*H*-indol-3-yl)-4,5-dimethylthiazole was isolated by recrystallization with a mixture of petroleum/diethyl ether (1:1), yield of 90%, m.p. = 168–170 °C, and MW = 334.44 g/mol.

**^1^H-NMR** (400 MHz, 25 °C, DMSO-d_6_, *δ* ppm, *J* Hz): 2.20 (s, 3H, -CH_3_), 2.23 (s, 3H, -CH_3_), 5.25 (s, 2H, -OCH_2_Ph), 6.60–6.69 (m, 1H, -CH), 7.03 (s, 1H, -CH), 7.04 (d, *J* = 1.2, 1H, -CH), 7.29–7.39 (m, 3H, -3×CH), 7.47 (d, *J* = 6.5, 2H, -2×CH), 7.74 (brs, 1H), 11.58 (s, 1H, -NH).

**^13^C{^1^H}-NMR** (100 MHz, 25 °C, DMSO-d_6_, *δ* ppm): 11.1 (CH_3_), 14.9 (CH_3_), 69.6 (CH_2_Ph), 102.1 (C-Ar), 105.9 (C-Ar), 111.3 (C-Ar), 114.8 (C-Ar), 123.1 (C-Ar), 125.1 (C-Ar), 125.5 (C-Ar), 128.1 (C-Ar), 128.5 (C-Ar), 128.6 (C-Ar), 137.5 (C-Ar), 138.7 (C-Ar), 147.0 (C-Ar), 152.6 (C-O), 158.5 (S-C=N).

**ATR-FTIR** (cm^−1^): ν (N-H)—3101; ν (Csp^2^-H) –2917, 2942; ν (C
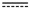
C)—1433, 1519, 1560; ν (C-O)—1070, 1119; ν (C-O-C)—1240, 1324; ν (C-N)—754, 781, 918; ν (C-S-C)—711.

**HRMS** *m*/*z* (ESI): calcd for C_20_H_17_N_2_OS^-^ [M-H]^−^ 333.1067, found 333.1069.

Oxidative rearomatization of compound **4l** 2,2,2-trichloroethyl 2-(5-hydroxy-1*H*-indol-3-yl)-4,5-dimethylthiazole-3(2H)-carboxylate. The synthesized compound **5l** 3-(4,5-dimethylthiazol-2-yl)-1*H*-indol-5-ol was isolated by recrystallization with a mixture of petroleum/diethyl ether (1:1), yield of 62%, m.p. = 233–235 °C, and MW = 244.31 g/mol.

**^1^H-NMR** (400 MHz, 25 °C, DMSO-d_6_, *δ* ppm, *J* Hz): 2.30 (s, 3H, -CH_3_), 2.35 (s, 3H, -CH_3_), 6.70 (dd, ^2^*J* = 8.7, ^3^*J* = 2.4, 1H, -CH), 7.25 (d, *J* = 8.6, 1H, -CH), 7.51 (d, *J* = 2.6, 1H, -CH), 7.80 (d, *J* = 2.8, 1H, -CH), 8.92 (s, 1H, -OH), 11.33 (s, 1H, -NH).

**^13^C{^1^H}-NMR** (100 MHz, 25 °C, DMSO-d_6_, *δ* ppm): 11.3 (CH_3_), 15.1 (CH_3_), 104.8 (C-Ar), 110.5 (C-Ar), 112.9 (C-Ar), 121.9 (C-Ar), 125.4 (C-Ar), 126.1 (C-Ar), 131.3 (C-Ar), 147.5 (C-Ar), 152.4 (C-O), 159.1 (S-C=N).

**ATR-FTIR** (cm^−1^): ν (O-H)—3251; ν (Csp^2^-H)—2921, 2851; ν (C
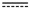
C)—1424, 1541, 1625; ν (C-O)—1078, 1123; ν (C-O-C)—1211, 1326; ν (C-N)—807; ν (C-S-C)—725.

**HRMS** *m*/*z* (ESI): calcd for C_13_H_11_N_2_OS^-^ [M-H]^−^ 243.0598, found 243.0595.

#### 4.1.4. In Silico QSAR Analysis

To predict selected properties using in silico methods, computational analyses were applied in this study using freely available software—T.E.S.T. (version 5.1.2). Table 4 presents data on the properties of newly synthesized oxy-camalexins. The T.E.S.T. software, developed by the U.S. Environmental Protection Agency (EPA, Washington, DC), was used to evaluate oral rat LD_50_, *Tetrahymena pyriformis* IGC_50_, *Daphnia magna* LC_50_, and water solubility at 25 °C [61], employing the nearest neighbour machine learning method. The toxicological profile and physicochemical properties of oxy-camalexins were assessed. Through the conducted in silico analysis, based on QSAR models and specialized software tools such as EPI Suite [71,75], VEGA [73,75], RDKit [69,70,76], PadelPy [69,70,76], and MolecularGraph.jl [74], key parameters were predicted, including the octanol/water partition coefficient (LogP) [66,67,68], topological polar surface area (TPSA) [83], and quantitative estimation of drug likeness (QED) [85], considering compliance with the Lipinski’s rule of five. The obtained results are presented in Table 5, Table 6, Table 7 and Table 8 and Tables S2 and S3 in the Supplementary Materials.

## 5. Conclusions

Modifying the camalexin moiety by introducing alkyl substituents in the thiazole ring, as well as varying the oxy groups in the indole ring, could significantly improve the physicochemical and biological properties of the new analogues. This strategy could expand the application possibilities of new camalexins in plant protection and the pharmaceutical industry.

This study presents an efficient two-step method for the synthesis of oxy-camalexins through the amidoalkylation of various substituted methoxy-, 4,6-dimethoxy-, 5-hydroxy-, and 4-benzyloxy-indoles with *N*-acylthiazolium reagents generated in situ*,* followed by oxidative rearomatization with *o*-chloranil. Eleven new *N*-acylated analogues of the natural 6-methoxycamalexin were synthesized with high yields (77–98%), and ten novel oxy-camalexins with yields ranging from 62 to 98%.

The synthetic strategy used is characterized by cost-effectiveness, a fast rate, accessible starting reagents, and scalability. Particularly significant is the successful synthesis of 2-(4,6-dimethoxy-1*H*-indol-3-yl)-4-methylthiazole and 2-(4,6-dimethoxy-1*H*-indol-3-yl)-4,5-dimethylthiazole, which are difficult to synthesize using classical methods. The proven fungicidal action of 5-methoxycamalexin motivated the synthesis of other 5-methoxy analogues on a gram-scale. Thus, the synthesis of 2,2,2-trichloroethyl 2-(5-methoxy-1*H*-indol-3-yl)-4,5-dimethylthiazole-3(2H)-carboxylate (**4e**) was successfully carried out on a gram-scale with high yield (96%), confirming the efficiency of the proposed method. Scaling up the process would ensure effective access to the necessary quantities of camalexins for studying a wide range of biological properties with applications in agrochemistry and medicine.

The conducted in silico analyses for predicting the ecotoxicological and physicochemical properties of oxy-camalexins show that, with few exceptions, the synthesized compounds meet the criteria of Lipinski’s rule of five and possess favourable drug potential, including high cellular permeability. The T.E.S.T software tool classified compounds **5b**, **5e**, **5h**, and **5i** with the lowest toxicity. These predictions highlight their prospective application as drug agents and bioactive molecules with potential use in the pharmaceutical and agrochemical fields. Despite the available literature data and the results obtained through in silico methods, final confirmation of activity requires additional in-depth analyses and studies. The examined compounds provide a good lead for their further biological testing. Therefore, our future scientific projects will focus on a thorough investigation of the biological activity of these new molecules and their potential mechanism of action.

## Figures and Tables

**Table 1:** Sources of natural indole phytoalexins.

Natural Phytoalexins	Isolated from
Camalexin	*Arabidopsis thaliana* and *Camelina sativa* (L.) [26]
5-hydroxycamalexin	Metabolite in *Rhizoctonia solani* [30]
6-methoxycamalexin	*Capsella bursa-pastoris* (L.) [26]
7-methoxycamalexin	*Neslia paniculata* (L.) *Desv.* [28]
6,7-dimethoxycamalexin	*Neslia paniculata* (L.) *Desv.* [28]

**Table 2:** The reaction conditions and yields of compounds **4a–l**, prepared according to Scheme 1.

Product 4a–l	R_1_	R_2_	R_3_	Reaction time, h	Yield, %	Melting Point, °C
**4a**	CH_3_	H	4-OCH_3_	30 min	98	175–177
**4b**	CH_3_	CH_3_	4-OCH_3_	20 min	97	191–193
**4c**	H	H	5-OCH_3_	40 min	93	162–164 [29] **
**4d**	CH_3_	H	5-OCH_3_	30 min	96	122–124
**4e**	CH_3_	CH_3_	5-OCH_3_	20 min	98 *	143–145
**4f**	H	H	6-OCH_3_	30 min	96	Oil
**4g**	CH_3_	CH_3_	6-OCH_3_	20 min	93	66–68
**4h**	CH_3_	H	4,6- (OCH_3_)_2_	10 min	92	171–173
**4i**	CH_3_	CH_3_	4,6- (OCH_3_)_2_	10 min	96	183–185
**4j**	H	H	4-BnO	30 min	97	54–56
**4k**	CH_3_	CH_3_	4-BnO	30 min	93	141–143
**4l**	CH_3_	CH_3_	5-OH	10 min	77	Oil

* Compound **4e** was obtained on a larger scale (1.255 g) in 96% yield; ** compound was also synthesized in cited work.

**Table 3:** Reaction conditions and yields of **5a–l**, obtained according to Scheme 2.

Product 5a–l	R_1_	R_2_	R_3_	Reaction Time, h	Yield, %	Melting Point, °C
**5a (p2)**	CH_3_	H	4-OCH_3_	30 min	86	180–181
**5b (p3)**	CH_3_	CH_3_	4-OCH_3_	30 min	80/93 *	212–214
**5c (p4)**	H	H	5-OCH_3_	15 min	98	112–114 [29,47] **
**5d (p5)**	CH_3_	H	5-OCH_3_	30 min	90	Oil
**5e (p6)**	CH_3_	CH_3_	5-OCH_3_	30 min	91	169–170
**5f (p7)**	H	H	6-OCH_3_	30 min	78	161–163 [47] **
**5g (p9)**	CH_3_	CH_3_	6-OCH_3_	20 min	91	163–165
**5h (p11)**	CH_3_	H	4,6- (OCH_3_)_2_	30 min	68	167–169
**5i (p12)**	CH_3_	CH_3_	4,6- (OCH_3_)_2_	30 min	70/80 *	188–190
**5j (p13)**	H	H	4-BnO	30 min	82	166–168
**5k (p15)**	CH_3_	CH_3_	4-BnO	30 min	90	168–170
**5l (p18)**	CH_3_	CH_3_	5-OH	40 min	62	233–235

* The reactions were carried out with DDQ in dichloromethane (CH_2_Cl_2_) for 10 min. at room temperature; ** compound was also synthesized in cited work.

**Table 4:** In silico toxicity prediction for oral rat LD_50_, *Tetrahymena pyriformis* IGC_50_, *Daphnia magna* LC_50_, and water solubility at 25 °C, applying *nearest neighbour* in the T.E.S.T software tool.

Compound	MW, g/mol	Oral Rat LD_50_ mg/kg	*T. pyriformis* IGC_50_ (48 h) mg/L	*Daphnia magna* LC_50_ (48 h) mg/L	Water Solubility at 25 °C mg/L
**p1**	230.29	631.90	27.29	2.25	25.02
**p2**	244.31	**2133.02**	7.60	1.71	11.34
**p3**	258.34	**3723.13**	8.03	1.81	11.99
**p4**	230.29	2010.54	27.29	2.25	22.13
**p5**	244.31	**2133.02**	7.60	1.71	11.34
**p6**	258.34	**3723.13**	8.03	11.71	11.99
**p7**	230.29	**2010.54**	27.29	2.25	22.13
**p8**	244.31	**2646.44**	7.60	1.71	61.85
**p9**	258.34	**2798.39**	8.03	11.71	11.99
**p10**	260.31	**2819.73**	8.09	0.84	65.90
**p11**	274.34	**3550.89**	8.53	12.44	18.98
**p12**	288.37	**3999.40**	8.97	13.07	19.95
**p13**	306.38	**2552.52**	8.10	2.15	21.20
**p14**	320.41	1519.66	6.53	2.24	22.17
**p15**	334.44	524.03	6.81	15.16	23.14
**p16**	216.26	1657.10	25.63	2.11	38.28
**p17**	230.29	1764.60	27.29	4.47	58.30
**p18**	244.31	**2133.02**	7.60	0.78	61.85

The predicted lower toxicity > 2000 mg/kg is indicated in bold.

**Table 5:** Computational predictions of LogP, using different models.

Compound	Wildman & Crippen (RDkit)	Wildman & Crippen (PadelPy)	Wildman & Crippen Julia	EPI Suite	VEGA
**p1**	2.79	3.31	3.3	2.9	2.9
**p2**	3.09	3.8	3.6	3.45	3.45
**p3**	3.4	3.34	3.91	4	4
**p4**	2.79	3.31	3.3	2.9	2.9
**p5**	3.09	3.8	3.6	3.45	3.45
**p6**	3.4	3.34	3.91	4	4
**p7**	2.79	3.31	3.3	2.9	2.9
**p8**	3.09	3.8	3.6	3.45	3.45
**p9**	3.4	3.34	3.91	4	4
**p10**	2.79	3.32	3.3	3	3
**p11**	3.1	3.81	3.61	3.53	3.53
**p12**	3.41	3.35	3.92	4.1	4.08
**p13**	4.36	4.85	4.87	4.6	4.61
**p14**	4.87	4.11	4.87	4.9	4.54
**p15**	5.18	4.13	5.18	5.45	5.1
**p16**	2.48	2.88	3	2.34	2.34
**p17**	2.79	3.37	3.3	2.9	2.9
**p18**	3.1	2.91	3.61	3.43	3.43

**Table 6:** DT_50_ (half-life in days—EPI Suite).

Compound	Air	Water	Soil	Sediments
**p1**	0.05	37.5	75	337.5
**p2**	0.05	37.5	75	337.5
**p3**	0.05	37.5	75	337.5
**p4**	0.05	37.5	75	337.5
**p5**	0.05	37.5	75	337.5
**p6**	0.05	37.5	75	337.5
**p7**	0.05	37.5	75	337.5
**p8**	0.05	37.5	75	337.5
**p9**	0.05	37.5	75	337.5
**p10**	0.05	37.5	75	337.5
**p11**	0.05	37.5	75	337.5
**p12**	0.05	37.5	75	337.5
**p13**	0.05	37.5	75	337.5
**p14**	0.3	37.5	75	337.5
**p15**	0.33	60	120	541.6
**p16**	0.05	15	30	135
**p17**	0.05	37.5	75	337.5
**p18**	0.05	37.5	75	337.5

**Table 7:** Molecular descriptors (Python language, RDKit library).

Compound	SA Score	NP Score	SPS	BertzCT	AvgIpc	BalabanJ
**p1**	3.127	−0.6	12.93	527.17	2.95	2.16
**p2**	3.047	−0.76	12.94	581.83	2.93	2.13
**p3**	3.084	−0.67	12.94	603.98	2.93	2.13
**p4**	3.126	−0.73	12.93	527.17	2.95	2.09
**p5**	3.046	−0.89	12.94	581.83	2.93	2.07
**p6**	3.083	−0.79	12.94	603.98	2.92	2.07
**p7**	3.082	−0.59	12.93	527.17	2.95	2.04
**p8**	3.004	−0.76	12.94	581.83	2.95	2.03
**p9**	3.043	−0.67	12.94	603.98	2.92	2.03
**p10**	3.157	−0.35	12.66	582.75	3.04	2.13
**p11**	3.089	−0.5	12.68	638.05	3.02	2.12
**p12**	3.126	−0.44	12.7	660.8	3.01	2.12
**p13**	2.749	−0.74	12.45	789.31	3.22	1.64
**p14**	3.079	−0.59	15.6	833.48	3.19	1.63
**p15**	3.139	−0.56	15.5	858.15	3.17	1.65
**p16**	3.368	−0.24	13.2	512.97	2.82	2.13
**p17**	3.269	−0.43	13.18	567.47	2.83	2.1
**p18**	3.296	−0.36	13.7	589.46	2.82	2.11

**Table 8:** Molecular descriptors (Python language, RDKit library) *.

Compound	QED	Fraction Csp^3^	Labute ASA	Mol MR	Max Estate Index	Min Estate Index	TPSA
**p1**	0.79	0.08	97.41	63.98	5.36	0.84	36.22
**p2**	0.81	0.15	103.77	68.71	5.4	0.84	36.22
**p3**	0.82	0.21	110.14	73.45	5.42	0.85	36.22
**p4**	0.79	0.08	97.41	63.98	5.21	0.84	36.22
**p5**	0.81	0.15	103.77	68.71	5.24	0.84	36.22
**p6**	0.82	0.21	110.14	73.45	5.26	0.84	36.22
**p7**	0.79	0.08	97.41	63.98	5.16	0.82	36.22
**p8**	0.81	0.15	103.77	68.71	5.18	0.83	36.22
**p9**	0.82	0.21	110.14	73.45	5.2	0.83	36.22
**p10**	0.85	0.15	108.88	70.53	5.42	0.73	45.45
**p11**	0.96	0.21	115.25	75.27	5.45	0.74	45.45
**p12**	0.96	0.26	121.61	80	5.48	0.74	45.45
**p13**	0.71	0.05	132.46	88.2	6.03	0.54	36.22
**p14**	0.68	0.15	138.86	94.06	6.09	0.093	34.48
**p15**	0.65	0.2	145.23	98.8	6.12	0.087	34.48
**p16**	0.79	0	90.72	59.09	9.42	0.25	47.22
**p17**	0.81	0.08	97.09	63.83	9.48	0.25	47.22
**p18**	0.83	0.15	103.45	68.56	9.53	0.26	47.22

* Continuation of the results presented in Table 7.

## Data Availability

The original contributions presented in this study are included in the article/Supplementary Materials. Further inquiries can be directed to the corresponding author(s).

## Supplementary Materials

The following supporting information can be downloaded at https://www.mdpi.com/article/10.3390/molecules30092049/s1: Figures S1–S96. PDF—processed ^1^H-, ^13^C{^1^H)-, HSQC-NMR, FTIR and ESI-HRMS spectra; Tables S1–S3; Scheme S1—Proposed mechanism of oxidative rearomatization with DDQ.

## References

[1] Jeandet P., Delaunois B., Aziz A., Donnez D., Vasserot Y., Cordelier S., Courot E. (2012). Metabolic Engineering of Yeast and Plants for the Production of the Biologically Active Hydroxystilbene, Resveratrol. J. Biomed. Biotechnol..

[2] Hasegawa M., Mitsuhara I., Seo S., Okada K., Yamane H., Iwai T., Ohashi Y. (2014). Analysis on Blast Fungus-Responsive Characters of a Flavonoid Phytoalexin Sakuranetin; Accumulation in Infected Rice Leaves, Antifungal Activity and Detoxification by Fungus. Molecules.

[3] Langcake P., Pryce R.J. (1976). The production of resveratrol by Vitis vinifera and other members of the Vitaceae as a response to infection or injury. Physiol. Plant Pathol..

[4] Koga J., Shimura M., Oshima K., Ogawa N., Yamauchi T., Ogasawara N. (1995). Phytocassanes A, B, C and D, Novel Diterpene Phytoalexins from Rice, Oryza Sativa, L. Tetrahedron.

[5] Großkinsky D.K., van der Graaff E., Roitsch T. (2012). Phytoalexin transgenics in crop protection-Fairy tale with a happy end?. Plant Sci..

[6] Jeandet P., Hébrard C., Deville M.-A., Cordelier S., Dorey S., Aziz A., Crouzet J. (2014). Deciphering the Role of Phytoalexins in Plant-Microorganism Interactions and Human Health. Molecules.

[7] Ahuja I., Kissen R., Bones A.M. (2012). Phytoalexins in Defense against Pathogens. Trends Plant Sci..

[8] Jeandet P. (2018). Structure, Chemical Analysis, Biosynthesis, Metabolism, Molecular Engineering, and Biological Functions of Phytoalexins. Molecules.

[9] Pedras M.S.C., Ahiahonu P.W.K. (2005). Metabolism and Detoxification of Phytoalexins and Analogs by Phytopathogenic Fungi. Phytochemistry.

[10] Nguyen N.H., Trotel-Aziz P., Clément C., Jeandet P., Baillieul F., Aziz A. (2022). Camalexin accumulation as a component of plant immunity during interactions with pathogens and beneficial microbes. Planta.

[11] Joubert A., Bataille-Simoneau N., Campion C., Guillemette T., Hudhomme P., Iacomi-Vasilescu B., Leroy T., Pochon S., Poupard P., Simoneau P. (2011). Cell Wall Integrity and High Osmolarity Glycerol Pathways Are Required for Adaptation of Alternaria Brassicicola to Cell Wall Stress Caused by Brassicaceous Indolic Phytoalexins. Cell. Microbiol..

[12] Gonçalves A.P., Heller J., Daskalov A., Videira A., Glass N.L. (2017). Regulated Forms of Cell Death in Fungi. Front. Microbiol..

[13] Glawischnig E. (2007). Camalexin. Phytochemistry.

[14] Pedras M.S.C., Yaya E.E., Glawischnig E. (2011). The Phytoalexins from Cultivated and Wild Crucifers: Chemistry and Biology. Nat. Prod. Rep..

[15] Nguyen N.H., Trotel-Aziz P., Villaume S., Rabenoelina F., Clément C., Baillieul F., Aziz A. (2022). Priming of Camalexin Accumulation in Induced Systemic Resistance by Beneficial Bacteria against Botrytis Cinerea and Pseudomonas Syringae Pv. Tomato DC3000. J. Exp. Bot..

[16] Wilson S.K., Pretorius T., Naidoo S. (2023). Mechanisms of Systemic Resistance to Pathogen Infection in Plants and Their Potential Application in Forestry. BMC Plant Biol..

[17] Choudhary D.K., Prakash A., Johri B.N. (2007). Induced Systemic Resistance (ISR) in Plants: Mechanism of Action. Indian J. Microbiol..

[18] Zhu Y., Zhao J., Luo L., Gao Y., Bao H., Li P., Zhang H. (2021). Research Progress of Indole Compounds with Potential Antidiabetic Activity. Eur. J. Med. Chem..

[19] Wan Y., Li Y., Yan C., Yan M., Tang Z. (2019). Indole: A Privileged Scaffold for the Design of Anti-Cancer Agents. Eur. J. Med. Chem..

[20] Umer S.M., Solangi M., Khan K.M., Saleem R.S.Z. (2022). Indole-Containing Natural Products 2019–2022: Isolations, Reappraisals, Syntheses, and Biological Activities. Molecules.

[21] Jeandet P., Clément C., Courot E., Cordelier S. (2013). Modulation of phytoalexin biosynthesis in engineered plants for disease resistance. Int. J. Mol. Sci..

[22] Müller K.O., Börger H. (1940). Experimentelle untersuchungen über die *Phytophthora* resistenz der *Kartoffel*. Arbeit. Biol. Reichsant Land Forstwirtsch.

[23] Jeandet P. (2015). Phytoalexins: Current Progress and Future Prospects. Molecules.

[24] Jimenez L.D., Ayer W.A., Tewari J.P. (1997). Phytoalexins Produced in the Leaves of Capsella Bursa-Pastoris (Shepherd’s Purse). Phytoprotection.

[25] Pedras M.S.C., Zheng Q.A., Sarma-Mamillapalle V.K. (2007). The phytoalexins from Brassicaceae: Structure, biological activity, synthesis and biosynthesis. Nat. Prod. Commun..

[26] Browne L.M., Conn K.L., Ayert W.A., Tewari J.P. (1991). The Camalexins: New Phytoalexins Produced in the Leaves of Camelina Sativa (Cruciferae). Tetrahedron.

[27] Pedras M.S.C., Khan A.Q. (2000). Biotransformation of the Phytoalexin Camalexin by the Phytopathogen Rhizoctonia Solani. Phytochemistry.

[28] Pedras M.S.C., Alavi M., Abdoli A. (2021). Phytoalexins and Signalling Metabolites Produced in the Wild Crucifer Neslia Paniculata: Camalexins and Arabidopsides. ChemRxiv.

[29] Liao A., Li L., Wang T., Lu A., Wang Z., Wang Q. (2022). Discovery of Phytoalexin Camalexin and Its Derivatives as Novel Antiviral and Antiphytopathogenic-Fungus Agents. J. Agric. Food Chem..

[30] Pedras M.S.C., Khan A.Q. (1997). Unprecedented Detoxification of the Phytoalexin Camalexin by a Root Rot Pathogen. Bioorg. Med. Chem. Lett..

[31] Pedras M.S.C., Minic Z., Sarma-Mamillapalle V.K. (2009). Synthetic Inhibitors of the Fungal Detoxifying Enzyme Brassinin Oxidase Based on the Phytoalexin Camalexin Scaffold. J. Agric. Food Chem..

[32] Pedras M.S.C., Abdoli A. (2017). Pathogen Inactivation of Cruciferous Phytoalexins: Detoxification Reactions, Enzymes and Inhibitors. RSC Adv..

[33] Pedras M.S.C., Abdoli A., Sarma-Mamillapalle V.K. (2017). Inhibitors of the Detoxifying Enzyme of the Phytoalexin Brassinin Based on Quinoline and Isoquinoline Scaffolds. Molecules.

[34] Manasa K., Chitra V. (2020). Evaluation of In-vitro Antioxidant Activity of Camalexin—A Novel Anti-Parkinson’s agent. Res. J. Pharm. Tech..

[35] Egbuonu A.C.C., Eneogwe J. (2018). Phytoalexins: Current and Possible Future Applications in Human Health and Diseases Control. Int. J. Mol. Biol..

[36] Zigová M., Michalková R., Mojžiš J. (2024). Anticancer Potential of Indole Phytoalexins and Their Analogues. Molecules.

[37] Pilatova M., Ivanova L., Kutschy P., Varinska L., Saxunova L., Repovska M., Sarissky M., Seliga R., Mirossay L., Mojzis J. (2013). In vitro toxicity of camalexin derivatives in human cancer and non-cancer cells. Toxicol. Vitro.

[38] Smith B., Randle D., Mezencev R., Thomas L.S., Hinton C., Odero-Marah V. (2014). Camalexin-Induced Apoptosis in Prostate Cancer Cells Involves Alterations of Expression and Activity of Lysosomal Protease Cathepsin D. Molecules.

[39] Chripkova M., Drutovic D., Pilatova M., Mikes J., Budovska M., Vaskova J., Broggini M., Mirossay L., Mojzis J. (2014). Brassinin and its derivatives as potential anticancer agents. Toxicol. Vitr..

[40] Yang Y., Wang G., Wu W., Yao S., Han X., He D., He J., Zheng G., Zhao Y., Cai Z. (2018). Camalexin Induces Apoptosis via the ROS-ER Stress-Mitochondrial Apoptosis Pathway in AML Cells. Oxid. Med. Cell. Longev..

[41] Zigová M., Miškufová V., Budovská M., Michalková R., Mojžiš J. (2024). Exploring the Antiproliferative and Modulatory Effects of 1-Methoxyisobrassinin on Ovarian Cancer Cells: Insights into Cell Cycle Regulation, Apoptosis, Autophagy, and Its Interactions with NAC. Molecules.

[42] Chripkova M., Zigo F., Mojzis J. (2016). Antiproliferative Effect of Indole Phytoalexins. Molecules.

[43] Mezencev R., Galizzi M., Kutschy P., Docampo R. (2009). Trypanosoma cruzi: Antiproliferative effect of indole phytoalexins on intracellular amastigotes in vitro. Exp. Parasitol..

[44] Moody C.J., Roffey J.R., Stephens M.A. (1997). Stratford IJ. Synthesis and cytotoxic activity of indolyl thiazoles. Anticancer Drugs..

[45] Smith B.A., Neal C.L., Chetram M., Vo B., Mezencev R., Hinton C., Odero-Marah V.A. (2013). The phytoalexin camalexin mediates cytotoxicity towards aggressive prostate cancer cells via reactive oxygen species. J. Nat. Med..

[46] Rogers E.E., Glazebrook J., Ausubel F.M. (1996). Mode of Action of the Arabidopsis Thaliana Phytoalexin Camalexin and Its Role in Arabidopsis-Pathogen Interactions. Mol. Plant-Microbe Interact..

[47] Ayer W.A., Craw P.A., Ma Y., Miao S. (1992). Synthesis of camalexin and related phytoalexins. Tetrahedron.

[48] Dzurilla M., Kutschy P., Zaletova J., Ruzinsky M., Kovacik V. (2001). Synthesis of Camalexin. Molecules.

[49] Tasch B.O.A., Antovic D., Merkul E., Müller T.J.J. (2013). One-Pot Synthesis of Camalexins and 3,3′-Biindoles by the Masuda Borylation-Suzuki Arylation (MBSA) Sequence. Eur. J. Org. Chem..

[50] Pedras M.S.C., Abdoli A. (2018). Methoxycamalexins and Related Compounds: Syntheses, Antifungal Activity and Inhibition of Brassinin Oxidase. Bioorganic Med. Chem..

[51] Stremski Y., Statkova-Abeghe S., Angelov P., Ivanov I. (2018). Synthesis of Camalexin and Related Analogues. J. Heterocycl. Chem..

[52] Stremski Y., Ahmedova A., Dołega A., Statkova-Abeghe S., Kirkova D. (2021). Oxidation Step in the Preparation of Benzocamalexin: The Crystallographic Evidence. Mendeleev Commun..

[53] Tantak M.P., Wang J., Singh R.P., Kumar A., Shah K., Kumar D. (2015). 2-(3′-Indolyl)-*N*-Arylthiazole-4-Carboxamides: Synthesis and Evaluation of Antibacterial and Anticancer Activities. Bioorg. Med. Chem. Lett..

[54] Kong M., Liang J., Ali Q., Wen W., Wu H., Gao X., Gu Q. (2021). 5-Methoxyindole, a Chemical Homolog of Melatonin, Adversely Affects the Phytopathogenic Fungus Fusarium Graminearum. Int. J. Mol. Sci..

[55] Gorunova O.N. (2021). Modification of Heterocycles by Amidoalkylation. Ineos Open.

[56] Mazurkiewicz R., Październiok-Holewa A., Adamek J., Zielińska K. (2014). α-Amidoalkylating agents: Structure, synthesis, reactivity and application. Adv. Heterocycl. Chem..

[57] Raies A.B., Bajic V.B. (2016). In Silico Toxicology: Computational Methods for the Prediction of Chemical Toxicity. Wiley Interdiscip. Rev. Comput. Mol. Sci..

[58] Garralaga M.P., Lomba L., Zuriaga E., Santander S., Giner B. (2022). Key Properties for the Toxicity Classification of Chemicals: A Comparison of the REACH Regulation and Scientific Studies Trends. Appl. Sci..

[59] Thamaraikani T., Karnam M., Velapandian C. (2022). In Silico Docking of Novel Phytoalkaloid Camalexin in the Management of Benomyl Induced Parkinson’s Disease and its In Vivo Evaluation by Zebrafish Model. CNS Neurol. Disord. Drug Targets.

[60] Mounika K., Sumedha J., Raveena G., Shivani K., Neelima K., Shireesha S.M., Anuradha Bai S. (2022). In silico Molecular Properties Predictions of Novel Camalexin Derivatives. Int. J. All Res. Educ. Sci. Methods.

[61] Martin T.M. (2020). User’s Guide for T.E.S.T. (Toxicity Estimation Software Tool), & Todd. User’s Guide for T. E. S. T. (Toxicity Estimation Software Tool) Version 5.1 A Java Application to Estimate Toxicities and Physical Properties from Molecular Structure. https://www.epa.gov/chemical-research/toxicity-estimation-software-tool-test..

[62] Guilhermino L., Diamantino T., Carolina Silva M., Soares A.M.V.M. (2000). Acute Toxicity Test with Daphnia Magna: An Alternative to Mammals in the Prescreening of Chemical Toxicity?. Ecotoxicol. Environ. Saf..

[63] United States Environmental Protection Agency (2002). Methods for Measuring the Acute Toxicity of Effluents and Receiving Waters to Freshwater and Marine Organisms.

[64] UE ANNEX to the Commission Regulation Amending, for the Purpose of Its Adaptation to Technical Progress, the Annex to Regulation (EC) No 440/2008 Laying Down Test Methods Persuant to Regulation (EC) No 1907/2006. REACH 2023..

[65] OECD Guidelines for the Testing of Chemicals, Section 2 (2004). Test No. 202: Daphnia sp. Acute Immobilisation Test.

[66] Lipinski C.A. (2000). Drug-like Properties and the Causes of Poor Solubility and Poor Permeability. J. Pharmacol. Toxicol. Methods.

[67] Pollastri M.P. (2010). Overview on the Rule of Five. Curr. Protoc. Pharmacol..

[68] Varma M.V., Perumal O.P., Panchagnula R. (2006). Functional Role of P-Glycoprotein in Limiting Peroral Drug Absorption: Optimizing Drug Delivery. Curr. Opin. Chem. Biol..

[69] Landrum G. (2013). Rdkit documentation. Release.

[70] Jana T., Sarkar D., Ganguli D., Mukherjee S.K., Mandal R.S., Das S. (2024). ABDpred: Prediction of active antimicrobial compounds using supervised machine learning techniques. J. Med. Res..

[71] (2007). US Environmental Protection Agency, Science Advisory Board Review of the Estimation Programs Interface Suite (EPI Suite™), Document EPA-SAB-07-11, US EPA, Washington, DC, USA. https://www.epa.gov/tsca-screening-tools/epi-suitetm-estimation-program-interface.

[72] Card M.L., Gomez-Alvarez V., Lee W.H., Lynch D.G., Orentas N.S., Lee M.T., Wong E.M., Boethling R.S. (2017). History of EPI Suite^TM^ and Future Perspectives on Chemical Property Estimation in US Toxic Substances Control Act New Chemical Risk Assessments. Environ. Sci. Process. Impacts.

[73] Benfenati E., Manganaro A., Gini G. (2013). VEGA-QSAR: AI inside a Platform for Predictive Toxicology. CEUR Workshop Proc..

[74] Liyaqat T., Ahmad T., Saxena C. (2024). Advancements in Molecular Property Prediction: A Survey of Single and Multimodal Approaches. arXiv.

[75] Vega-Garcia P., Lok C.S.C., Marhoon A., Schwerd R., Johann S., Helmreich B. (2022). Modelling the environmental fate and behavior of biocides used in façades covered with mortars and plasters and their transformation products. Build. Environ..

[76] Aires-de-Sousa J. (2024). GUIDEMOL: A Python graphical user interface for molecular descriptors based on RDKit. Mol. Inform..

[77] Ertl P., Schuffenhauer A. (2009). Estimation of Synthetic Accessibility Score of Drug-like Molecules Based on Molecular Complexity and Fragment Contributions. J. Cheminform..

[78] Ertl P., Roggo S., Schuffenhauer A. (2008). Natural Product-Likeness Score and Its Application for Prioritization of Compound Libraries. J. Chem. Inf. Model..

[79] Durham E., Dorr B., Woetzel N., Staritzbichler R., Meiler J. (2009). Solvent Accessible Surface Area Approximations for Rapid and Accurate Protein Structure Prediction. J. Mol. Model..

[80] Gandhi H.A., White A.D. (2022). Explaining Structure—Activity Relationships Using Locally Faithful Surrogate Models. Theor. Comput. Chem..

[81] Ye X., Cui N., Ou W., Liu D., Bao Y., Ai B., Zhou Y. (2024). Explainable optimized 3D-MoRSE descriptors for the power conversion efficiency prediction of molecular passivated perovskite solar cells through machine learning. J. Mater. Chem. A.

[82] Estrada E., Gutierrez Y. (2001). The Balaban J index in the multidimensional space of generalized topological indices. Generalizations and QSPR improvements. Match Commun. Math. Comput. Chem..

[83] Prasanna S., Doerksen R. (2008). Topological Polar Surface Area: A Useful Descriptor in 2D-QSAR. Curr. Med. Chem..

[84] Le Fèvre R.J.W. (1965). Molecular Refractivity and Polarizability. Adv. Phys. Org. Chem..

[85] Tian S., Wang J., Li Y., Li D., Xu L., Hou T. (2015). The Application of in Silico Drug-Likeness Predictions in Pharmaceutical Research. Adv. Drug Deliv. Rev..

