# Supplementary material for: An Efficient Method for the Synthesis and In Silico Study of Novel Oxy-Camalexins
Source: Molecules. 2025 May 4;30(9):2049. doi: 10.3390/molecules30092049 (PMC12073450; doi:10.3390/molecules30092049)
Supplement: Supplementary file 1 [file molecules-30-02049-s001.zip › molecules-3590159-supplementary.pdf]

Supplementary Materials
An efficient method for the synthesis and *in silico* study of novel oxy-camalexins

Maria Bachvarova[1], Yordan Stremski[1,*], Donyo Ganchev[2], Stela Statkova-Abeghe[1], Plamen Angelov[1] and Iliyan Ivanov[1]

[1] Department of Organic Chemistry, University of Plovdiv "Paisii Hilendarski", 24 Tsar Asen Str., 4000 Plovdiv, Bulgaria

[2] Department of General Chemistry, Agricultural University of Plovdiv, 12 Mendeleev Blvd, 4000 Plovdiv, Bulgaria

* Correspondence: stremski@uni-plovdiv.net; Tel.: +359-32-261-346

Table of Contents:

Spectral data of *N*-acylated oxy-camalexin analogues:

Figure S1 ^{1}H-NMR spectrum **4a**, **page 3**
Figure S2 ^{13}C{^{1}H}-NMR spectrum **4a**, **page 3**
Figure S3 FTIR spectrum **4a**, **page 4**
Figure S4 ESI-HRMS spectrum **4a**, **page 4**
Figure S5 ^{1}H-NMR spectrum **4b**, **page 5**
Figure S6 ^{13}C{^{1}H}-NMR spectrum **4b**, **page 5**
Figure S7 FTIR spectrum **4b**, **page 6**
Figure S8 ESI-HRMS spectrum **4b**, **page 6**
Figure S9 ^{1}H-NMR spectrum **4c**, **page 7**
Figure S10 ^{13}C{^{1}H}-NMR spectrum **4c**, **page 7**
Figure S11 HSQC-NMR spectrum **4c**, **page 8**
Figure S12 ATR-FTIR spectrum **4c**, **page 8**
Figure S13 ESI-HRMS spectrum **4c**, **page 9**
Figure S14 ^{1}H-NMR spectrum **4d**, **page 9**
Figure S15 ^{13}C{^{1}H}-NMR spectrum **4d**, **page 10**
Figure S16 FTIR spectrum **4d**, **page 10**
Figure S17 ESI-HRMS spectrum **4d**, **page 11**
Figure S18 ^{1}H-NMR spectrum **4e**, **page 11**
Figure S19 ^{13}C{^{1}H}-NMR spectrum **4e**, **page 12**
Figure S20 FTIR spectrum **4e**, **page 12**
Figure S21 ESI-HRMS spectrum **4e**, **page 13**
Figure S22 ^{1}H-NMR spectrum **4f**, **page 13**
Figure S23 ^{13}C{^{1}H}-NMR spectrum **4f**, **page 14**
Figure S24 ATR-FTIR spectrum **4f**, **page 14**
Figure S25 ESI-HRMS spectrum **4f**, **page 15**
Figure S26 ^{1}H-NMR spectrum **4g**, **page 15**
Figure S27 ^{13}C{^{1}H}-NMR spectrum **4g**, **page 16**
Figure S28 ATR-FTIR spectrum **4g**, **page 16**
Figure S29 ESI-HRMS spectrum **4g**, **page 17**
Figure S30 ^{1}H-NMR spectrum **4h**, **page 17**
Figure S31 ^{13}C{^{1}H}-NMR spectrum **4h**, **page 18**
Figure S32 FTIR spectrum **4h**, **page 18**
Figure S33 ESI-HRMS spectrum **4h**, **page 19**
Figure S34, S35 ^{1}H-NMR spectra **4i**, **page 19, 20**
Figure S36 ^{13}C{^{1}H}-NMR spectrum **4i**, **page 20**
Figure S37 FTIR spectrum **4i**, **page 21**
Figure S38 ESI-HRMS spectrum **4i**, **page 21**
Figure S39 ^{1}H-NMR spectrum **4j**, **page 22**
Figure S40 ^{13}C{^{1}H}-NMR spectrum **4j**, **page 22**
Figure S41 ATR-FTIR spectrum **4j**, **page 23**
Figure S42 ESI-HRMS spectrum **4j**, **page 23**
Figure S43 ^{1}H-NMR spectrum **4k**, **page 24**
Figure S44 ^{13}C{^{1}H}-NMR spectrum **4k**, **page 24**
Figure S45 ATR-FTIR spectrum **4k**, **page 25**
Figure S46 ESI-HRMS spectrum **4k**, **page 25**
Figure S47 ^{1}H-NMR spectrum **4l**, **page 26**
Figure S48 ^{13}C{^{1}H}-NMR spectrum **4l**, **page 26**
Figure S49 FTIR spectrum **4l**, **page 27**
Figure S50 ESI-HRMS spectrum **4l**, **page 27**

Spectral data of oxy-camalexins:

Figure S51 ^{1}H-NMR spectrum **5a**, **page 28**
Figure S52 ^{13}C{^{1}H}-NMR spectrum **5a**, **page 28**
Figure S53 FTIR spectrum **5a**, **page 29**
Figure S54 ESI-HRMS spectrum **5a**, **page 29**
Figure S55 ^{1}H-NMR spectrum **5b**, **page 30**
Figure S56 ^{13}C{^{1}H}-NMR spectrum **5b**, **page 30**
Figure S57 FTIR spectrum **5b**, **page 31**
Figure S58 ESI-HRMS spectrum **5b**, **page 31**
Figure S59 ^{1}H-NMR spectrum **5c**, **page 32**
Figure S60 ^{13}C{^{1}H}-NMR spectrum **5c**, **page 32**
Figure S61 ATR-FTIR spectrum **5c**, **page 33**
Figure S62 ESI-HRMS spectrum **5c**, **page 33**
Figure S63 ^{1}H-NMR spectrum **5d**, **page 34**
Figure S64 ^{13}C{^{1}H}-NMR spectrum **5d**, **page 34**
Figure S65 ESI-HRMS spectrum **5d**, **page 35**
Figure S66 ^{1}H-NMR spectrum **5e**, **page 35**
Figure S67 ^{13}C{^{1}H}-NMR spectrum **5e**, **page 36**
Figure S68 FTIR spectrum **5e**, **page 36**
Figure S69 ESI-HRMS spectrum **5e**, **page 37**
Figure S70 ^{1}H-NMR spectrum **5f**, **page 37**
Figure S71 ^{13}C{^{1}H}-NMR spectrum **5f**, **page 38**
Figure S72 ATR-FTIR spectrum **5f**, **page 38**
Figure S73 ESI-HRMS spectrum **5f**, **page 39**
Figure S74 ^{1}H-NMR spectrum **5g**, **page 39**
Figure S75 ^{13}C{^{1}H}-NMR spectrum **5g**, **page 40**
Figure S76 ATR-FTIR spectrum **5g**, **page 40**
Figure S77 ESI-HRMS spectrum **5g**, **page 41**
Figure S78 ^{1}H-NMR spectrum **5h**, **page 41**
Figure S79 ^{13}C{^{1}H}-NMR spectrum **5h**, **page 42**
Figure S80 FTIR spectrum **5h**, **page 42**
Figure S81 ESI-HRMS spectrum **5h**, **page 43**
Figure S82 ^{1}H-NMR spectra **5i**, **page 43**
Figure S83 ^{13}C{^{1}H}-NMR spectrum **5i**, **page 44**
Figure S84 ESI-HRMS spectrum **5i**, **page 44**
Figure S85 ^{1}H-NMR spectrum **5j**, **page 45**
Figure S86 ^{13}C{^{1}H}-NMR spectrum **5j**, **page 45**
Figure S87 ATR-FTIR spectrum **5j**, **page 46**
Figure S88 ESI-HRMS spectrum **5j**, **page 46**
Figure S89 ^{1}H-NMR spectrum **5k**, **page 47**
Figure S90 ^{13}C{^{1}H}-NMR spectrum **5k**, **page 47**
Figure S91 ATR-FTIR spectrum **5k**, **page 48**
Figure S92 ESI-HRMS spectrum **5k**, **page 48**
Figure S93 ^{1}H-NMR spectrum **5l**, **page 49**
Figure S94 ^{13}C{^{1}H}-NMR spectrum **5l**, **page 49**
Figure S95 ATR-FTIR spectrum **5l**, **page 50**
Figure S96 ESI-HRMS spectrum **5l**, **page 50**
Table S1 Structures and abbreviations of not synthesized compounds, **page 51**
Table S2 Lipinski Rule of Five, **page 52**
Table S3 Atomic hybridization assessment, **page 52**
Scheme S1 Proposed mechanism of oxidative rearomatization with DDQ, **page 53**

Spectral data of *N*-acylated oxy-camalexin analogues:

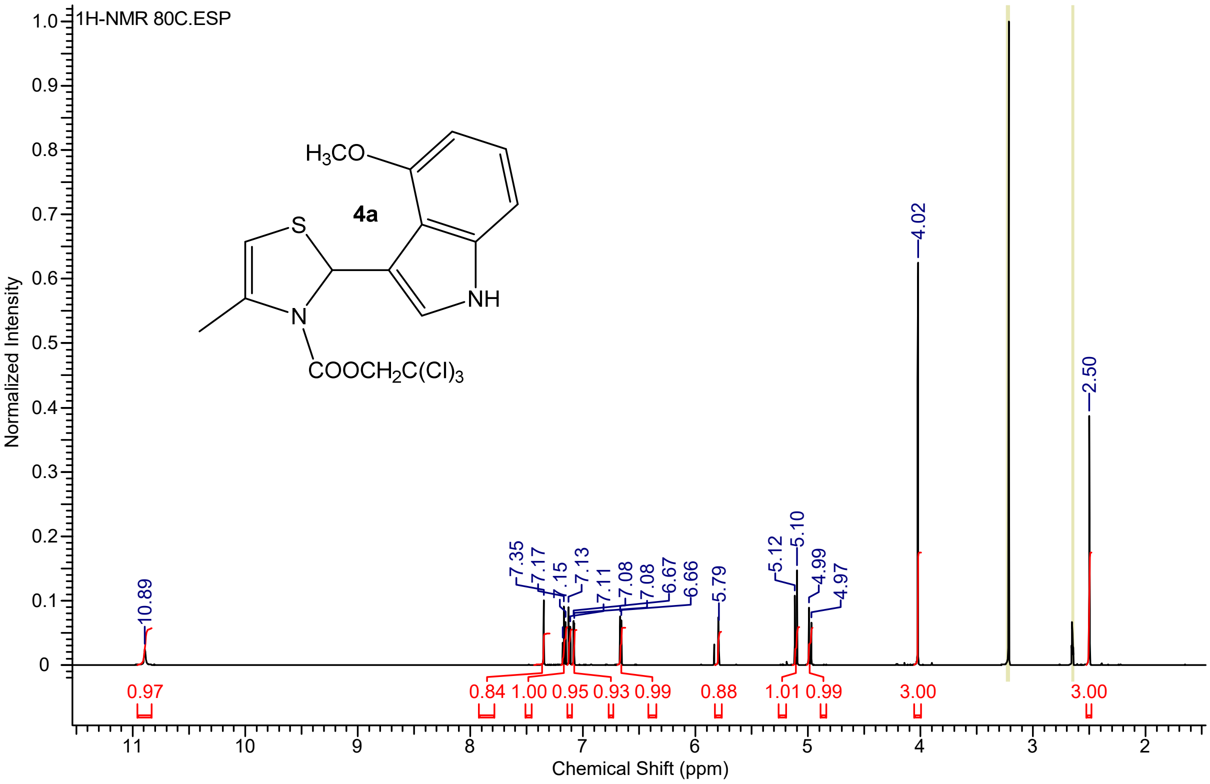

Figure S1 ^{1}H-NMR spectrum of **4a** - DMSO-d$_6$, 80 °C, 600 MHz

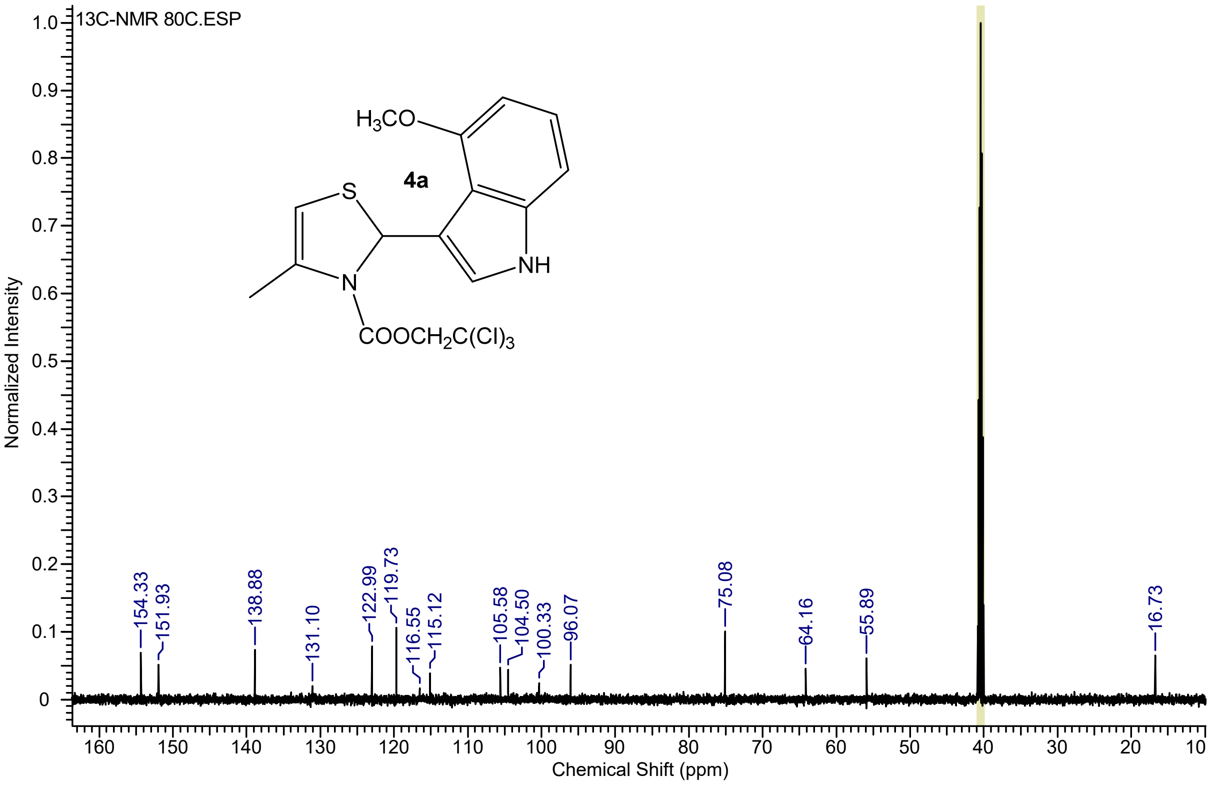

Figure S2 ^{13}C{^{1}H}-NMR spectrum of **4a** - DMSO-d$_6$, 80 °C, 150 MHz

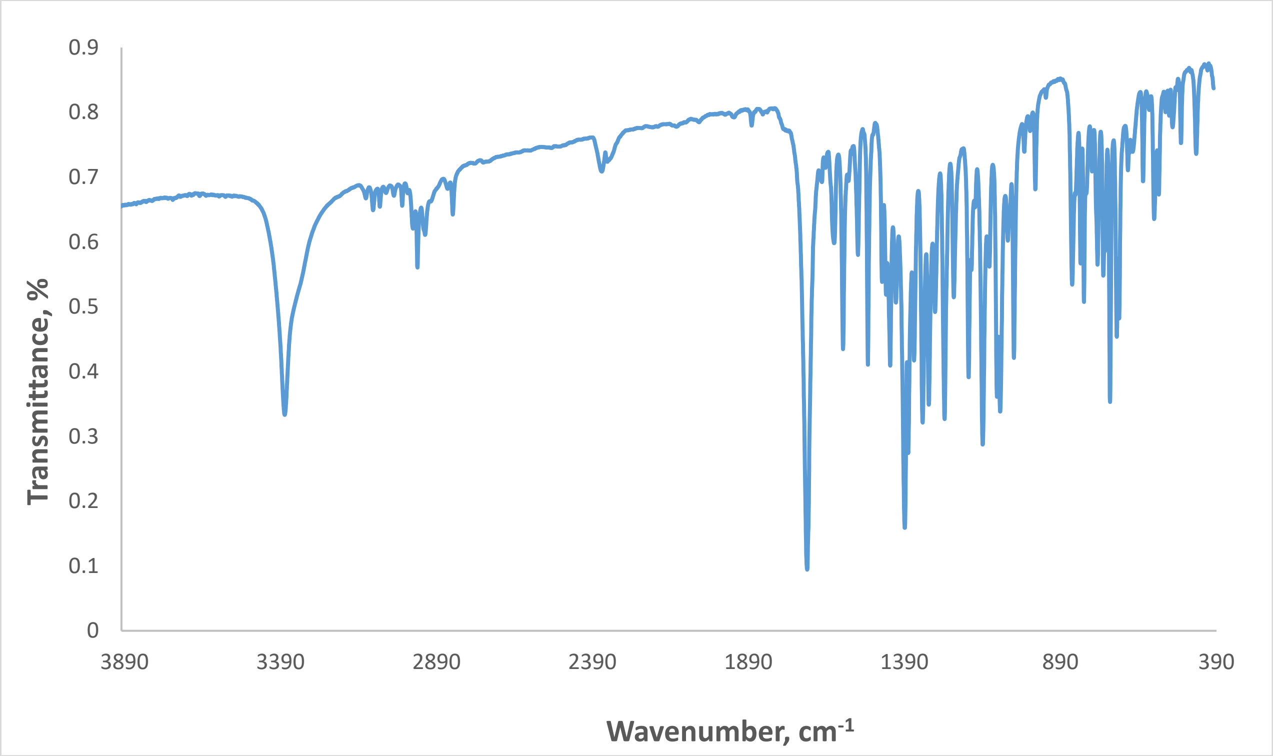

Figure S3 FTIR spectrum of compound **4a**, KBr tablet, cm^{-1}

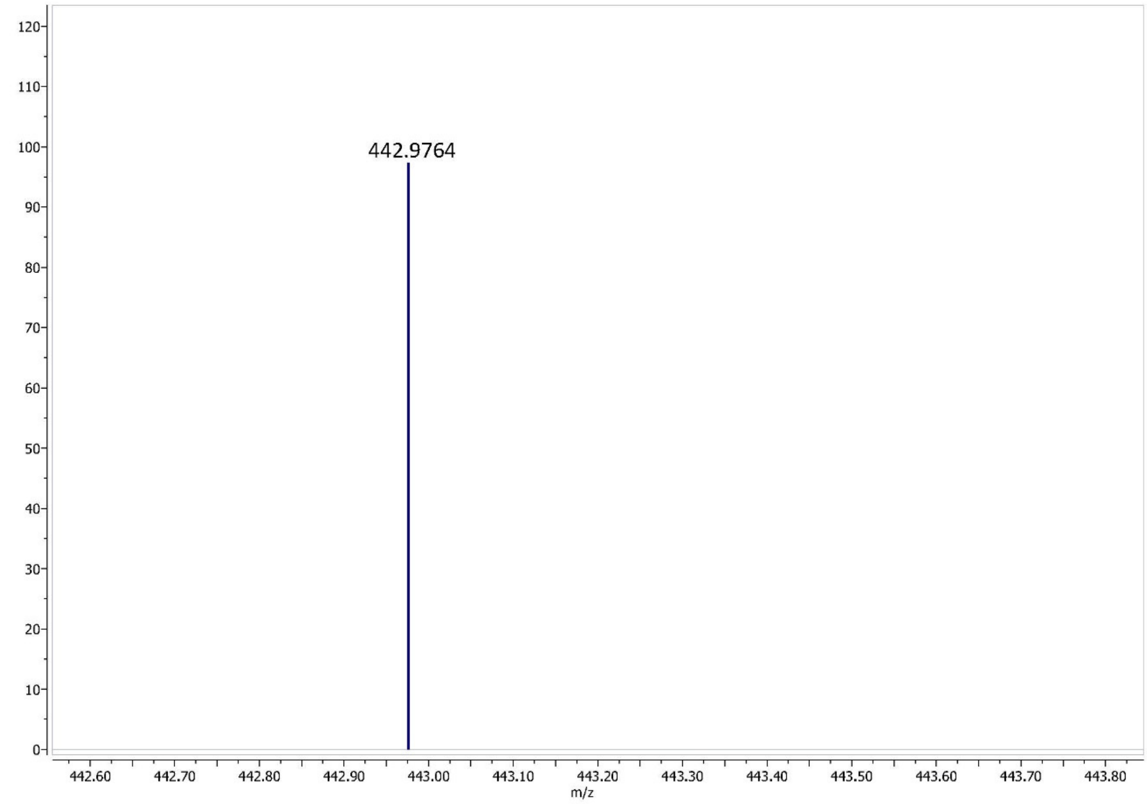

Figure S4 ESI-HRMS spectrum of compound **4a**, positive mode

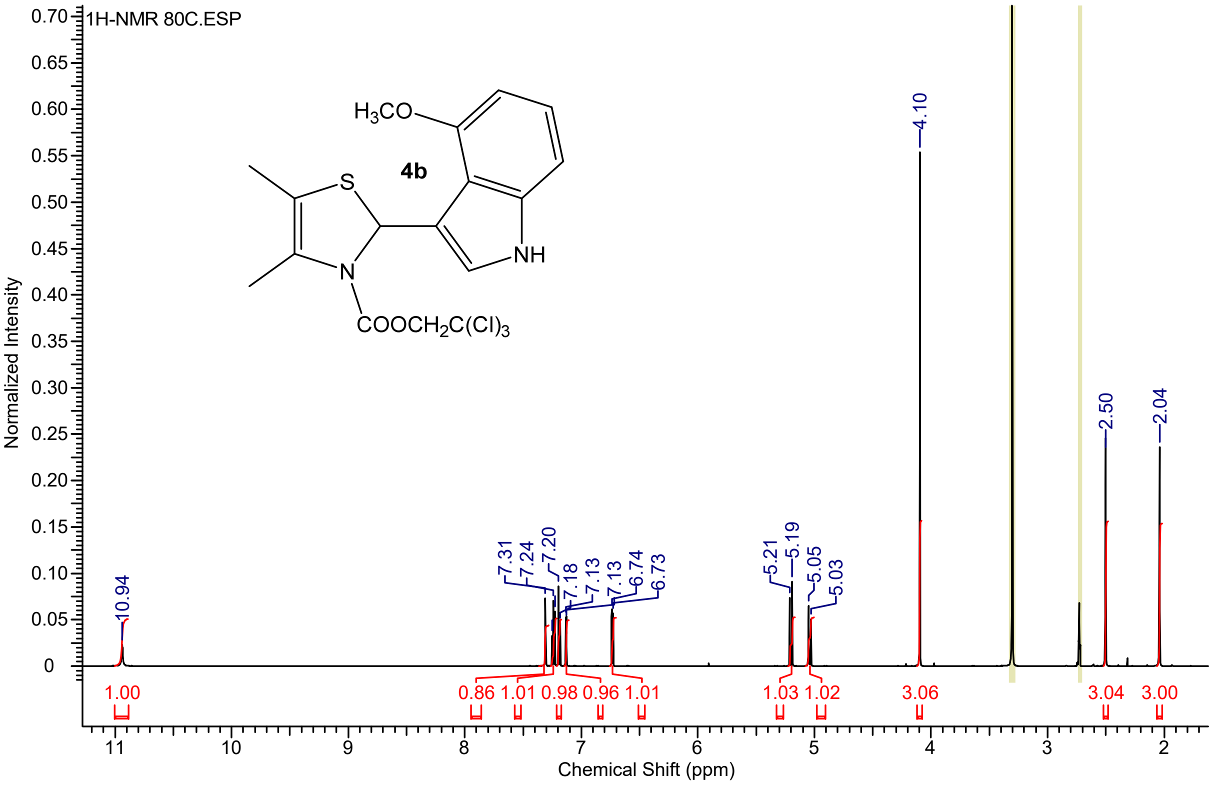

Figure S5 ^{1}H-NMR spectrum of **4b** - DMSO-d_6, 80 °C, 600 MHz

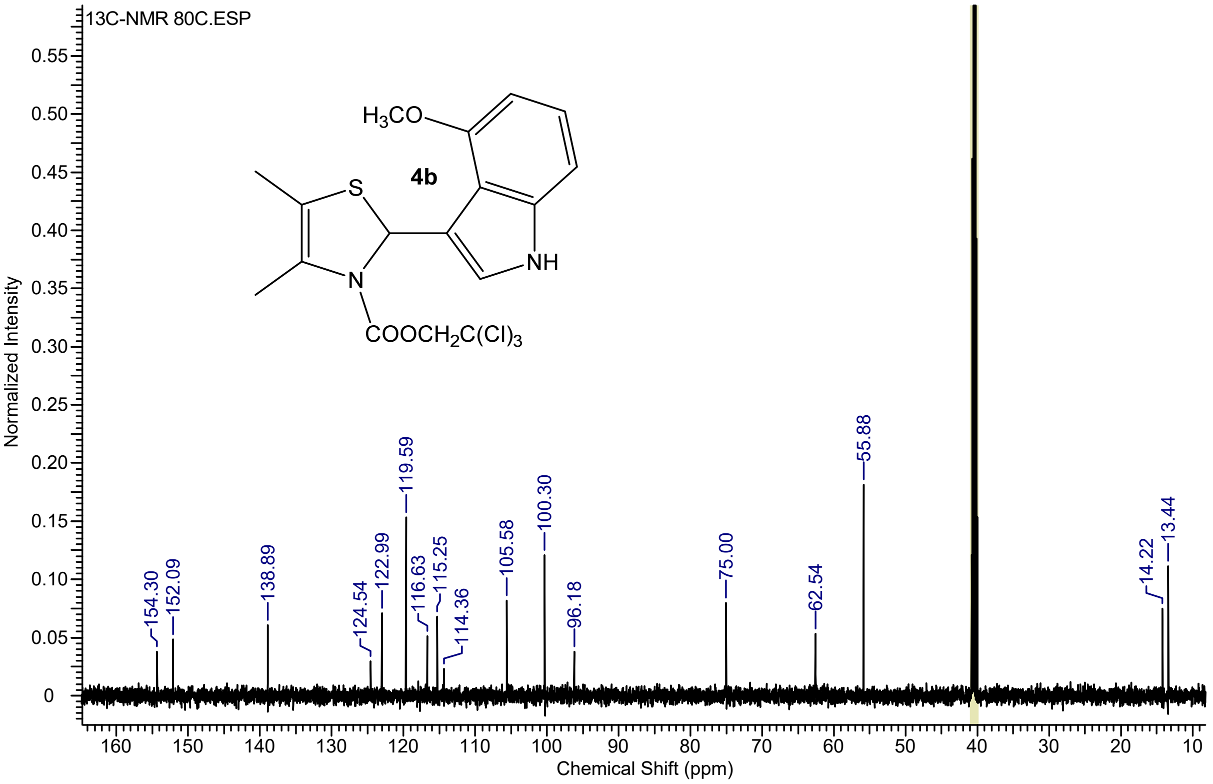

Figure S6 ^{13}C{^{1}H}-NMR spectrum of **4b** - DMSO-d_6, 80 °C, 150 MHz

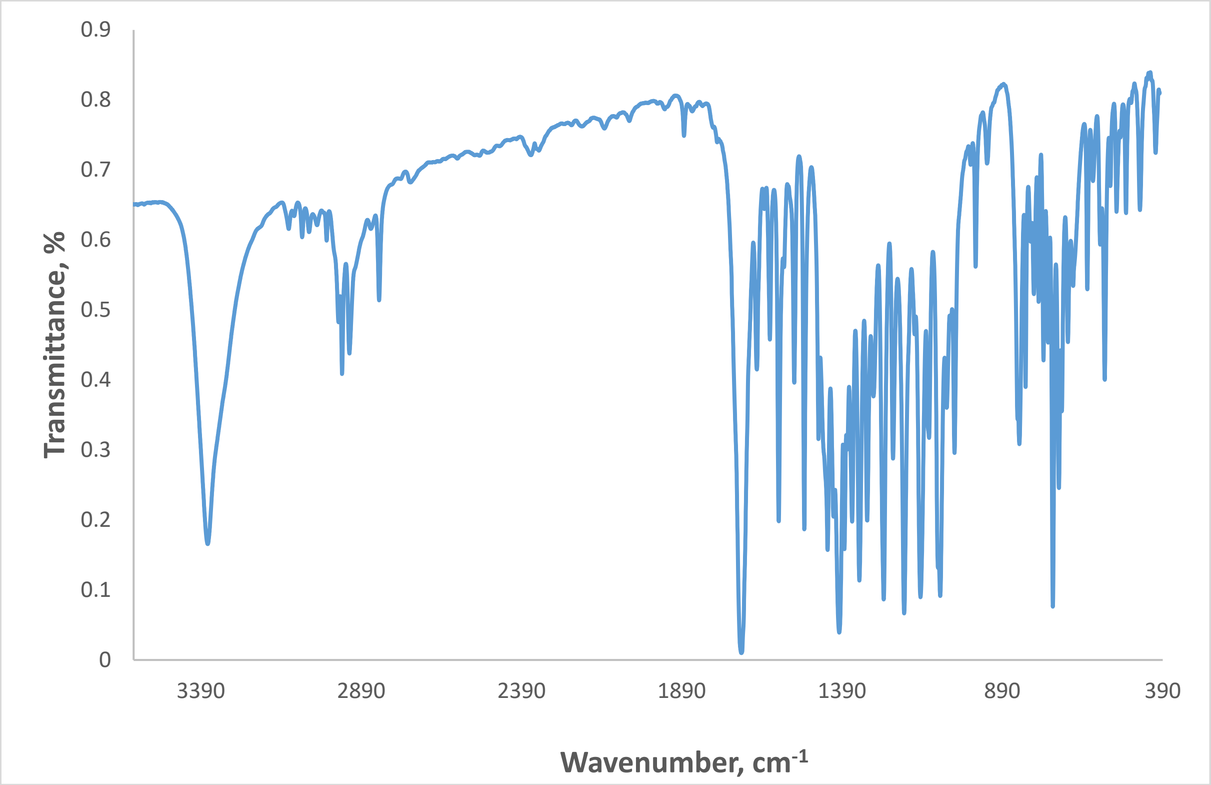

Figure S7 FTIR spectrum of compound **4b**, KBr tablet, cm^{-1}

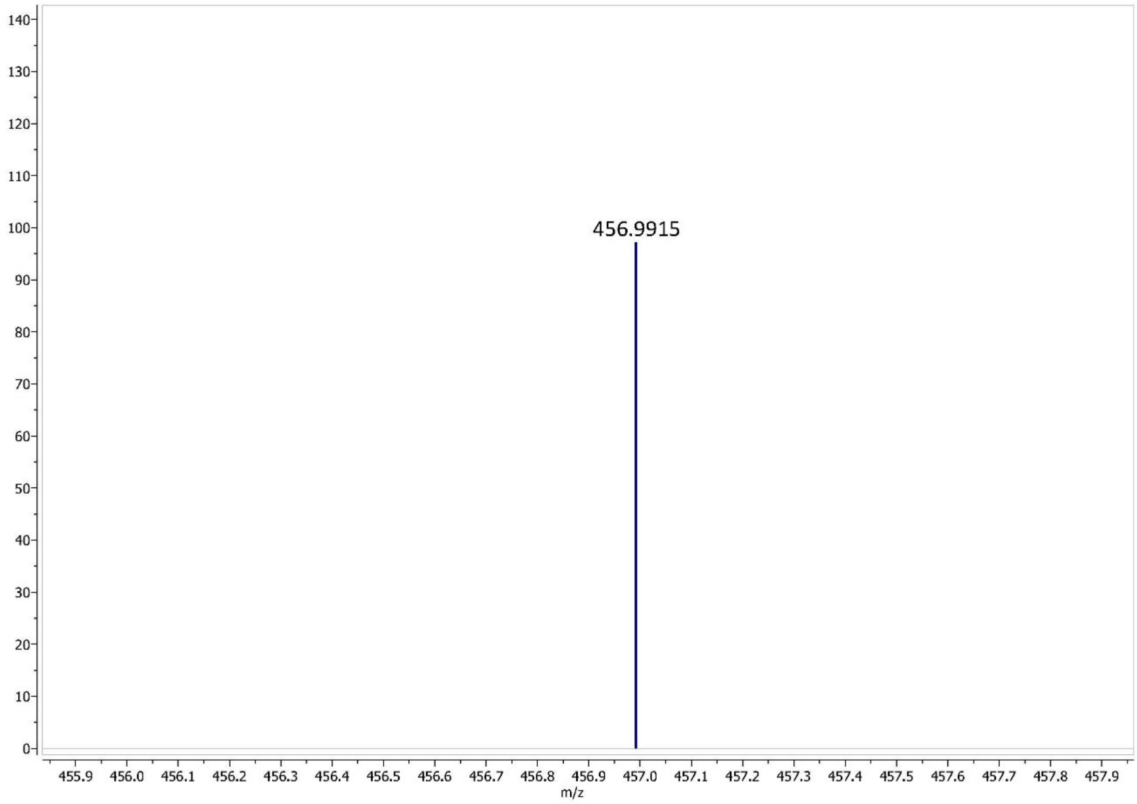

Figure S8 ESI-HRMS spectrum of compound **4b**, positive mode

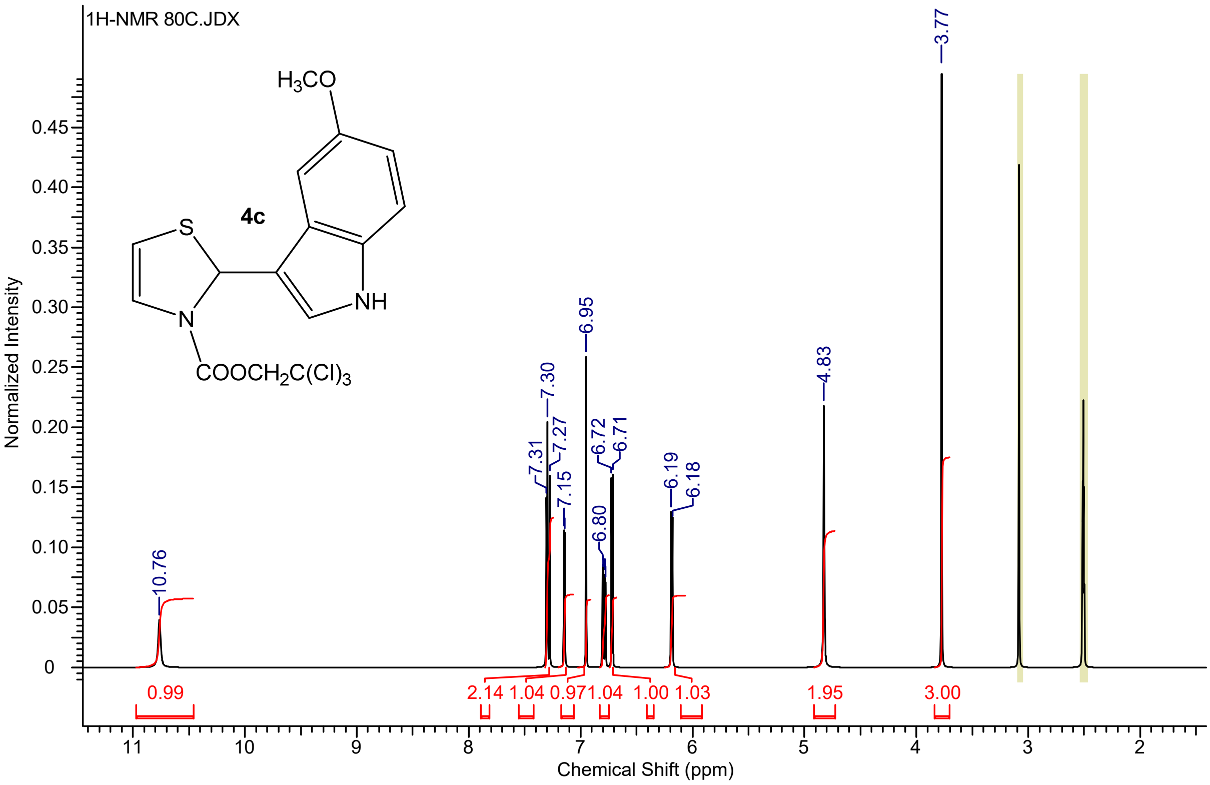

Figure S9 ^{1}H-NMR spectrum of **4c** - DMSO-d6, 80 °C, 400 MHz

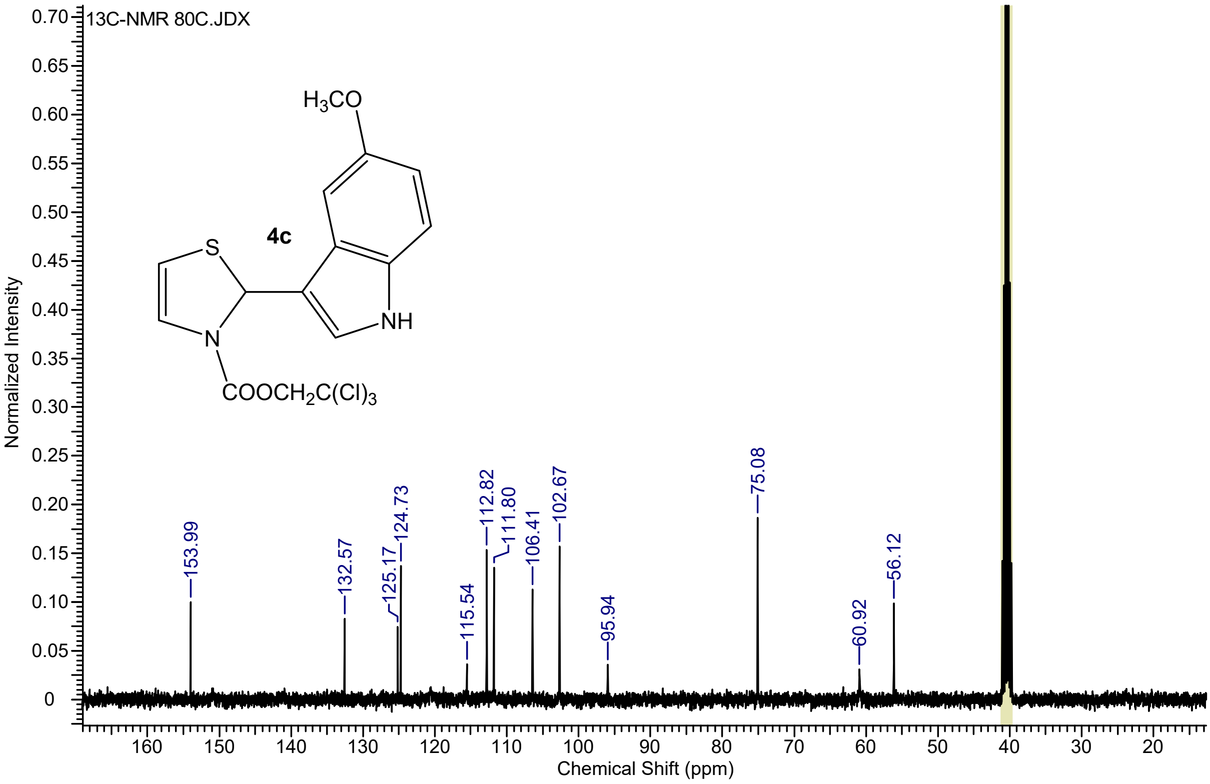

Figure S10 ^{13}C{^{1}H}-NMR spectrum of **4c** - DMSO-d6, 80 °C, 100 MHz

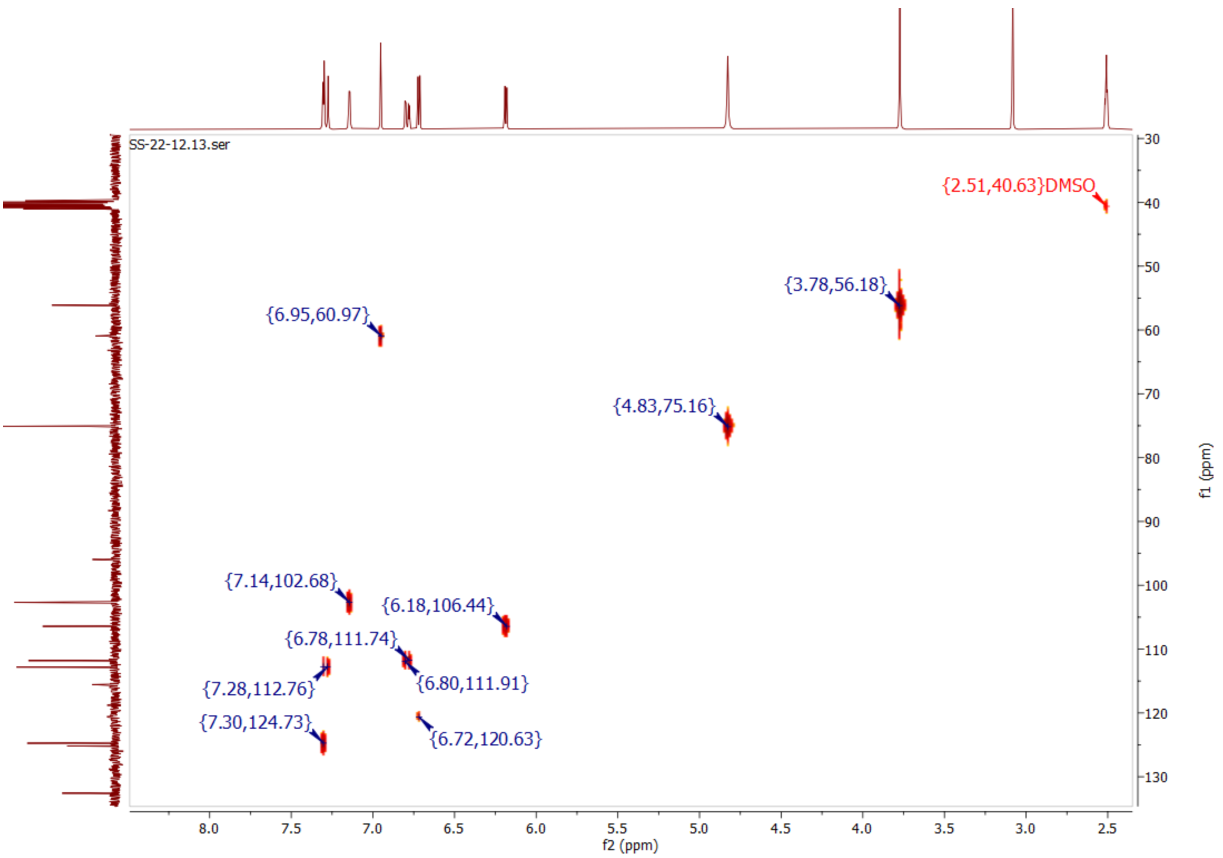

Figure S11 HSQC-NMR spectrum of **4c** - DMSO-d_6, 80 ^{o}C

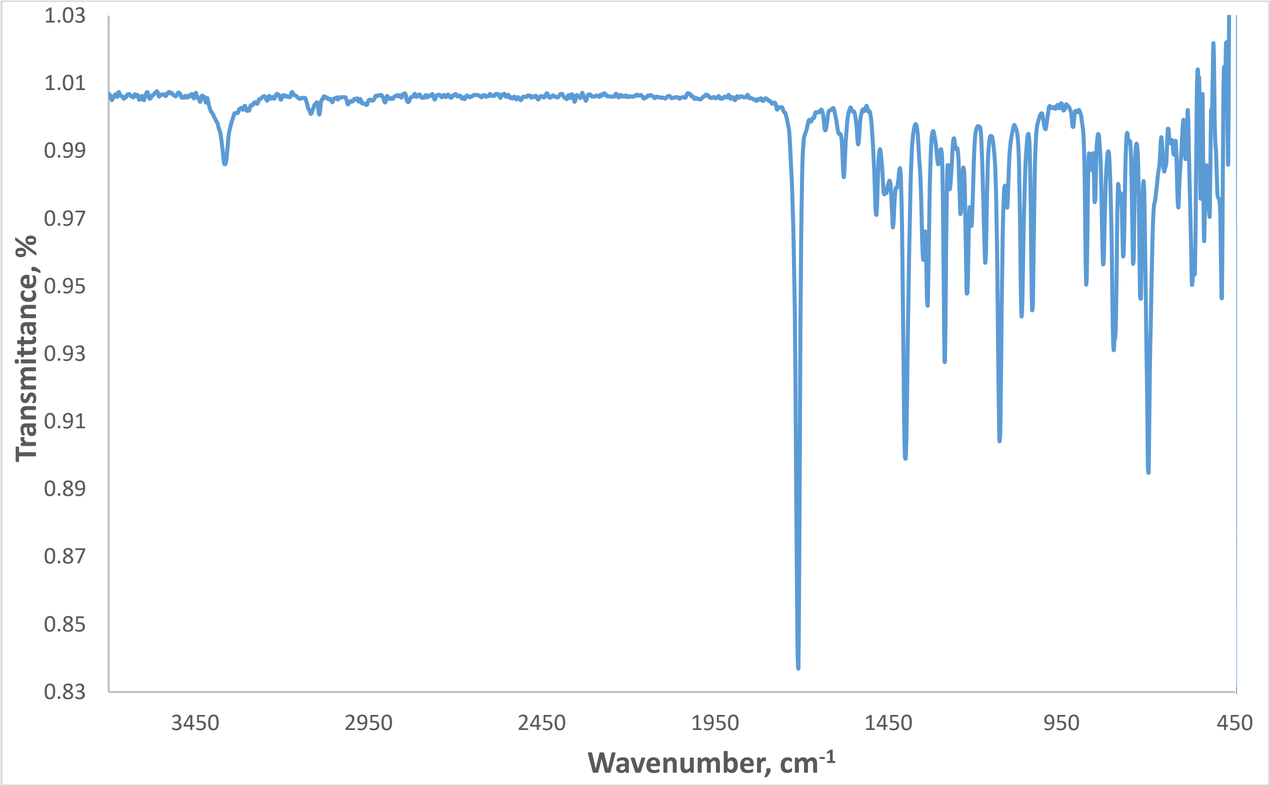

Figure S12 ATR-FTIR spectrum of compound **4c**, cm^{-1}

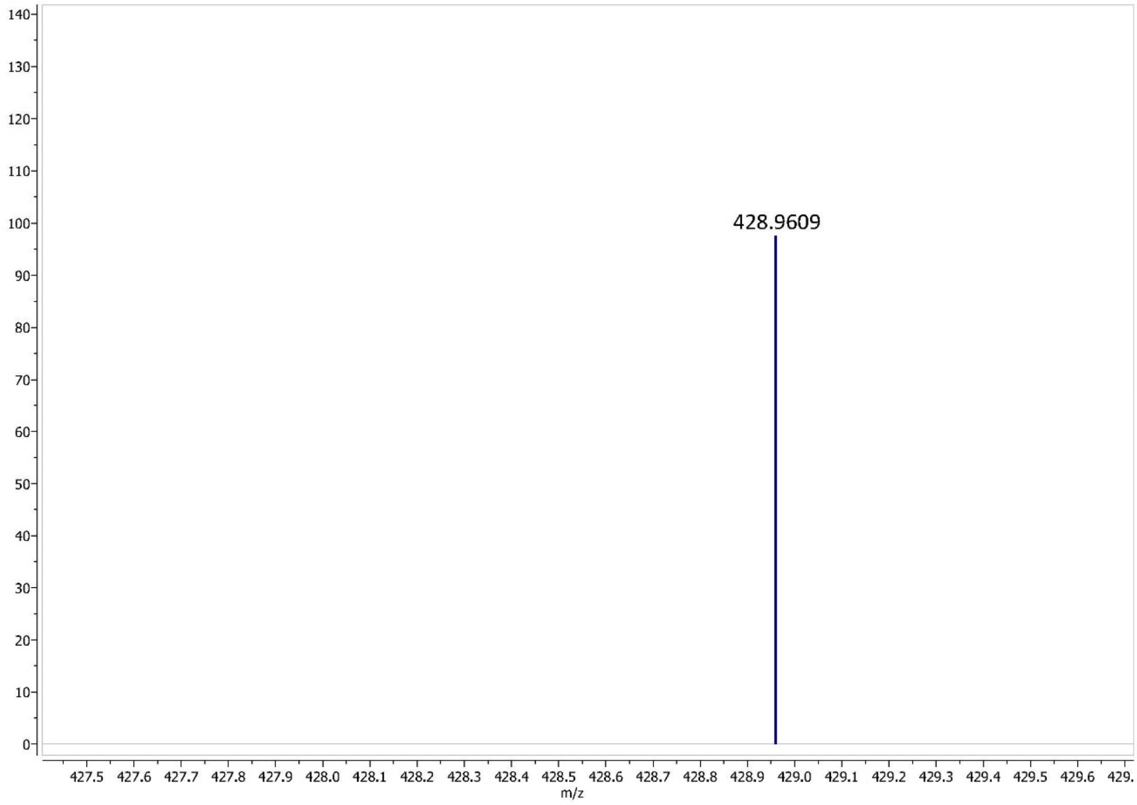

Figure S13 ESI-HRMS spectrum of compound **4c**, positive mode

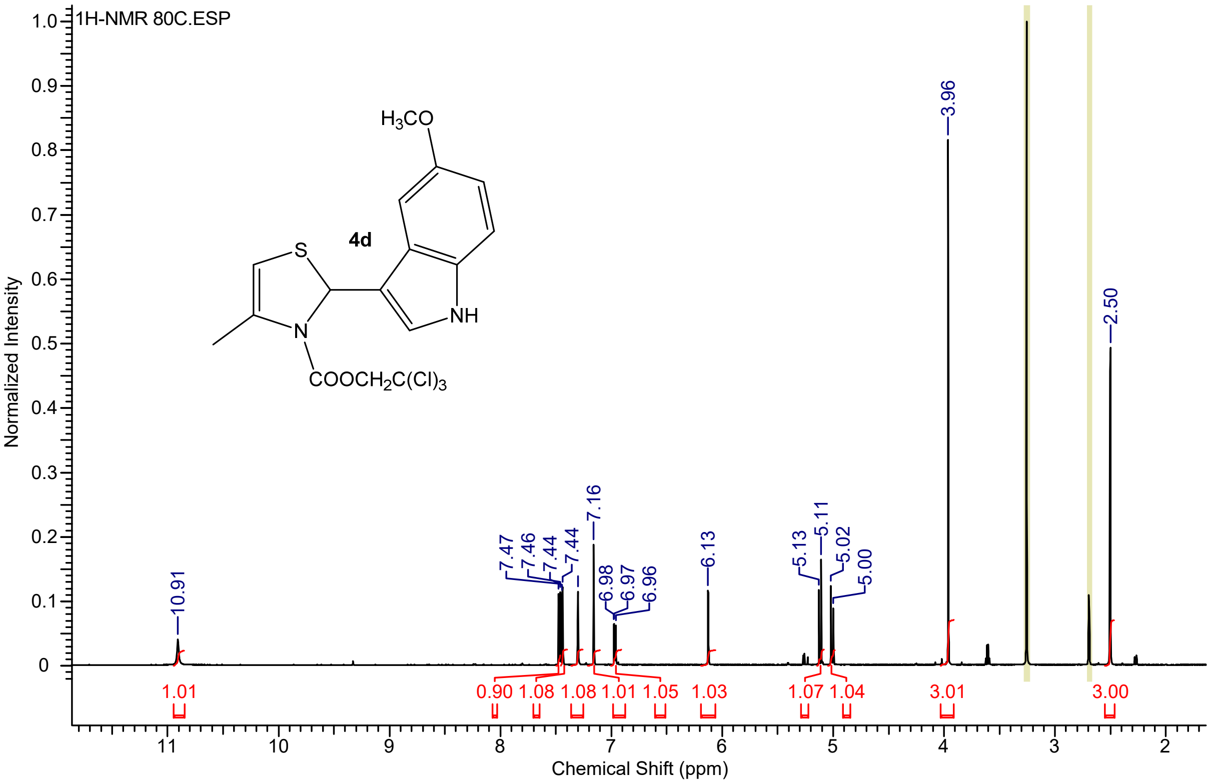

Figure S14 ^{1}H-NMR spectrum of **4d** - DMSO-d$_6$, 80 ^{o}C, 600 MHz

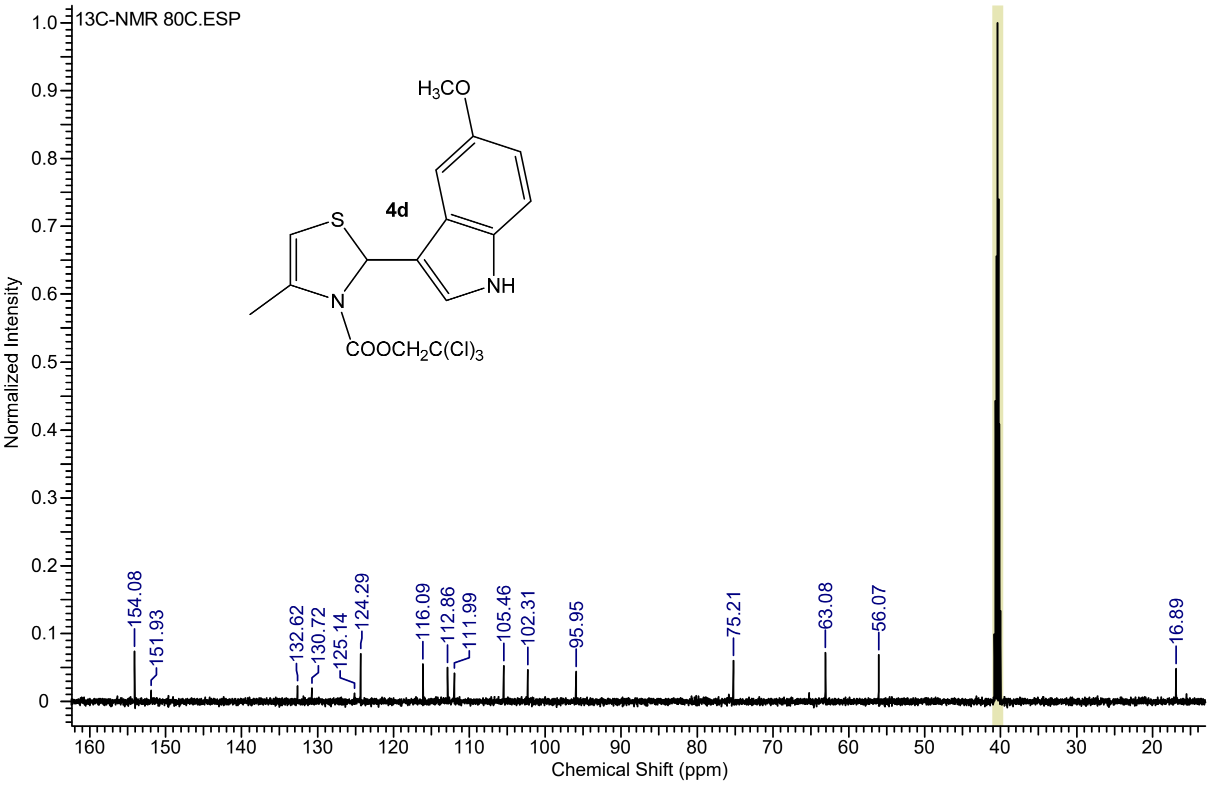

Figure S15 $^{13}C\{^{1}H\}$-NMR spectrum of **4d** - DMSO-d_6, 80 ^{o}C, 150 MHz

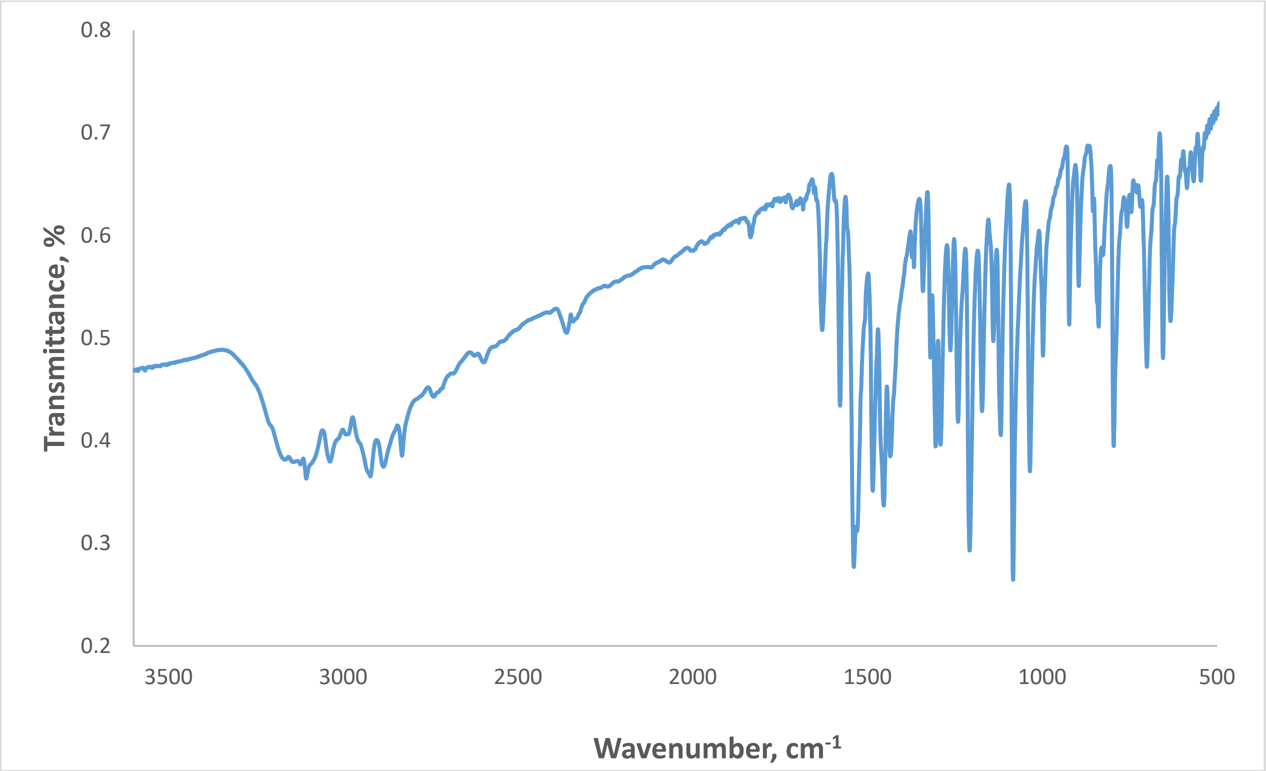

Figure S16 FTIR spectrum of compound **4d**, KBr tablet, cm^{-1}

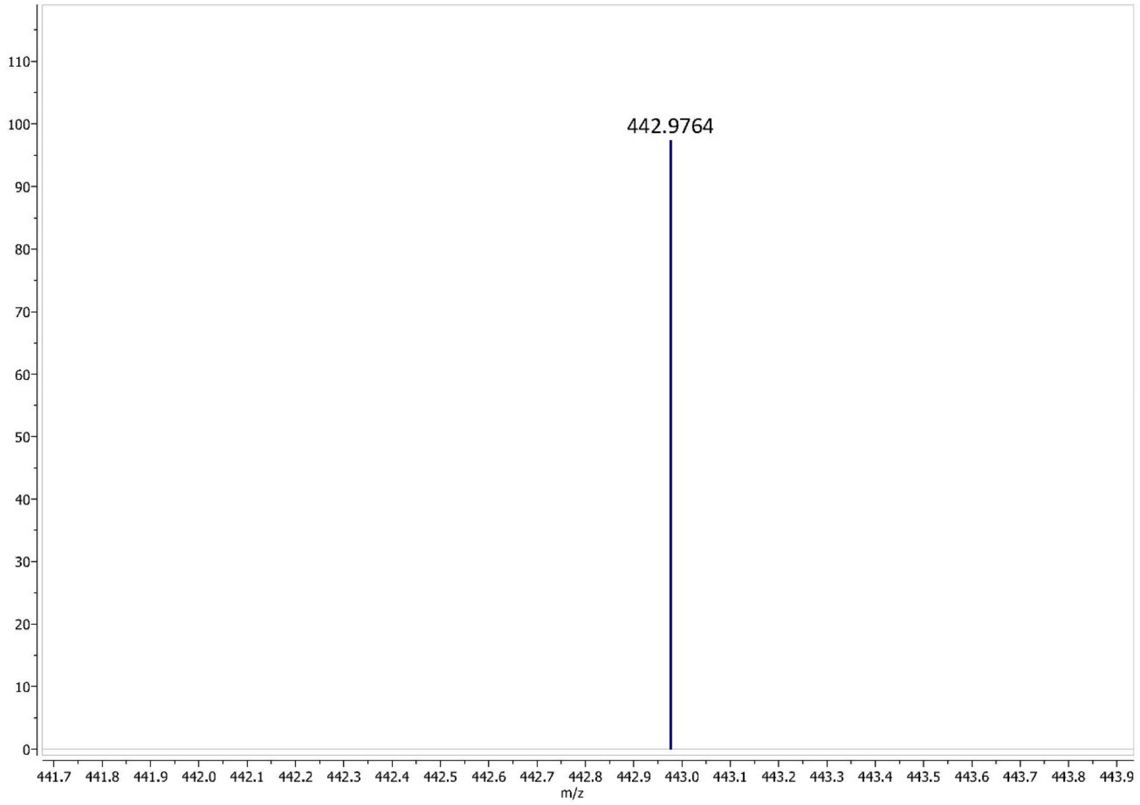

Figure S17 ESI-HRMS spectrum of compound **4d**, positive mode

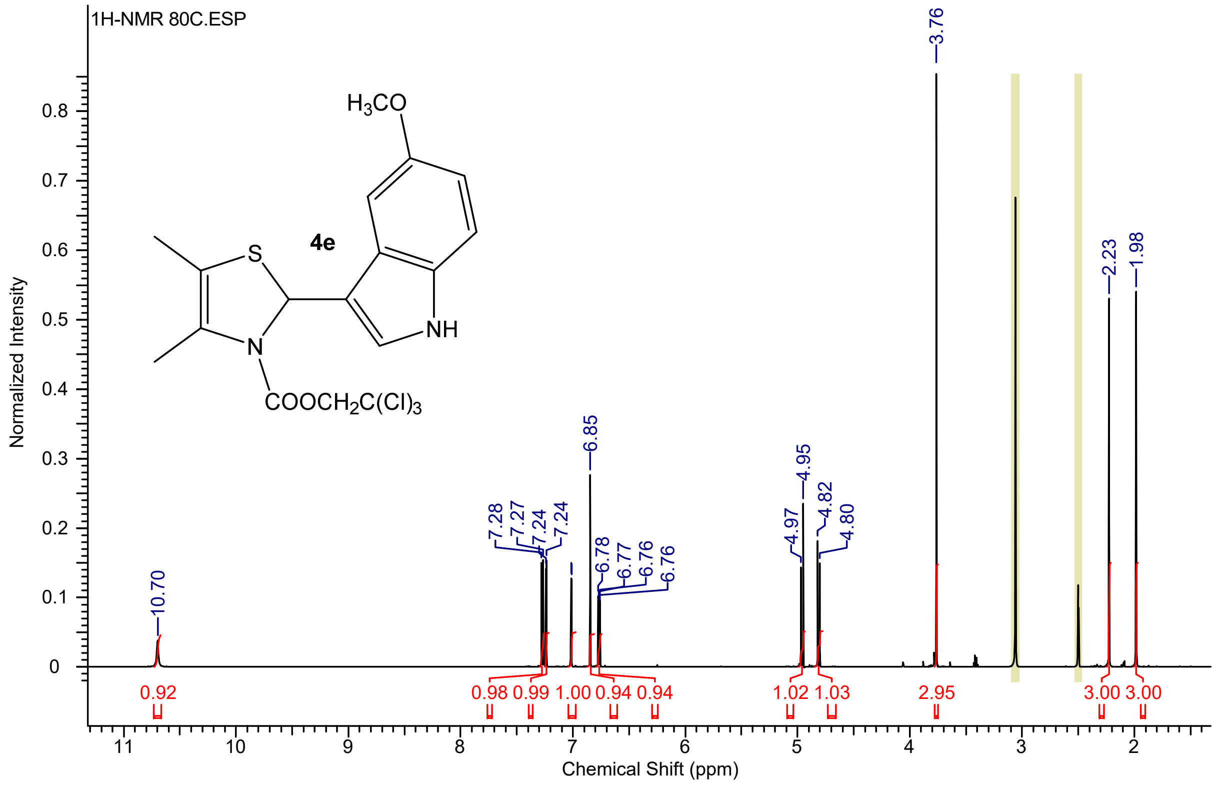

Figure S18 ^{1}H-NMR spectrum of **4e** - DMSO-d_6, 80 ^{o}C, 600 MHz

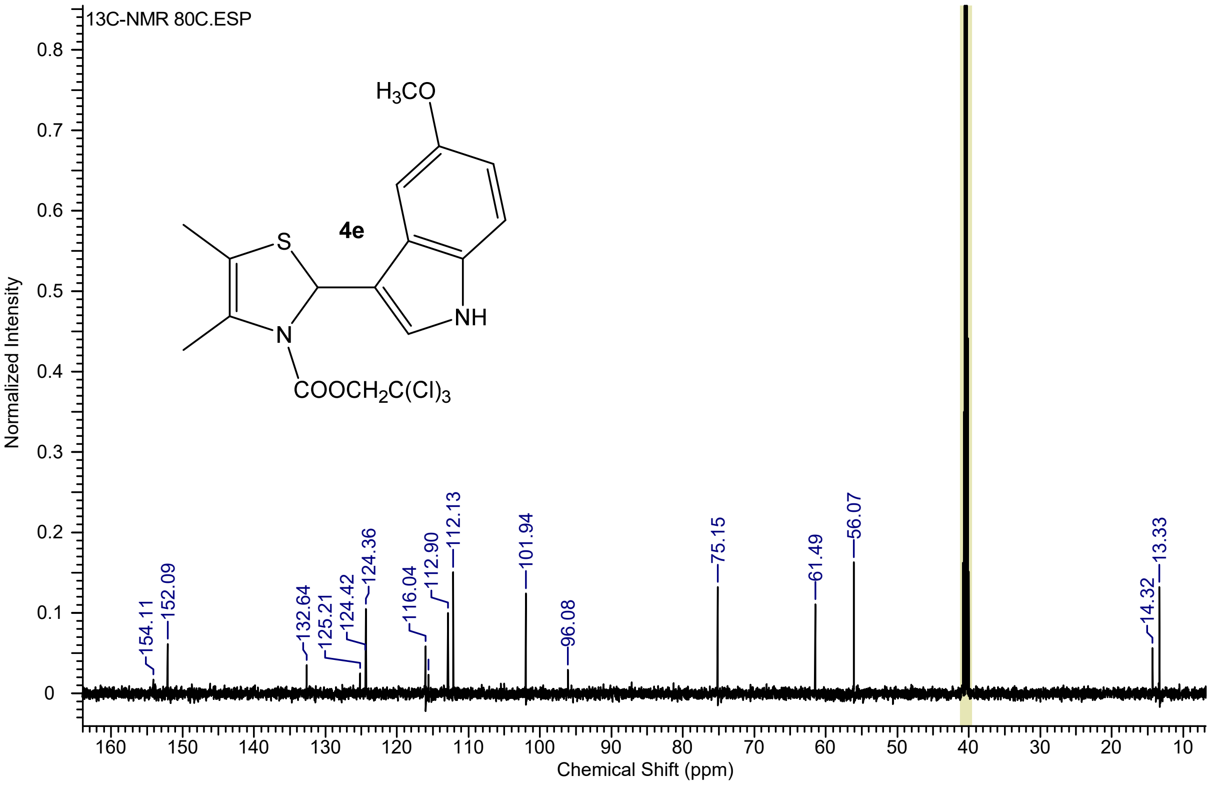

Figure S19 $^{13}C\{^{1}H\}$-NMR spectrum of **4e** - DMSO-d_6, 80 ^{o}C, 150 MHz

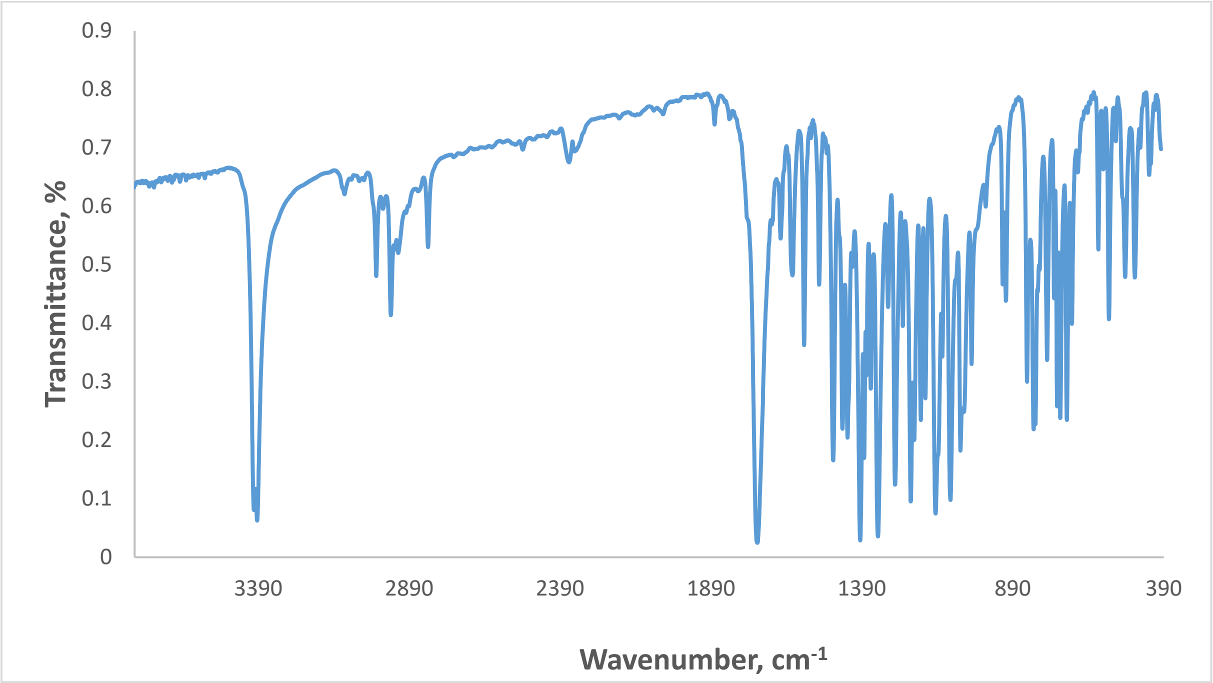

Figure S20 FTIR spectrum of compound **4e**, KBr tablet, cm^{-1}

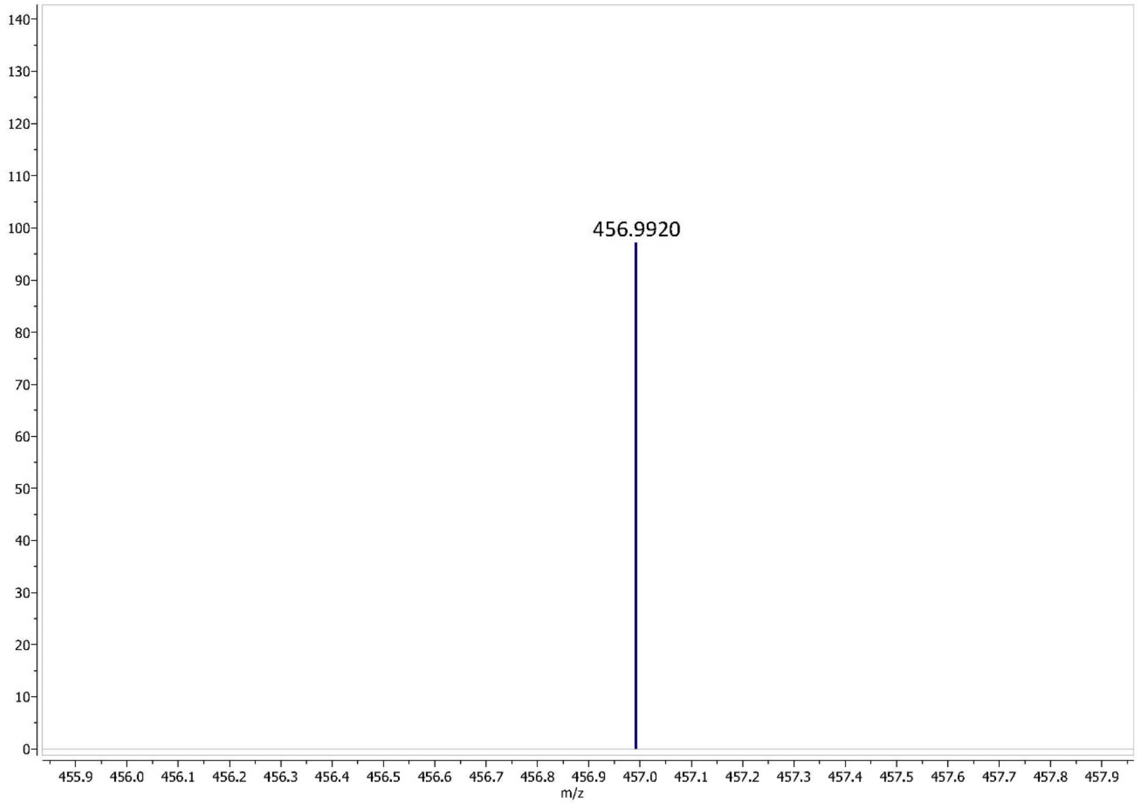

Figure S21 ESI-HRMS spectrum of compound **4e**, positive mode

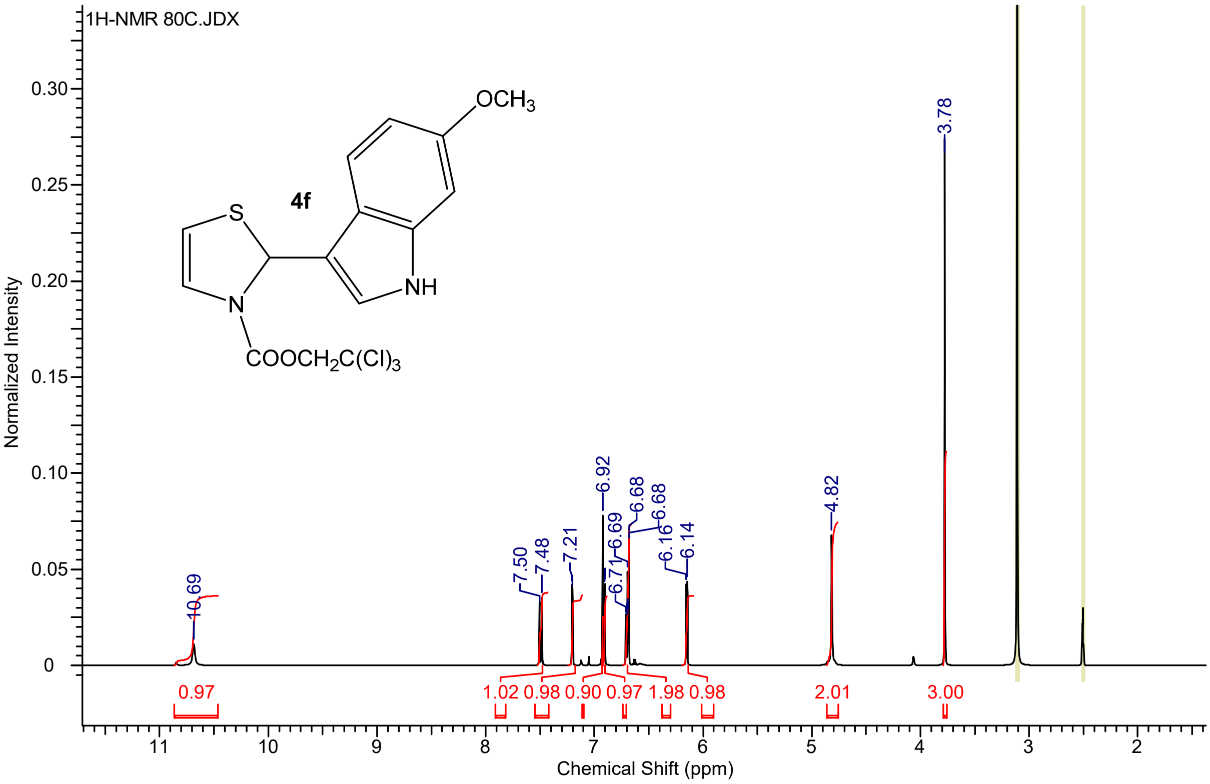

Figure S22 ^{1}H-NMR spectrum of **4f** - DMSO-d$_6$, 80 ^{o}C, 400 MHz

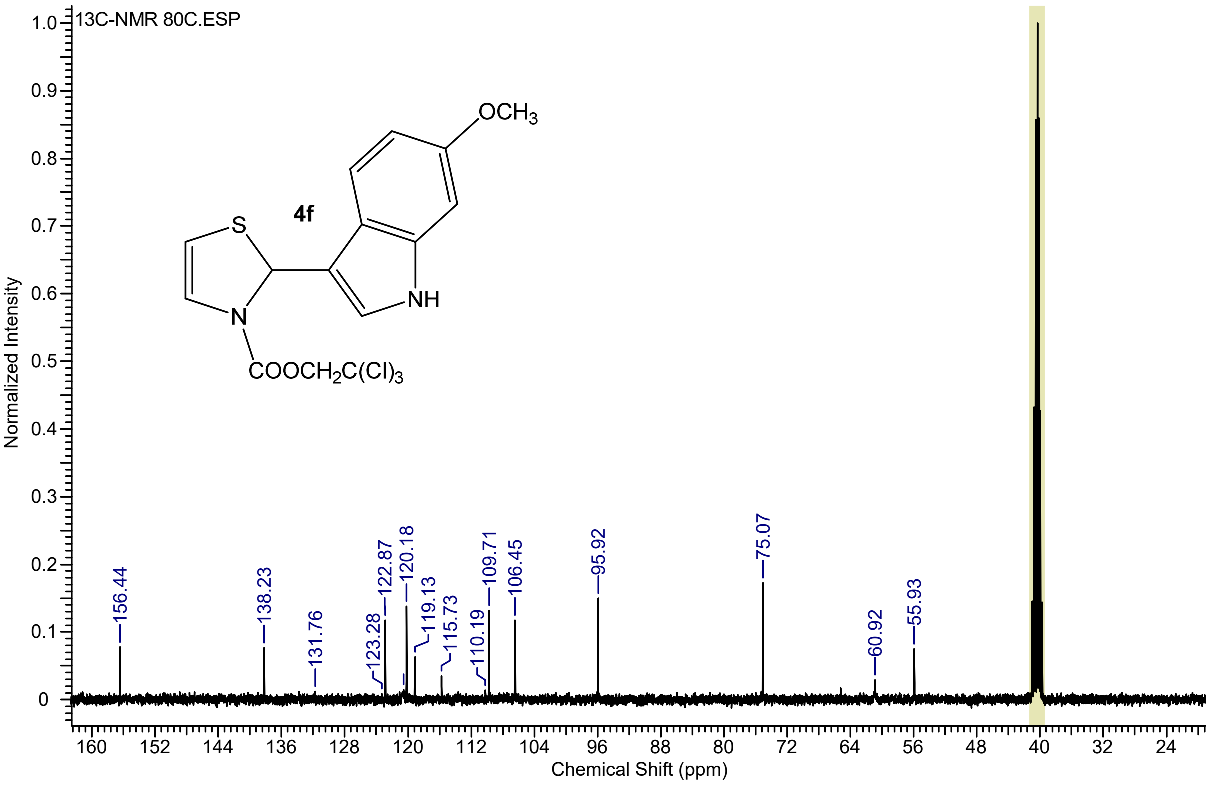

Figure S23 $^{13}C\{^1H\}$-NMR spectrum of **4f** - DMSO-d$_6$, 80 ^oC, 100 MHz

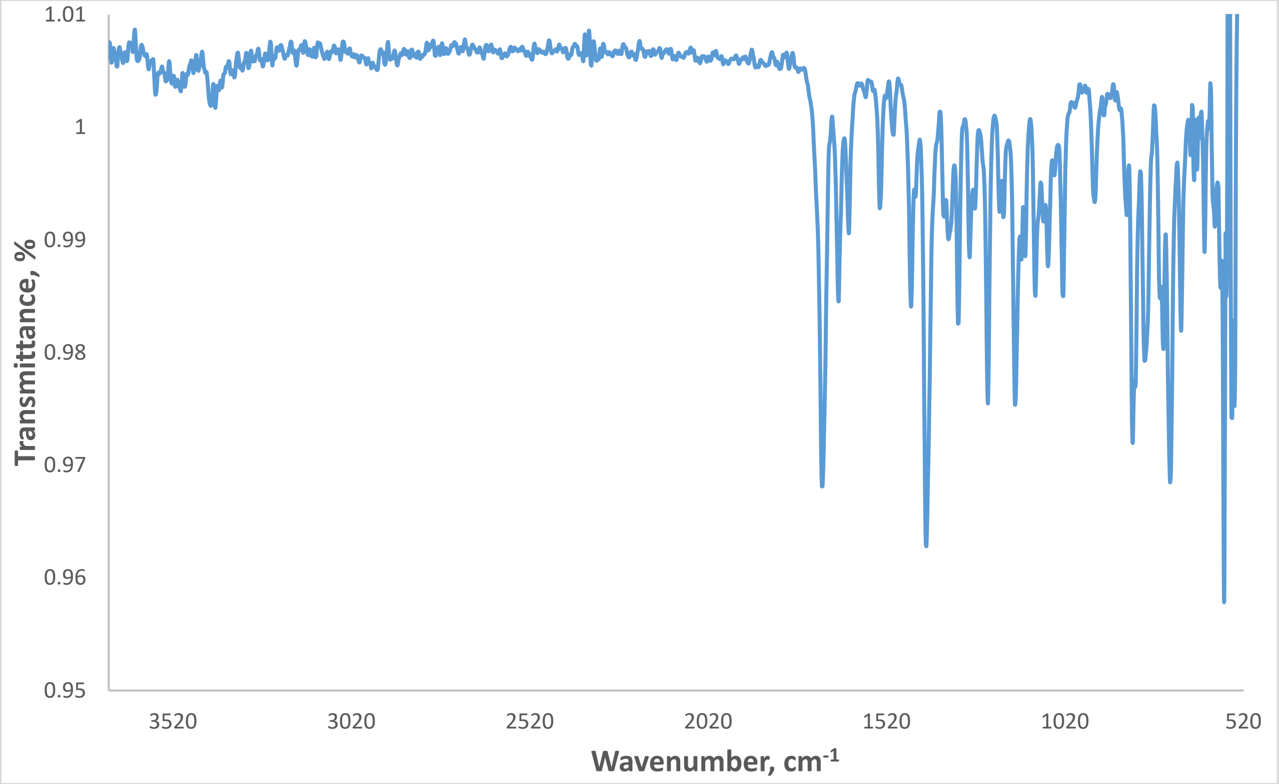

Figure S24 ATR-FTIR spectrum of compound **4f**, cm^{-1}

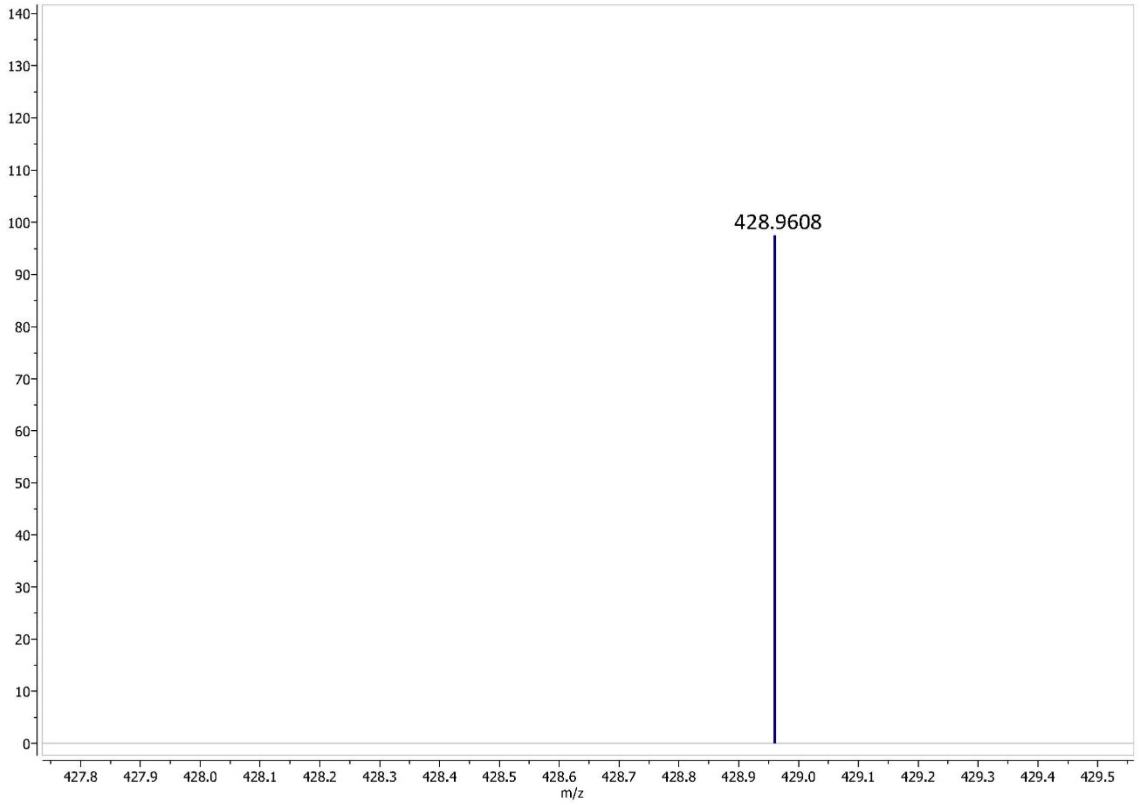

Figure S25 ESI-HRMS spectrum of compound **4f**, positive mode

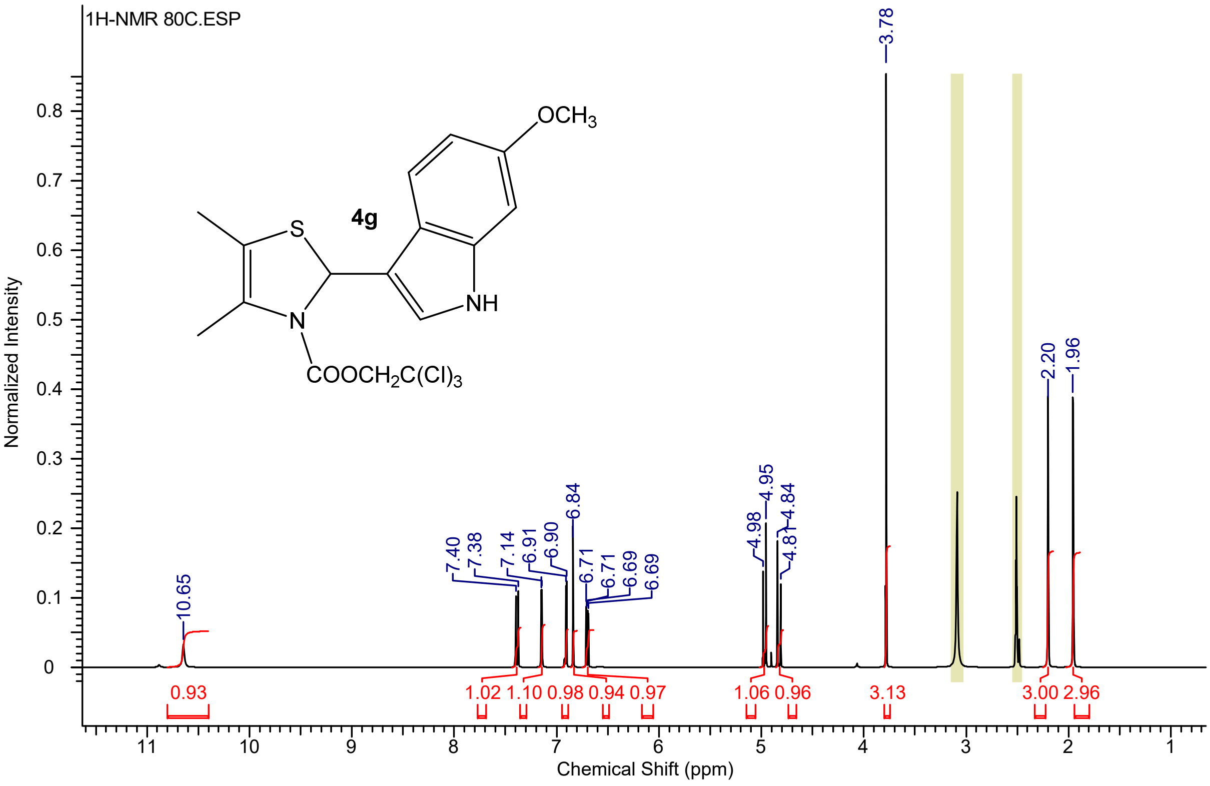

Figure S26 ^{1}H-NMR spectrum of **4g** - DMSO-d$_6$, 80 ^{o}C, 400 MHz

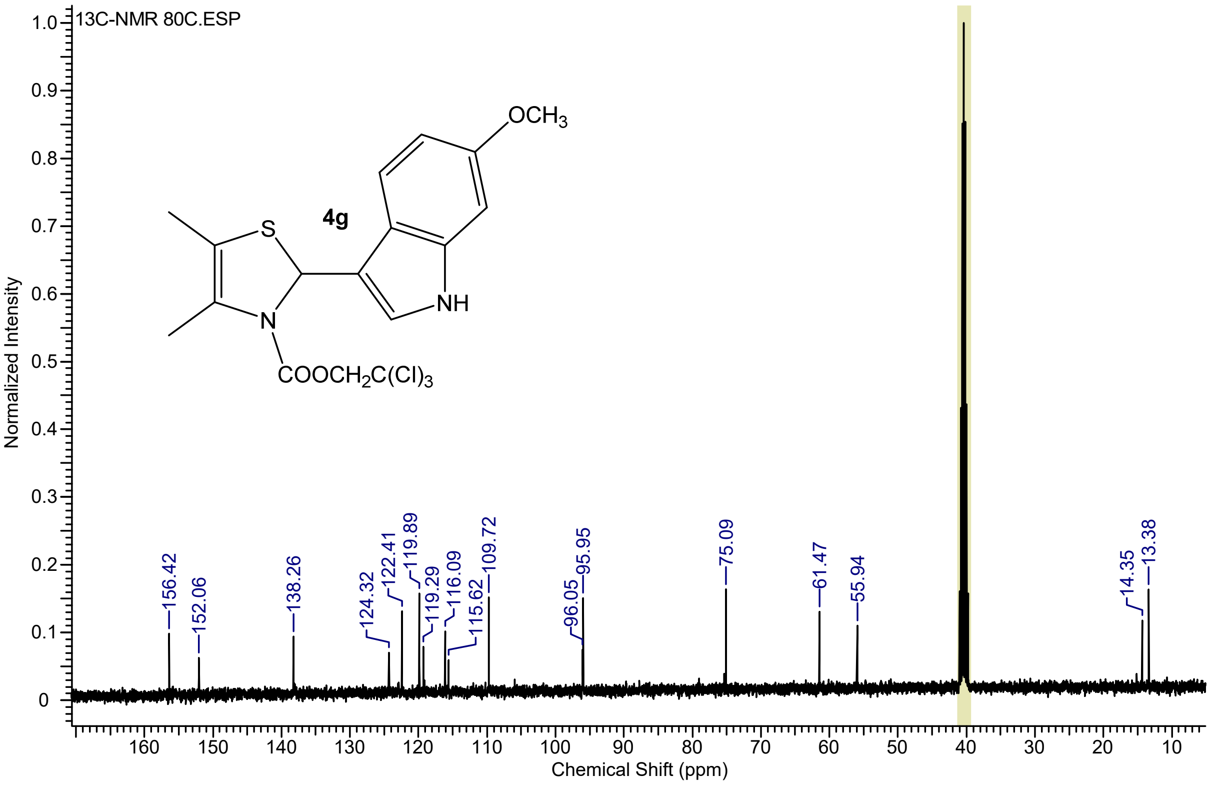

Figure S27 $^{13}C\{^{1}H\}$-NMR spectrum of **4g** - DMSO-d_6, 80 °C, 100 MHz

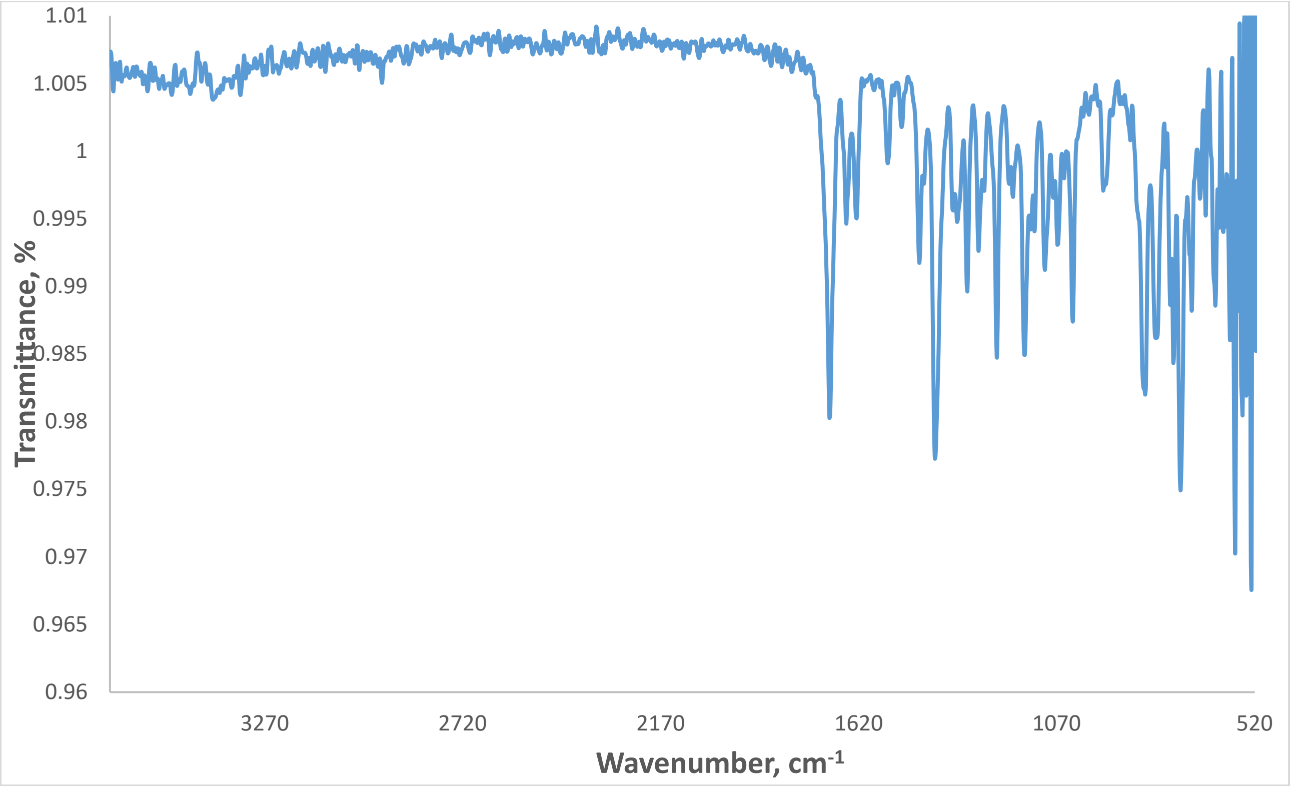

Figure S28 ATR-FTIR spectrum of compound **4g**, cm^{-1}

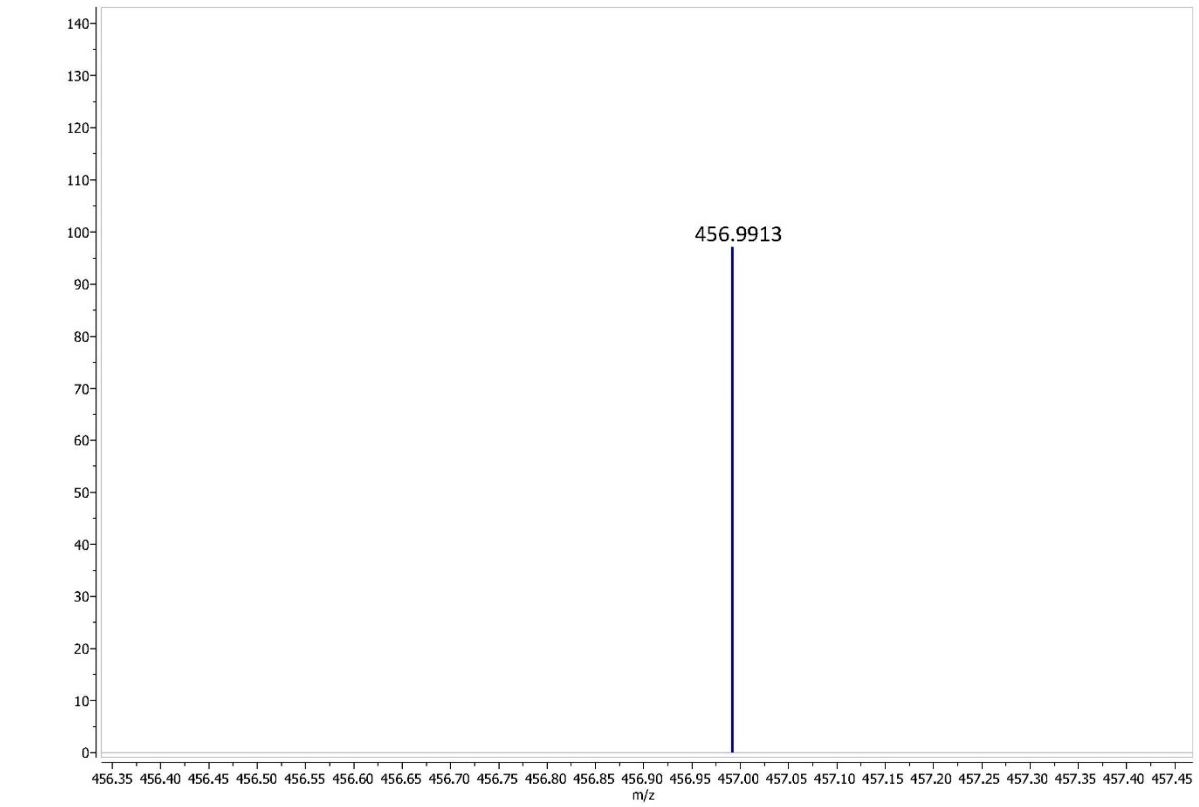

Figure S29 ESI-HRMS spectrum of compound **4g**, positive mode

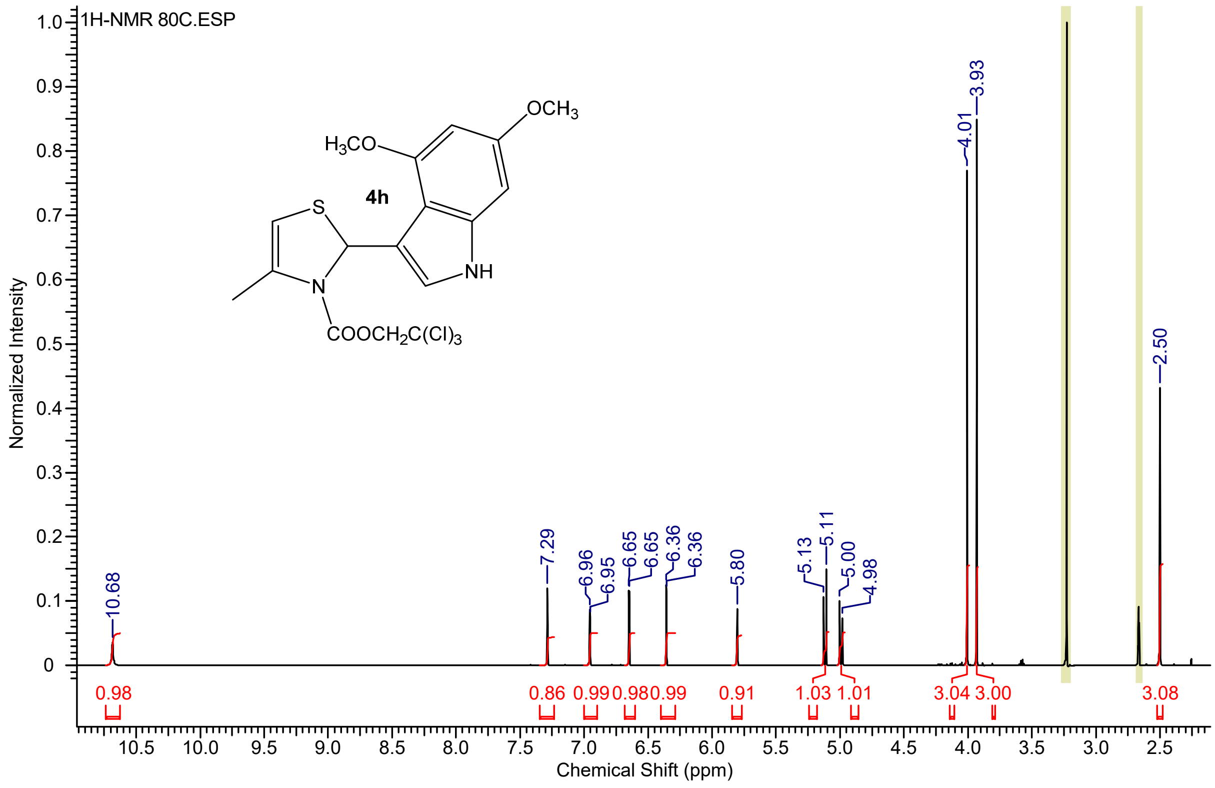

Figure S30 ^{1}H-NMR spectrum of **4h** - DMSO-d$_6$, 80 ^{o}C, 600 MHz

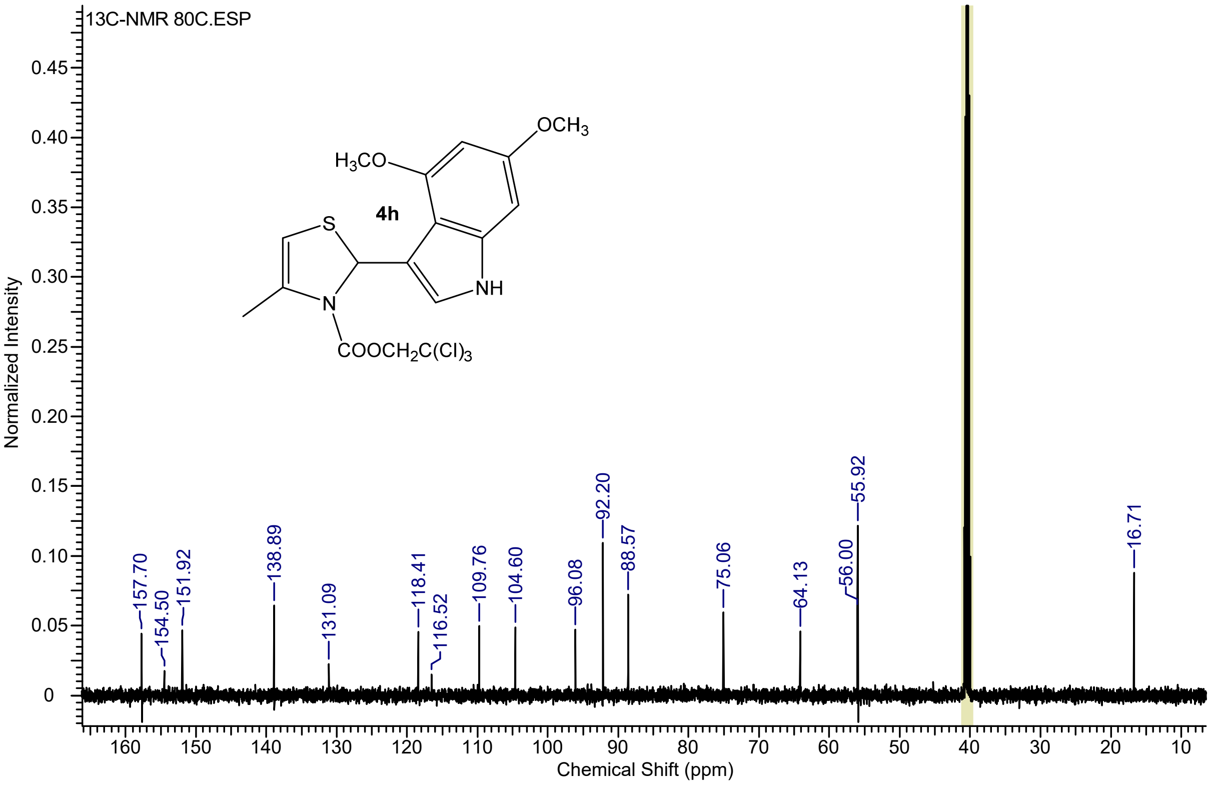

Figure S31 $^{13}C\{^1H\}$-NMR spectrum of **4h** - DMSO-d$_6$, 80 ^{o}C, 150 MHz

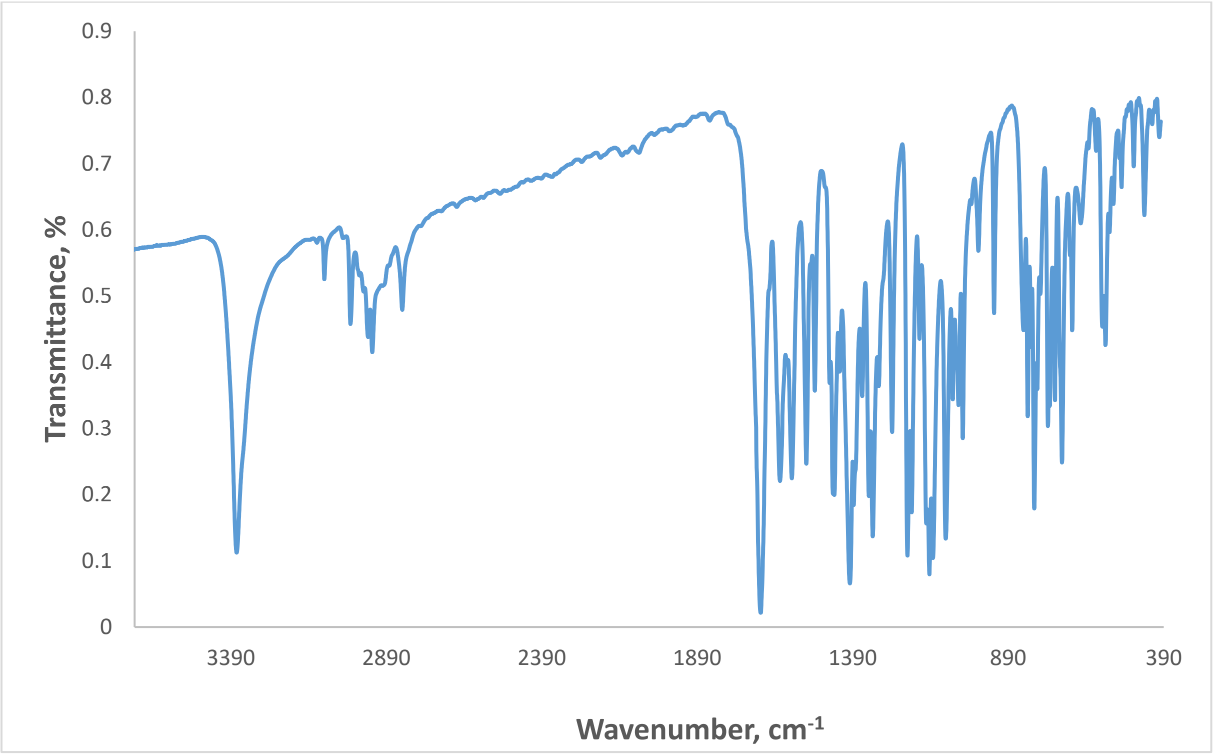

Figure S32 FTIR spectrum of compound **4h**, KBr tablet, cm^{-1}

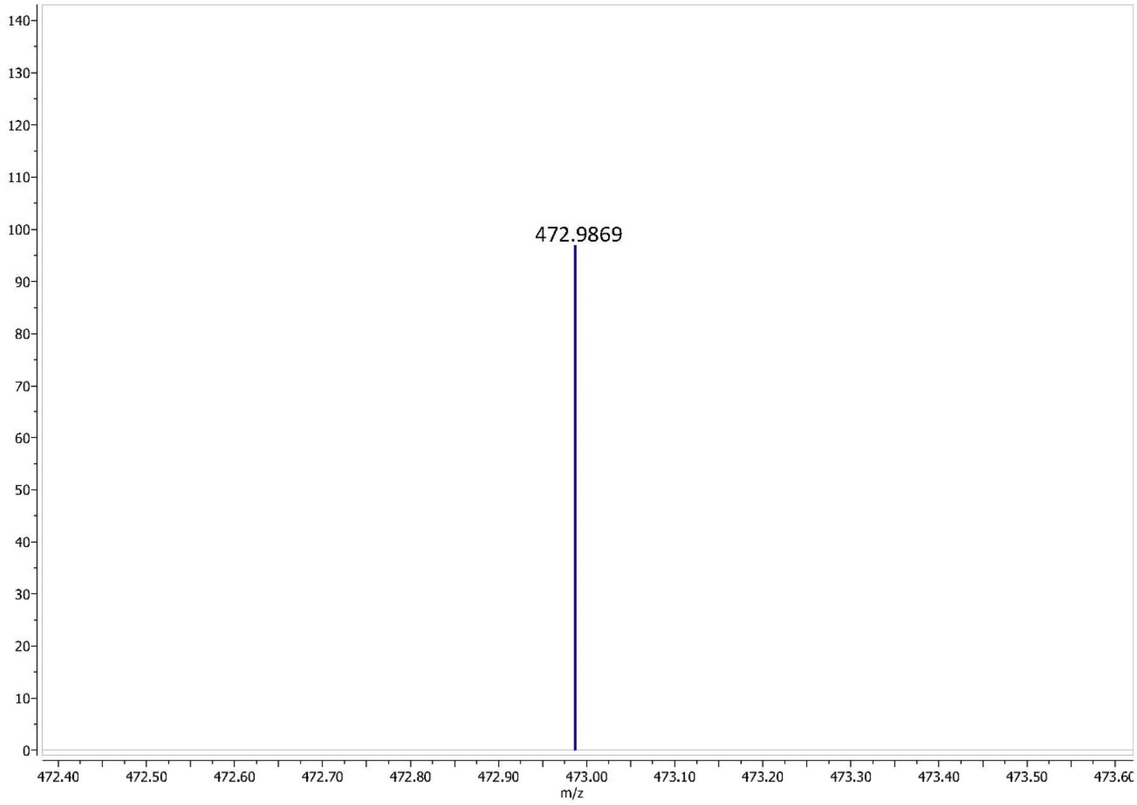

Figure S33 ESI-HRMS spectrum of compound **4h**, positive mode

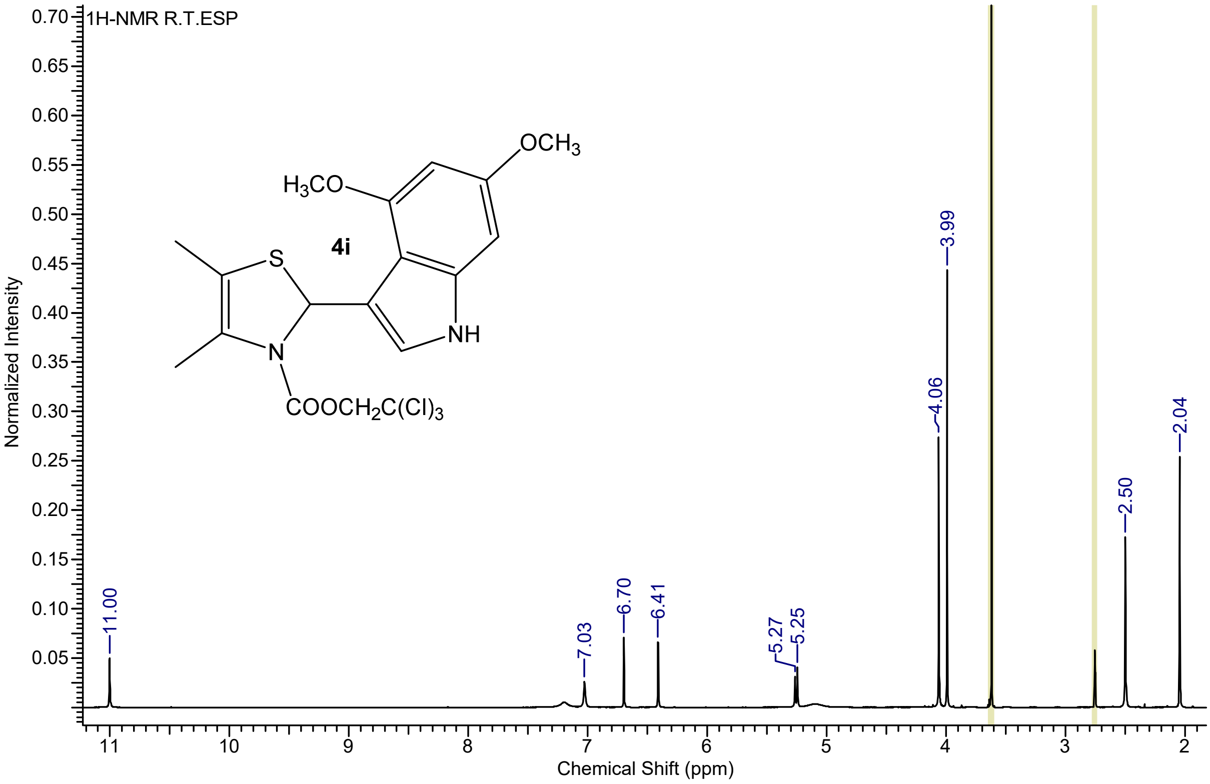

Figure S34 ^{1}H-NMR spectrum of **4i** - DMSO-d$_6$, r.t., 600 MHz

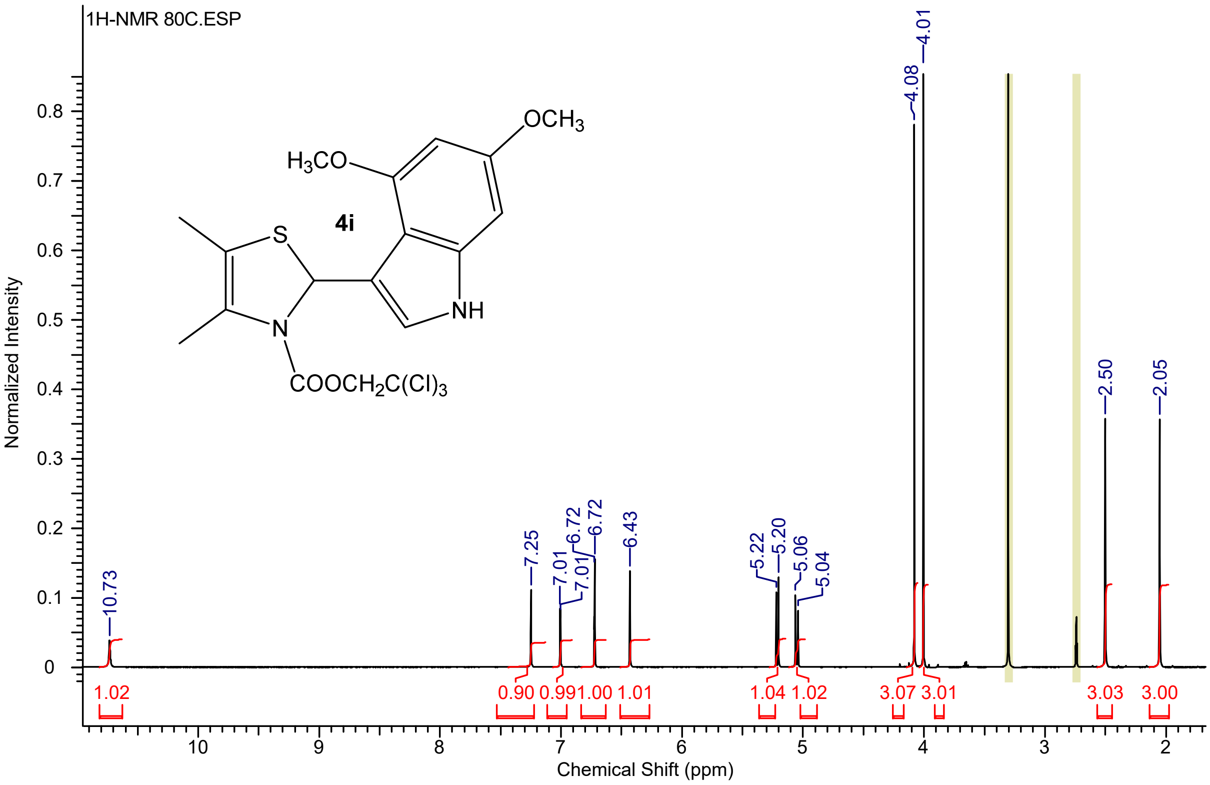

Figure S35 ^{1}H-NMR spectrum of **4i** - DMSO-d_6, 80 ^{o}C, 600 MHz

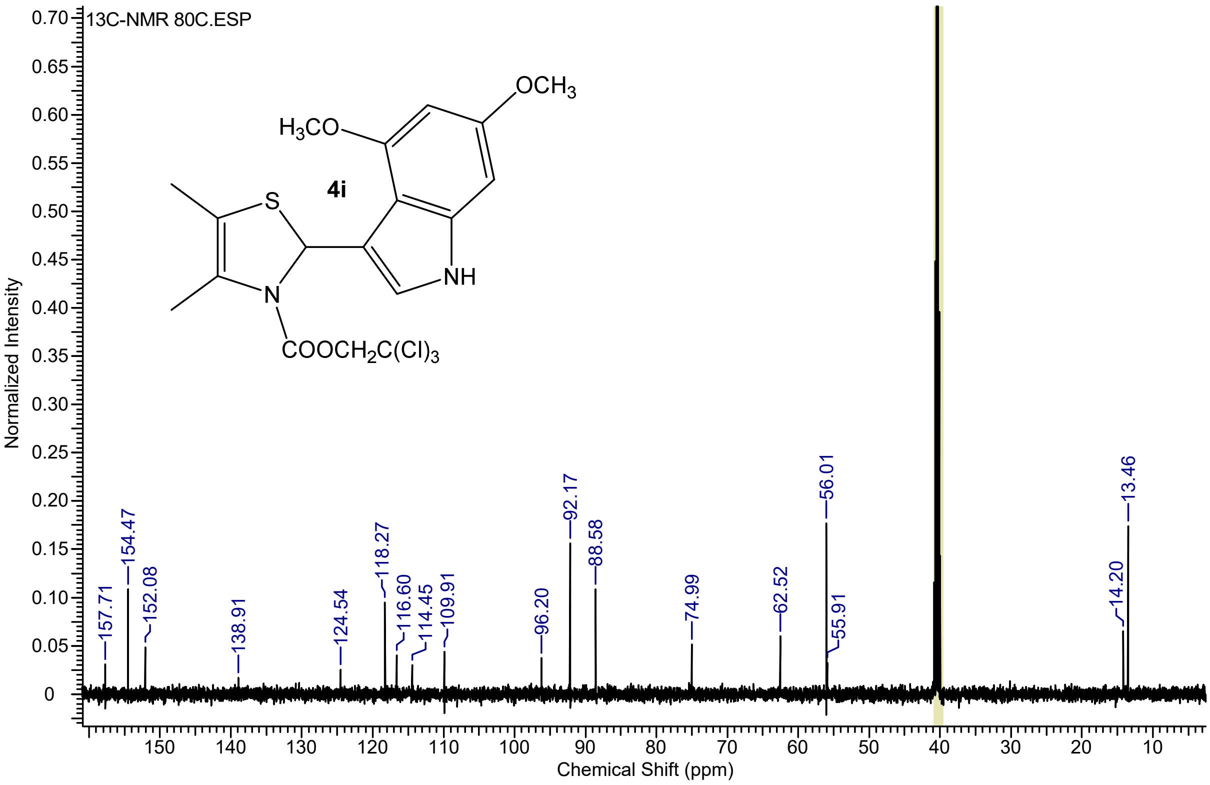

Figure S36 ^{13}C{^{1}H}-NMR spectrum of **4i** - DMSO-d_6, 80 ^{o}C, 150 MHz

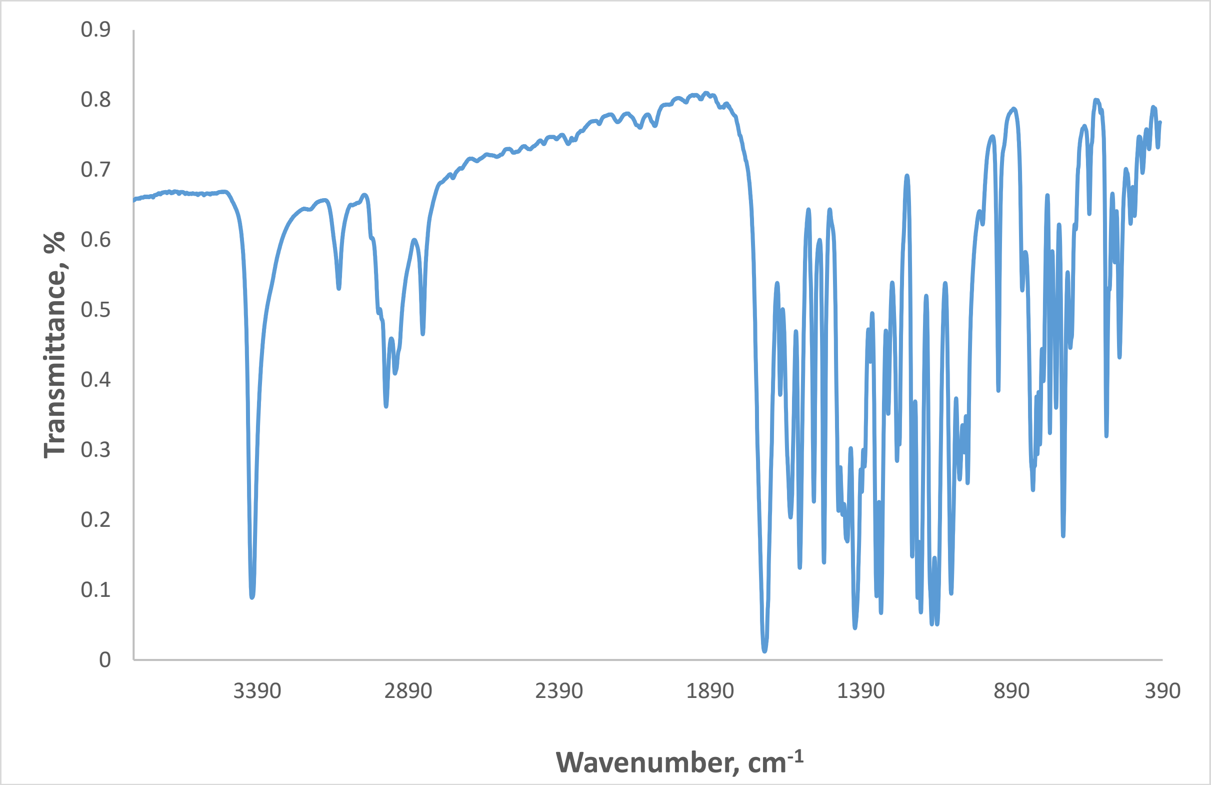

Figure S37 FTIR spectrum of compound **4i**, KBr tablet, cm^{-1}

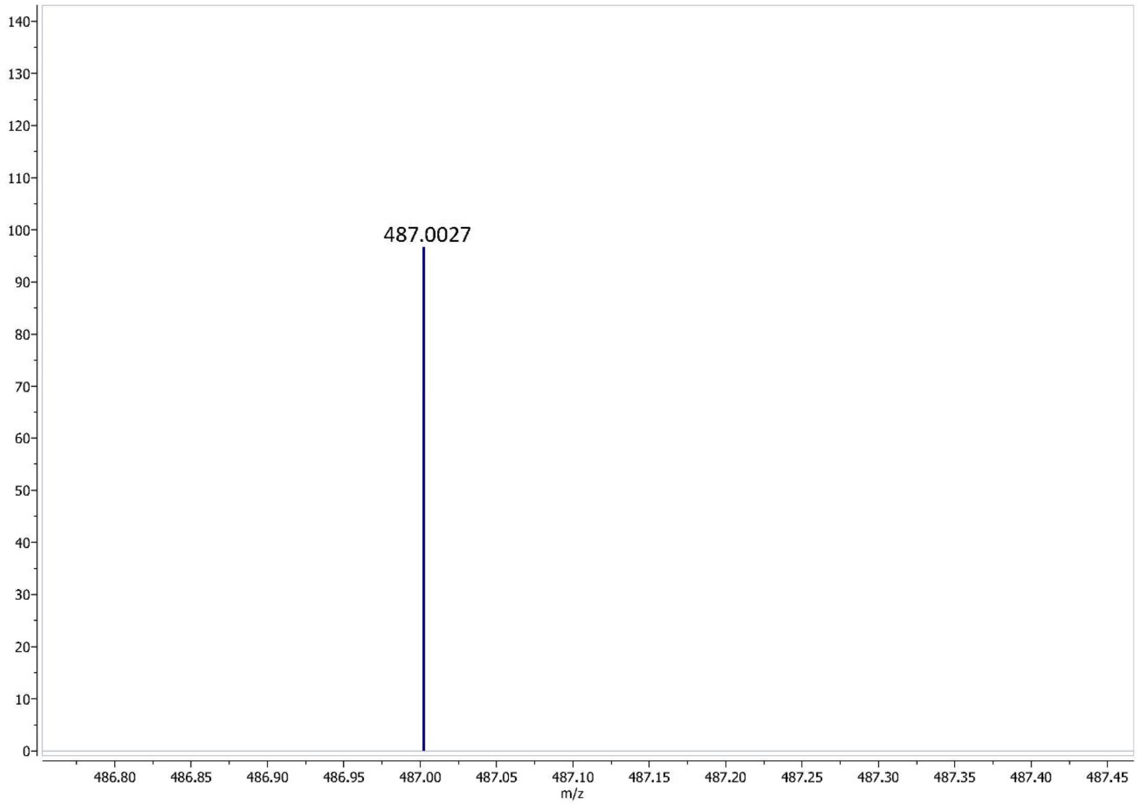

Figure S38 ESI-HRMS spectrum of compound **4i**, positive mode

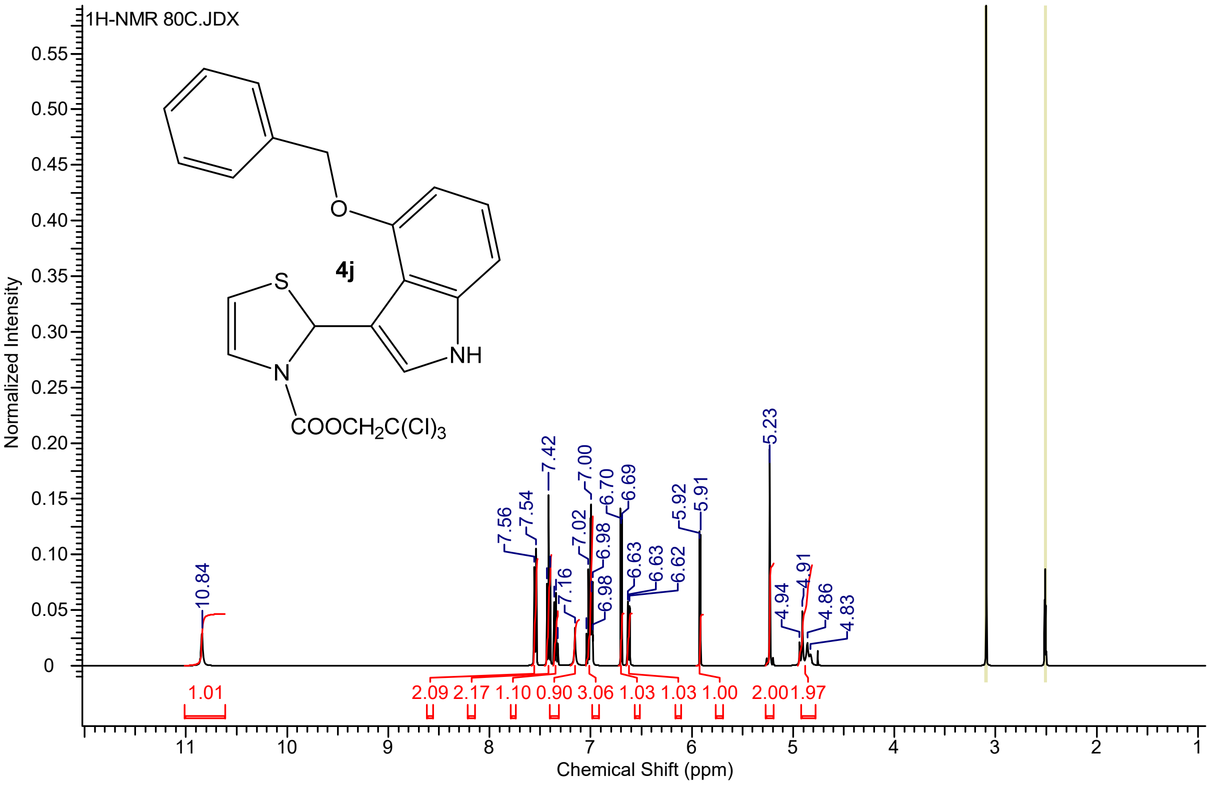

Figure S39 ^{1}H-NMR spectrum of **4j** - DMSO-d$_6$, 80 ^{o}C, 400 MHz

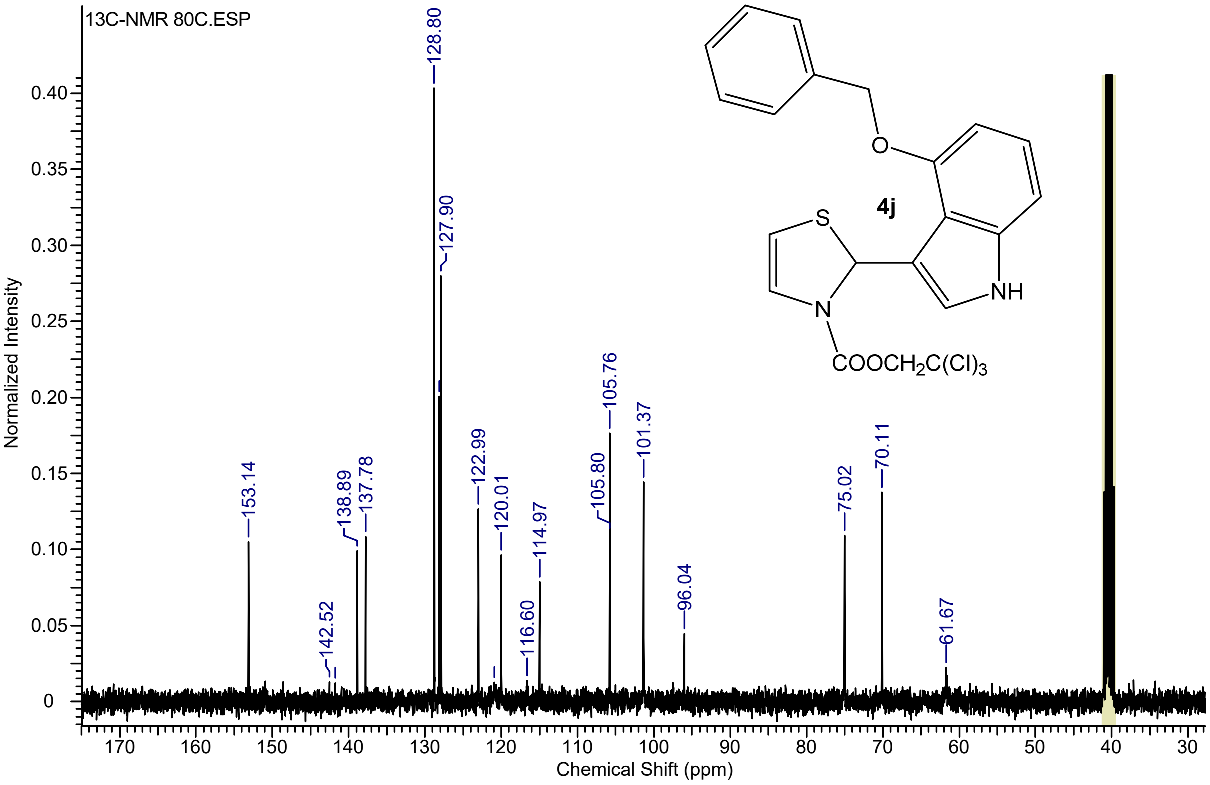

Figure S40 ^{13}C{^{1}H}-NMR spectrum of **4j** - DMSO-d$_6$, 80 ^{o}C, 100 MHz

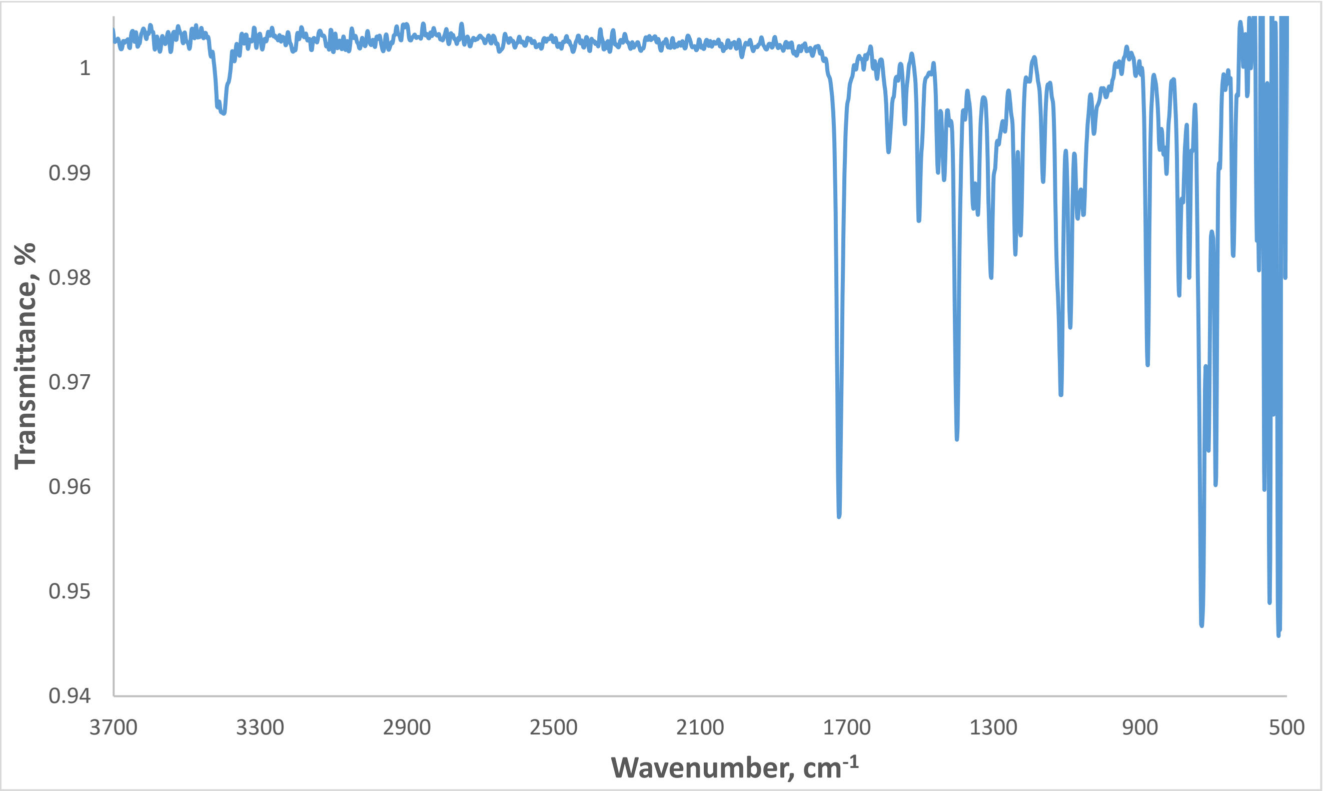

Figure S41 ATR-FTIR spectrum of compound **4j**, cm^{-1}

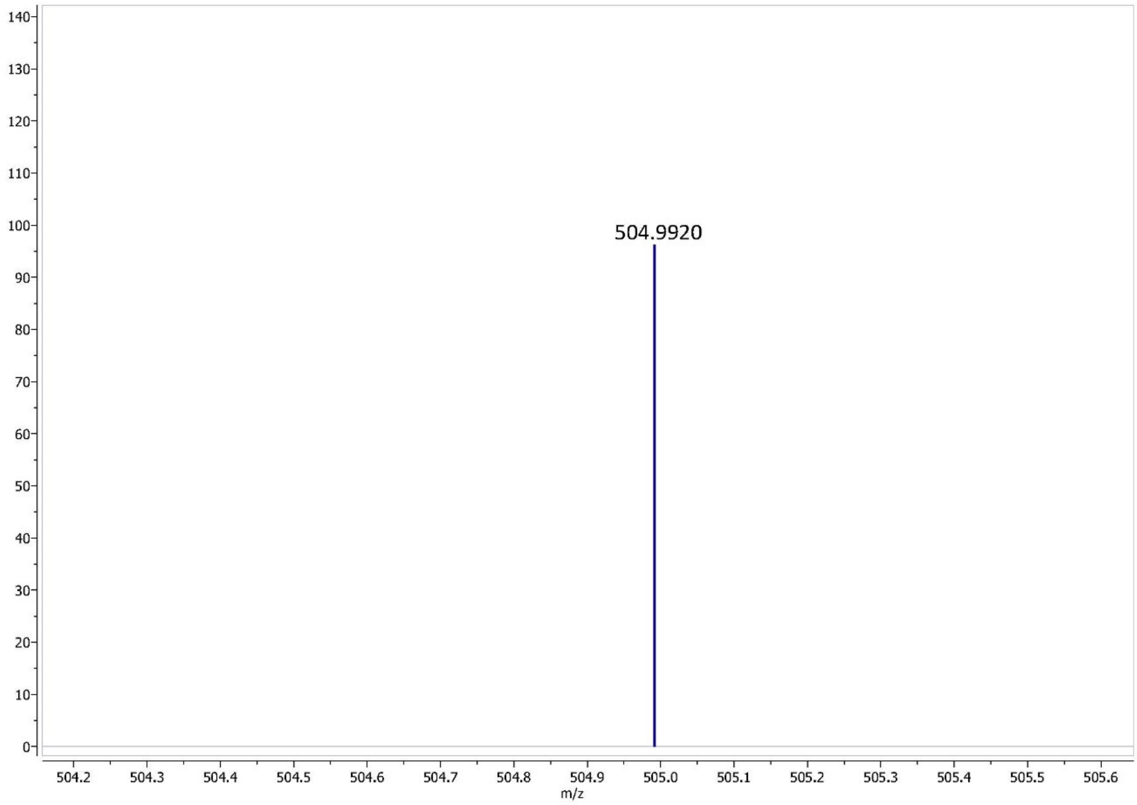

Figure S42 ESI-HRMS spectrum of compound **4j**, positive mode

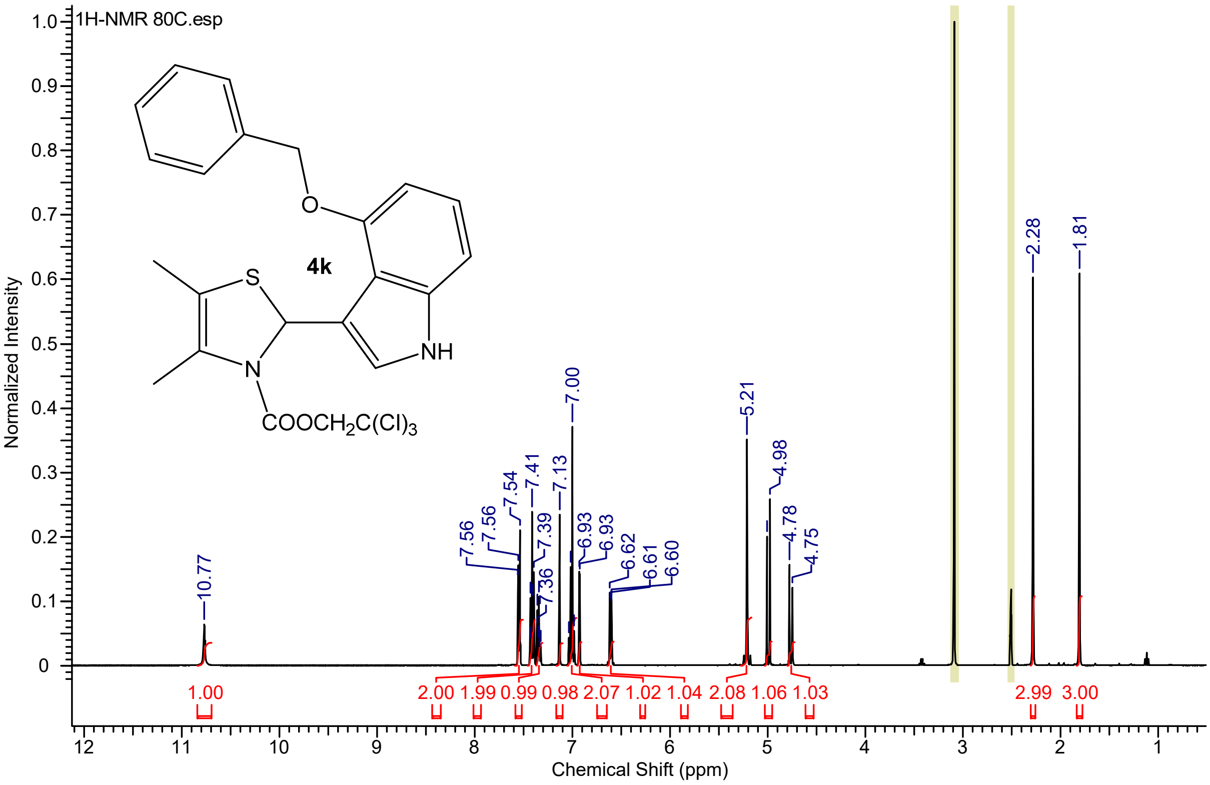

Figure S43 ^{1}H-NMR spectrum of **4k** - DMSO-d$_6$, 80 °C, 400 MHz

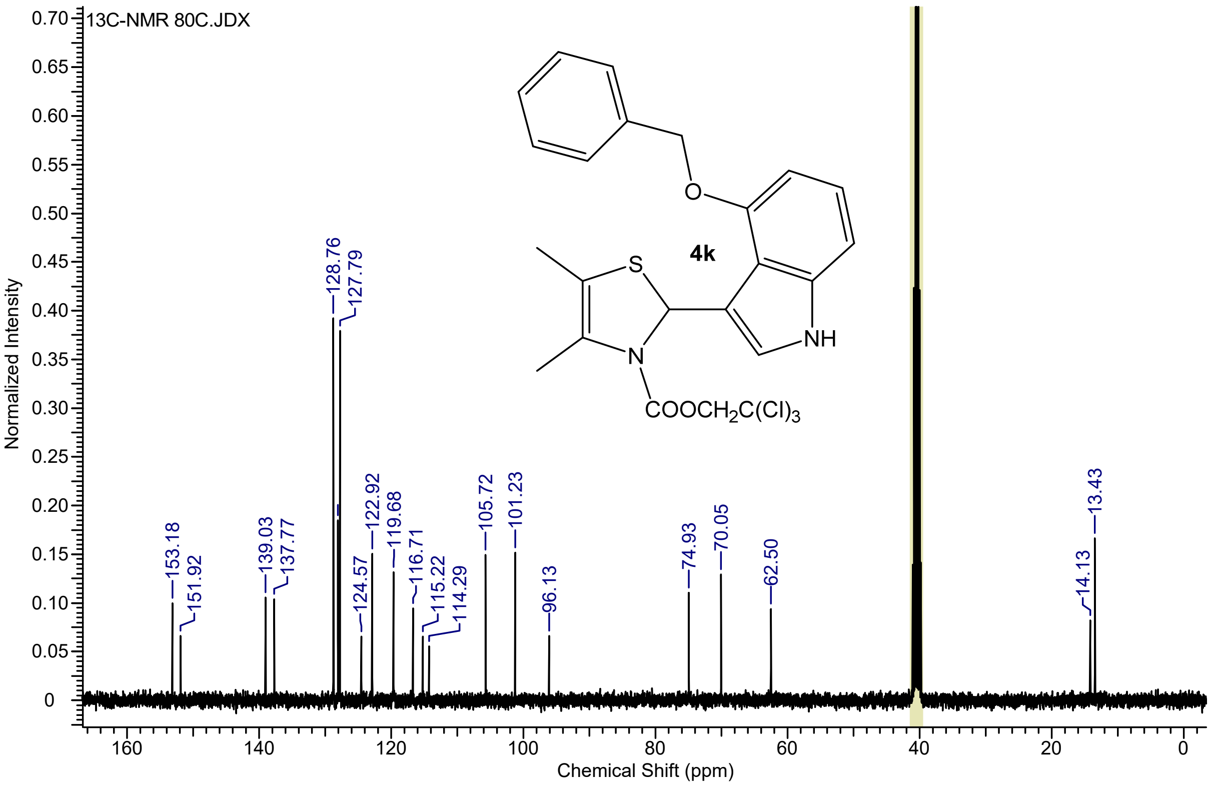

Figure S44 ^{13}C{^{1}H}-NMR spectrum of **4k** - DMSO-d$_6$, 80 °C, 100 MHz

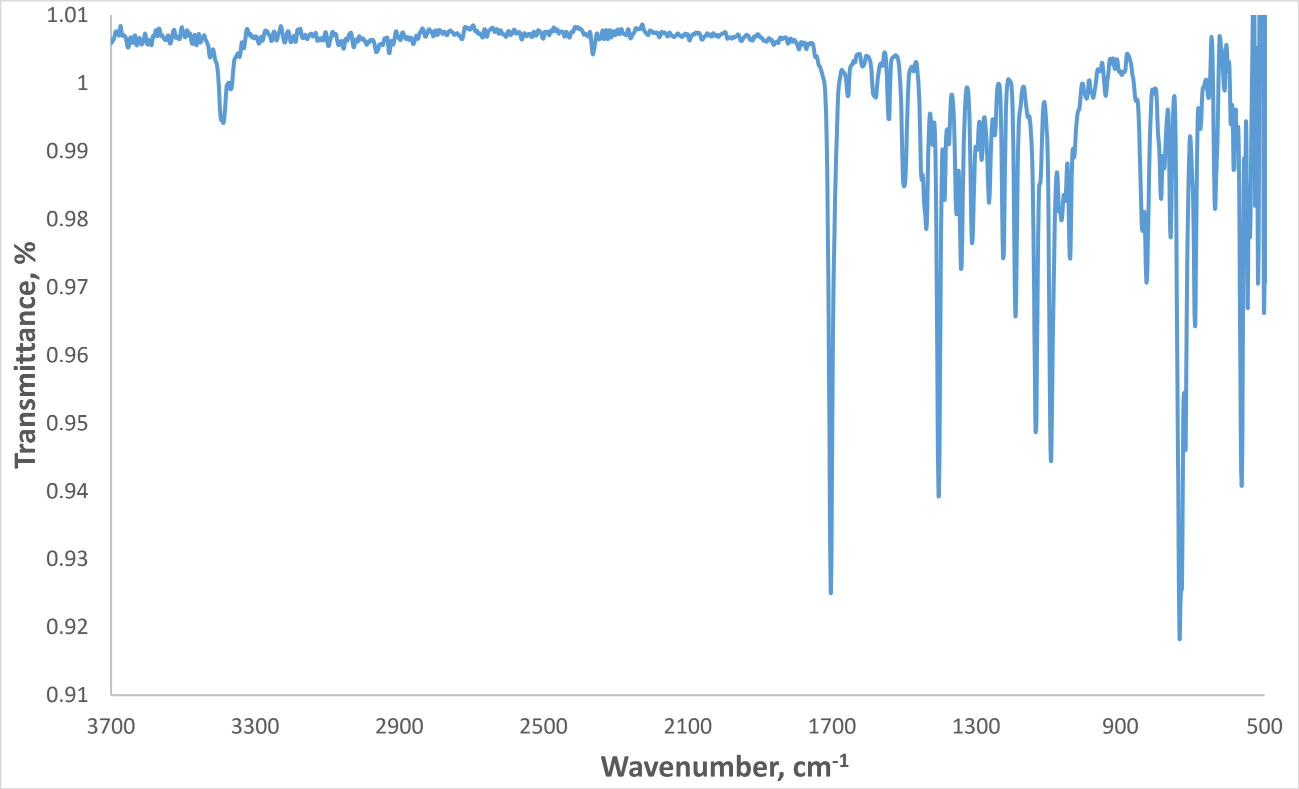

Figure S45 ATR-FTIR spectrum of compound **4k**, cm^{-1}

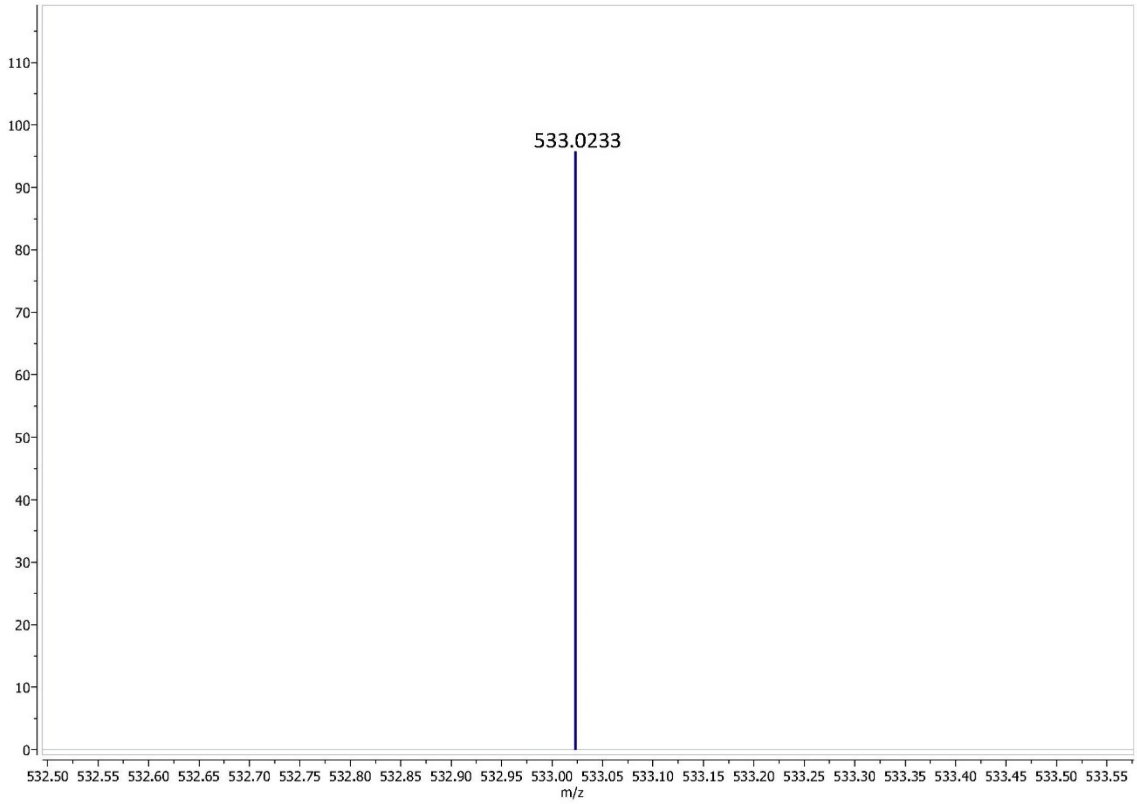

Figure S46 ESI-HRMS spectrum of compound **4k**, positive mode

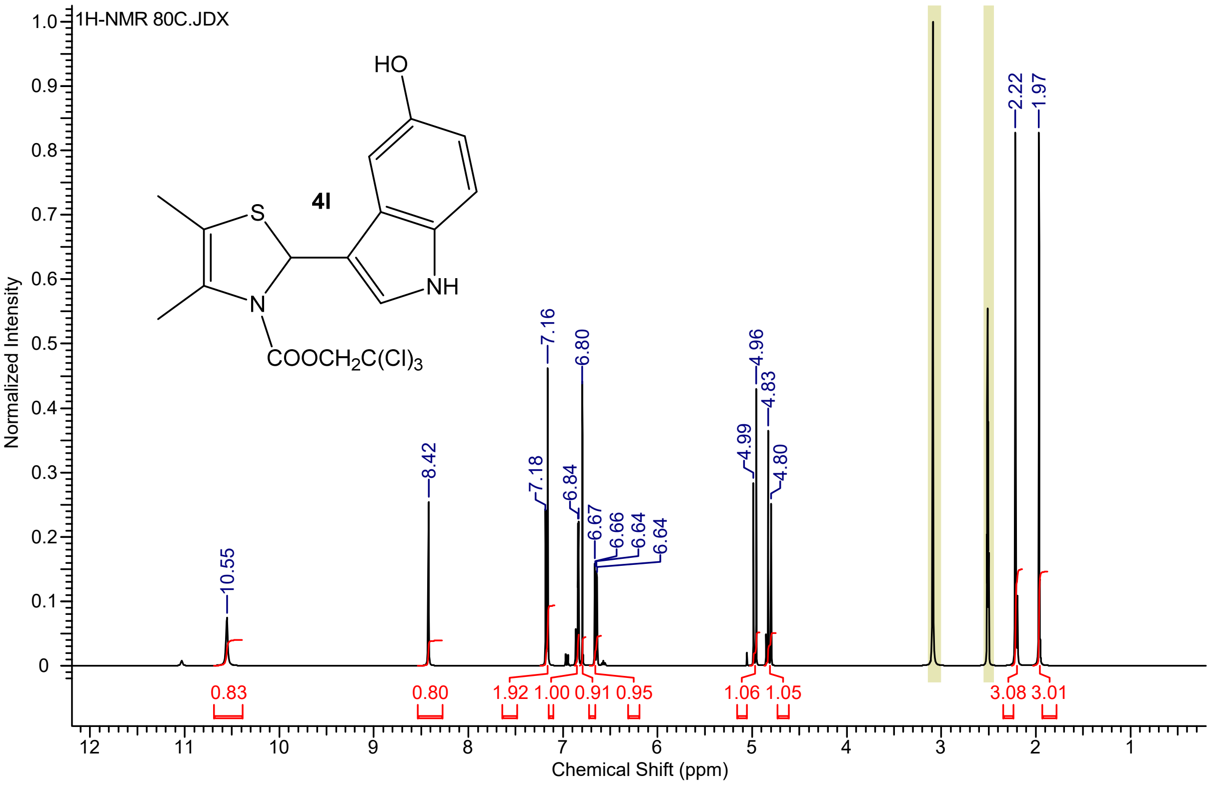

Figure S47 ^{1}H-NMR spectrum of **4l** - DMSO-d$_6$, 80 °C, 400 MHz

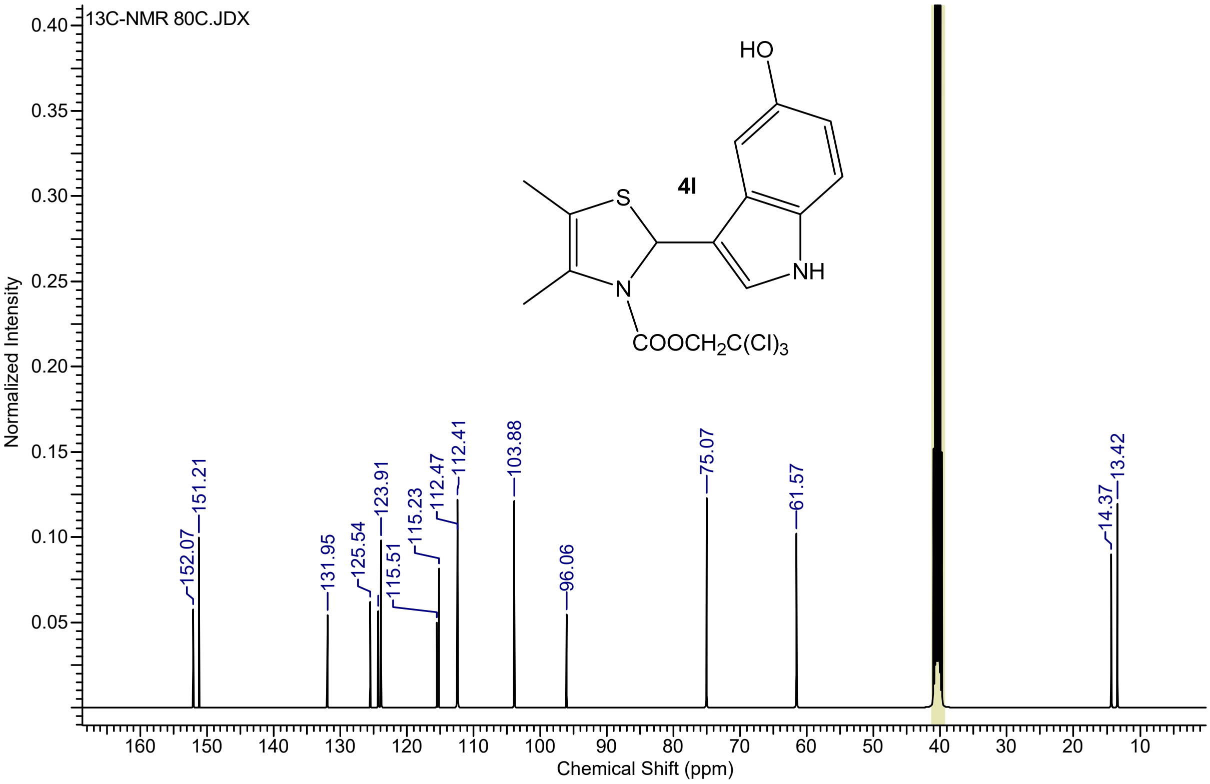

Figure S48 $^{13}C\{^{1}H\}$-NMR spectrum of **4l** - DMSO-d$_6$, 80 °C, 100 MHz

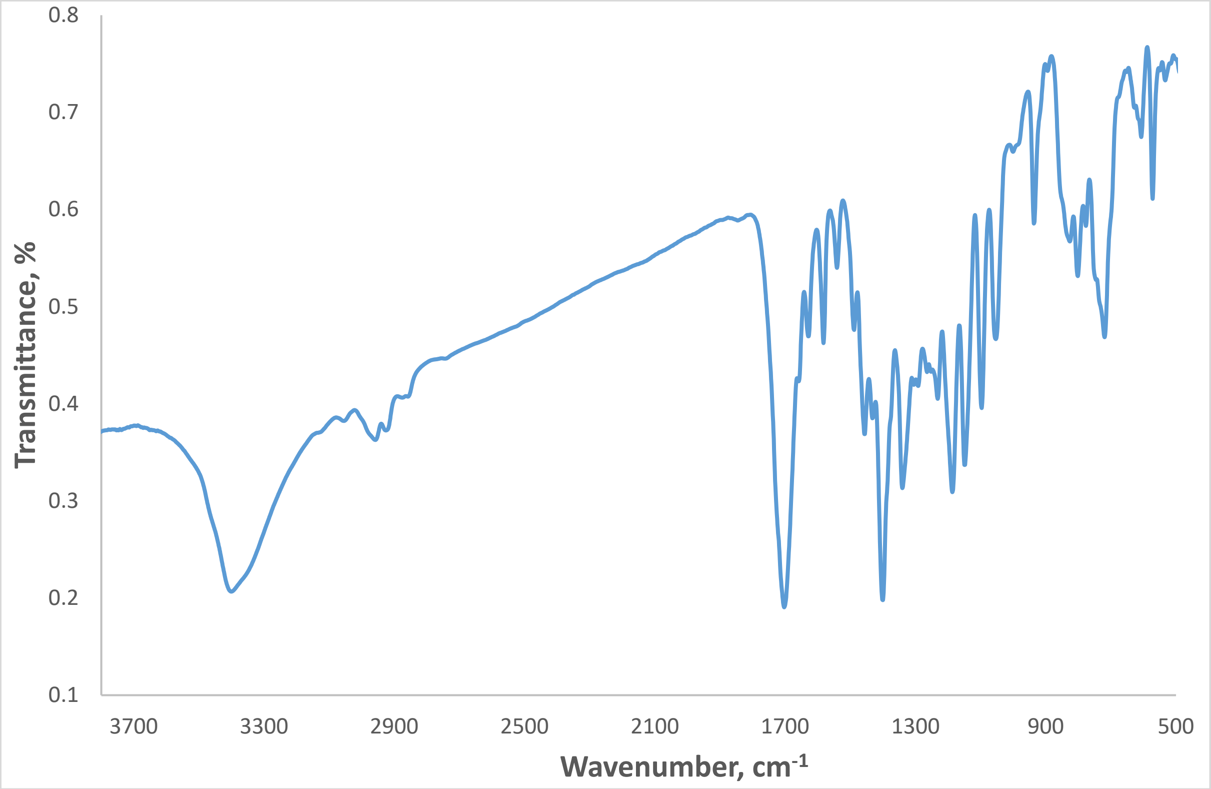

Figure S49 FTIR spectrum of compound **4l**, KBr tablet, cm^{-1}

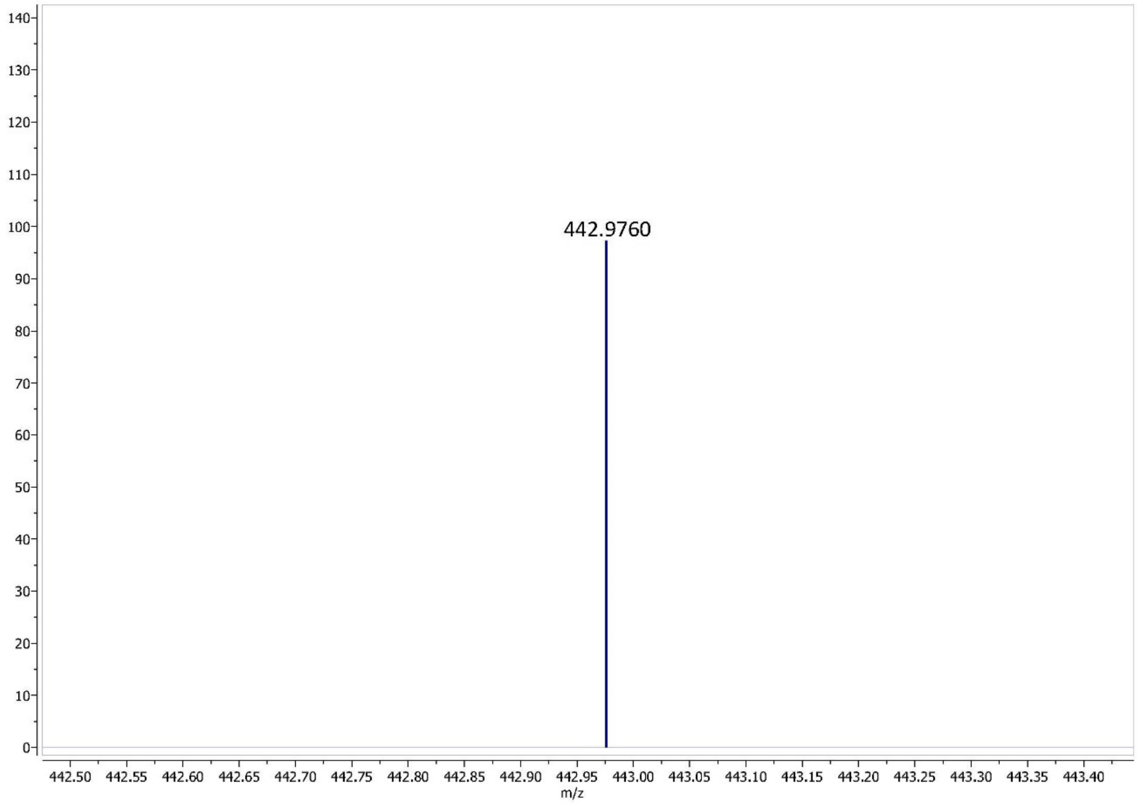

Figure S50 ESI-HRMS spectrum of compound **4l**, positive mode

Spectral data of oxy-camalexins:

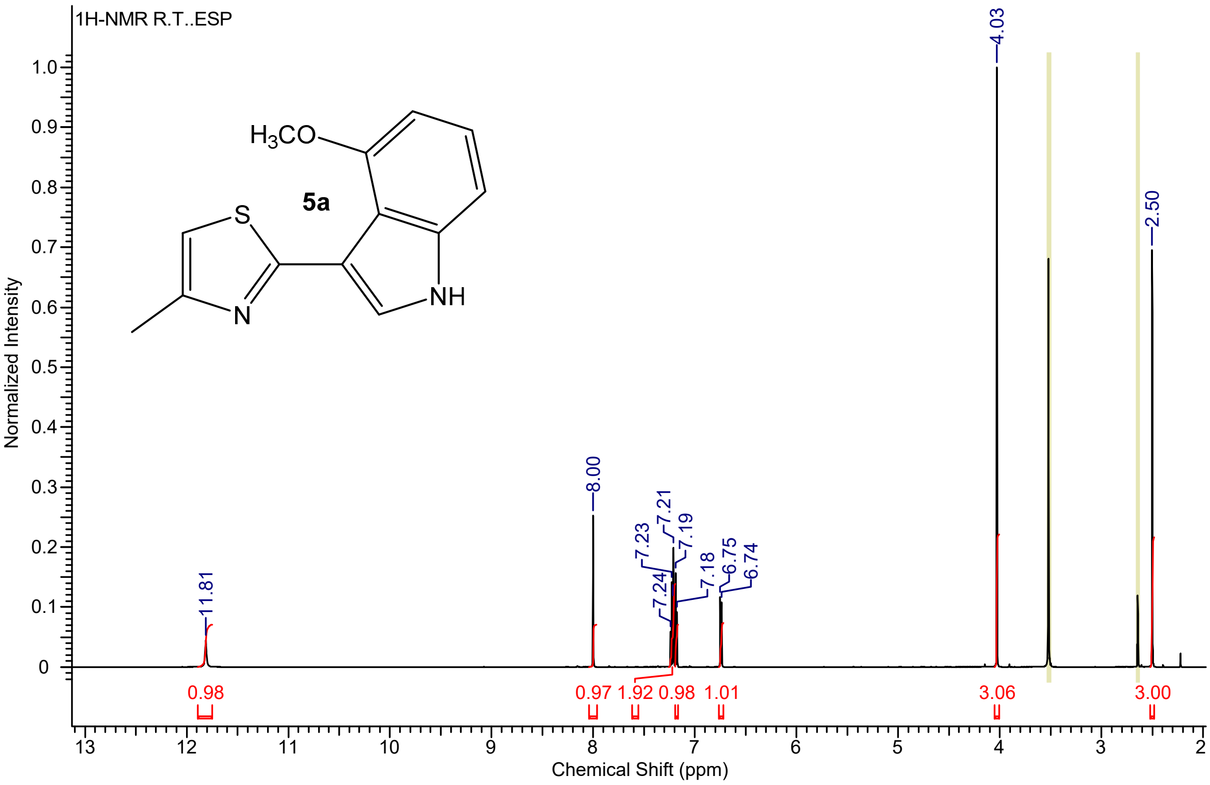

Figure S51 ^{1}H-NMR spectrum of **5a** - DMSO-d$_6$, r.t., 600 MHz

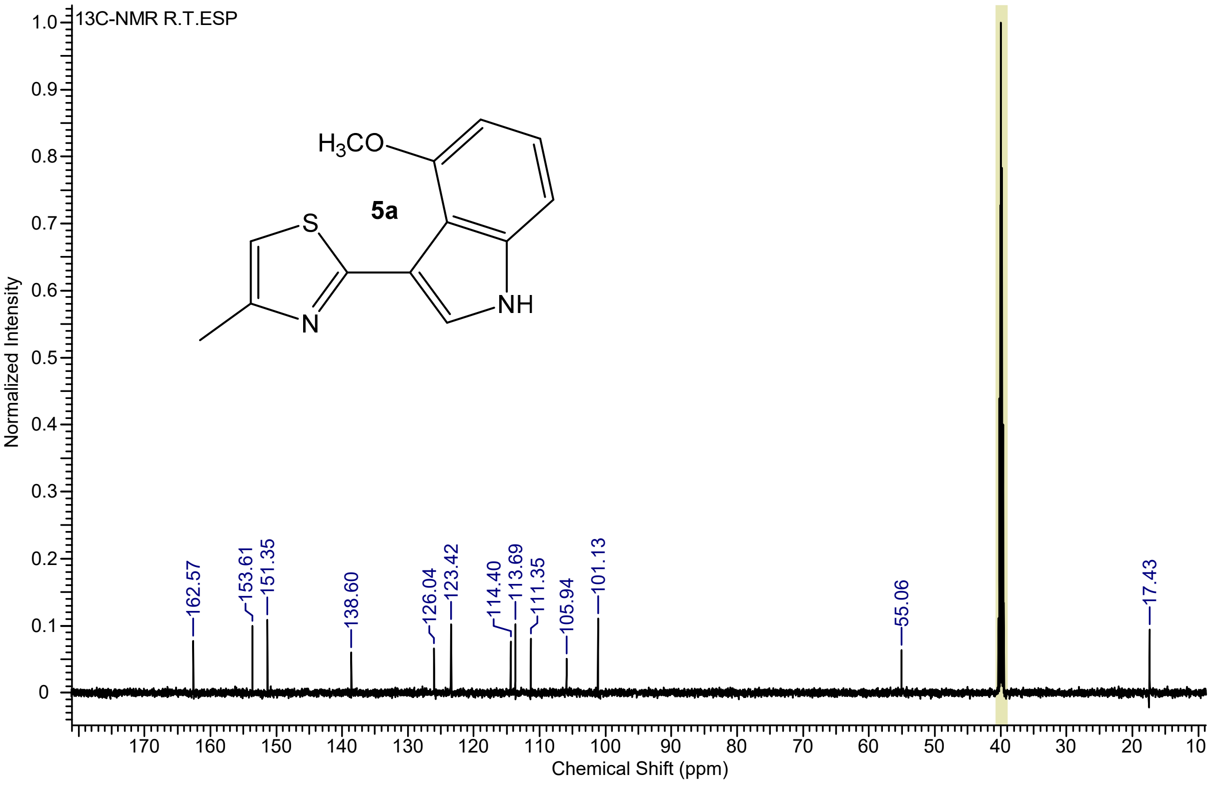

Figure S52 ^{13}C{^{1}H}-NMR spectrum of **5a** - DMSO-d$_6$, r.t., 150 MHz

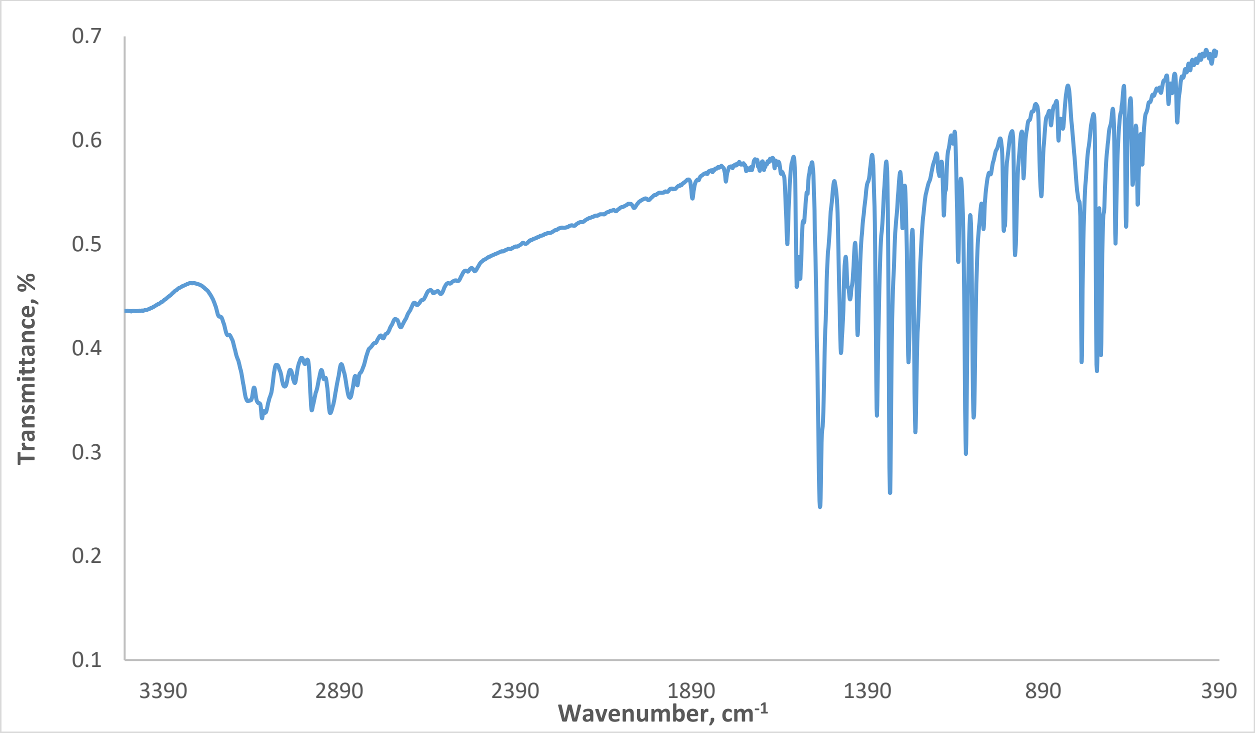

Figure S53 FTIR spectrum of compound **5a**, KBr tablet, cm^{-1}

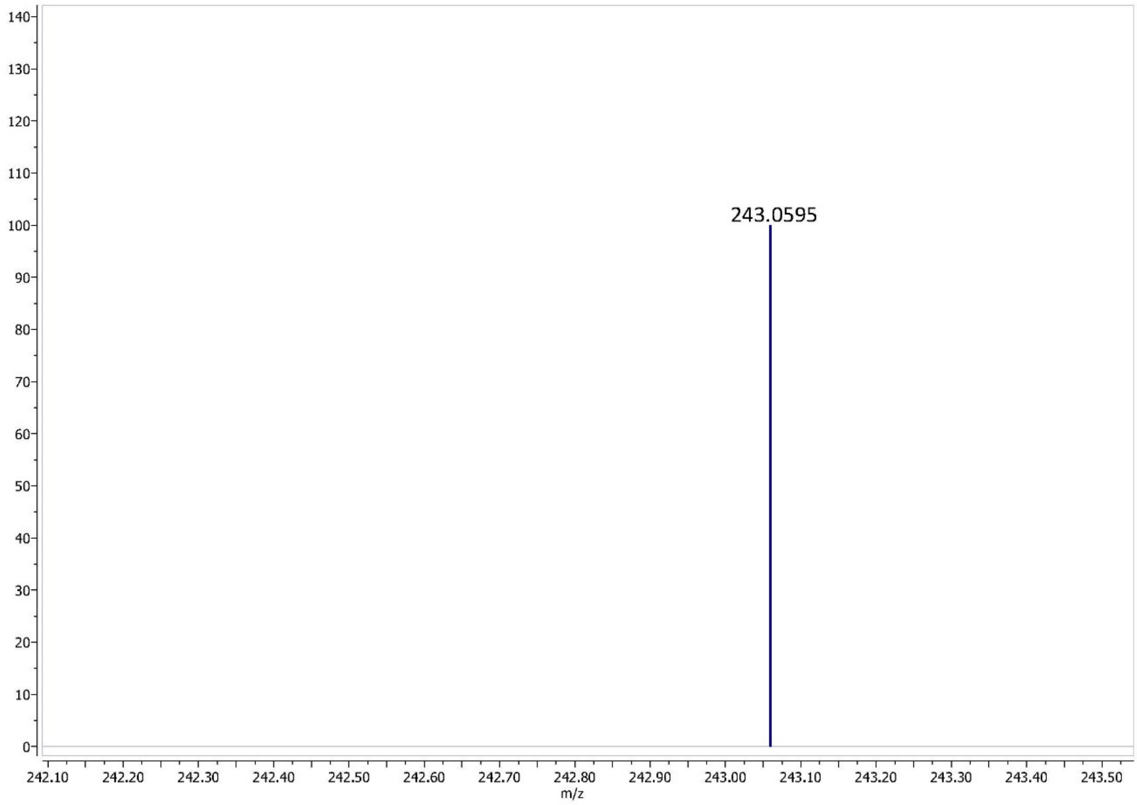

Figure S54 ESI-HRMS spectrum of compound **5a**, negative mode

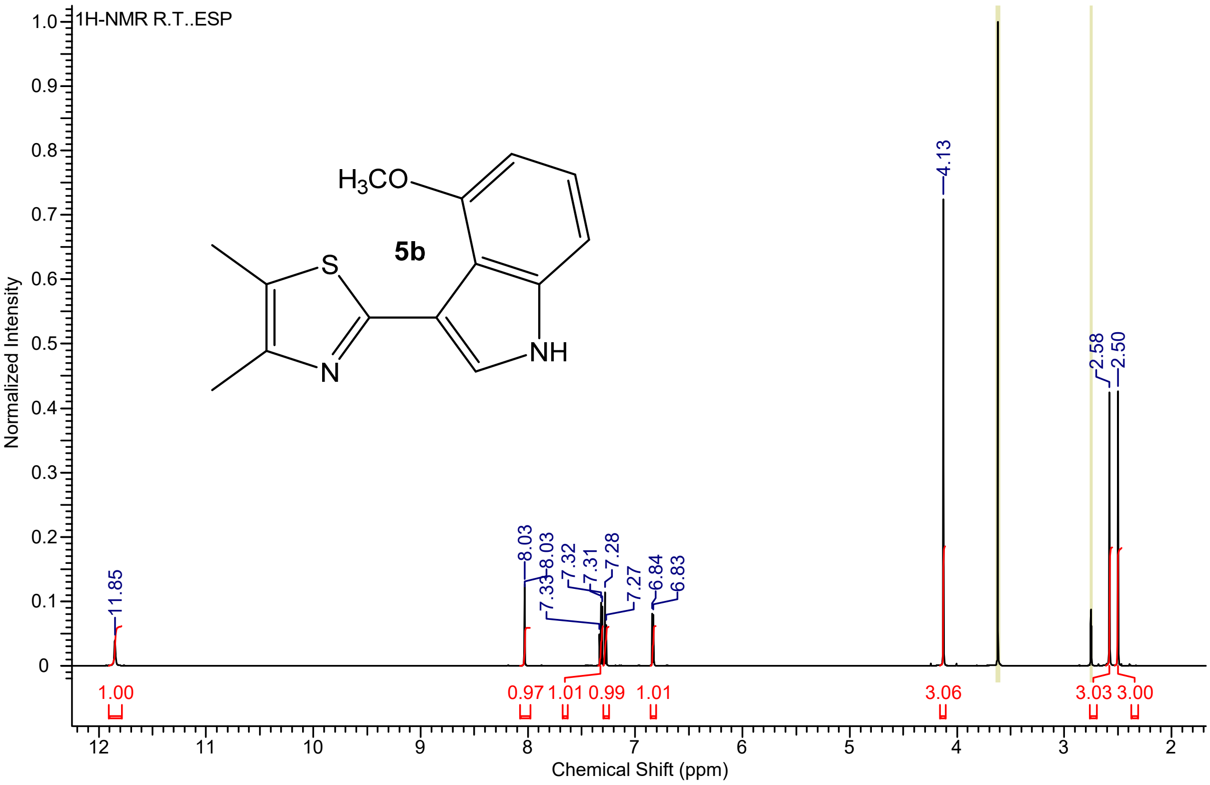

Figure S55 ^{1}H-NMR spectrum of **5b** - DMSO-d6, r.t., 600 MHz

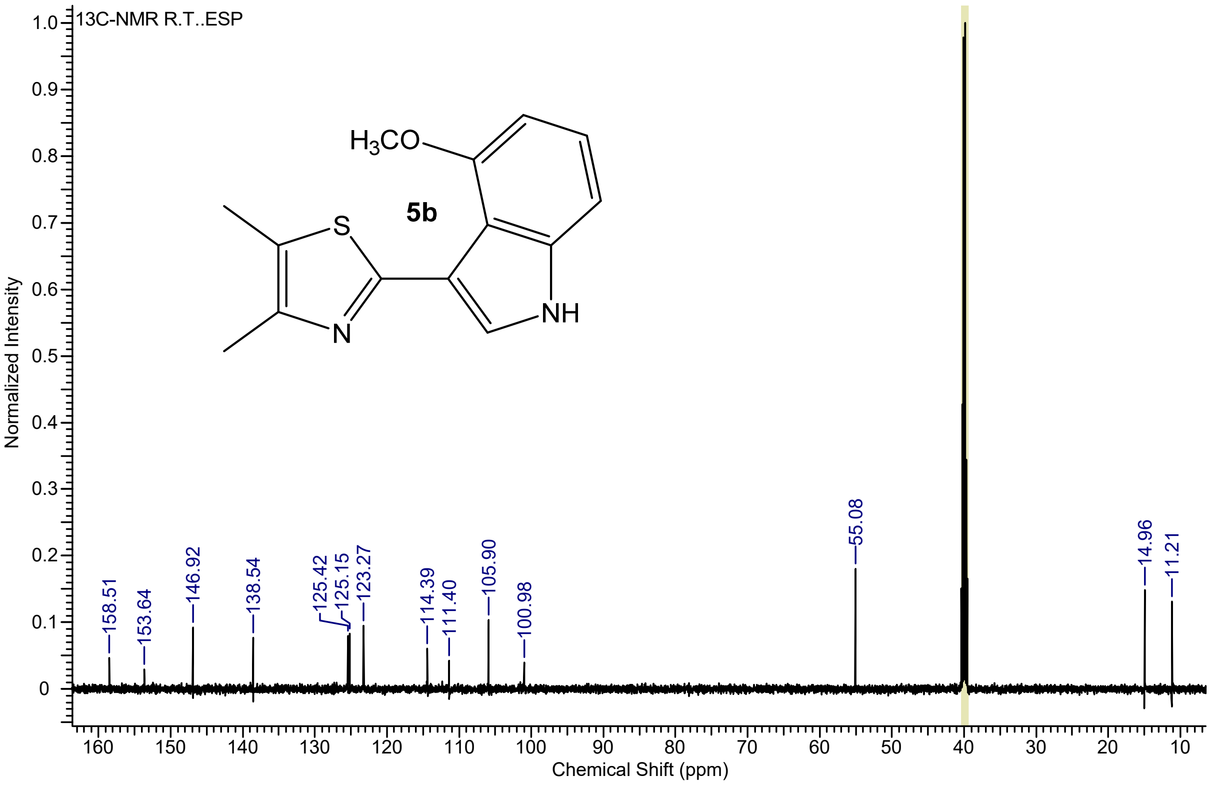

Figure S56 ^{13}C{^{1}H}-NMR spectrum of **5b** - DMSO-d6, r.t., 150 MHz

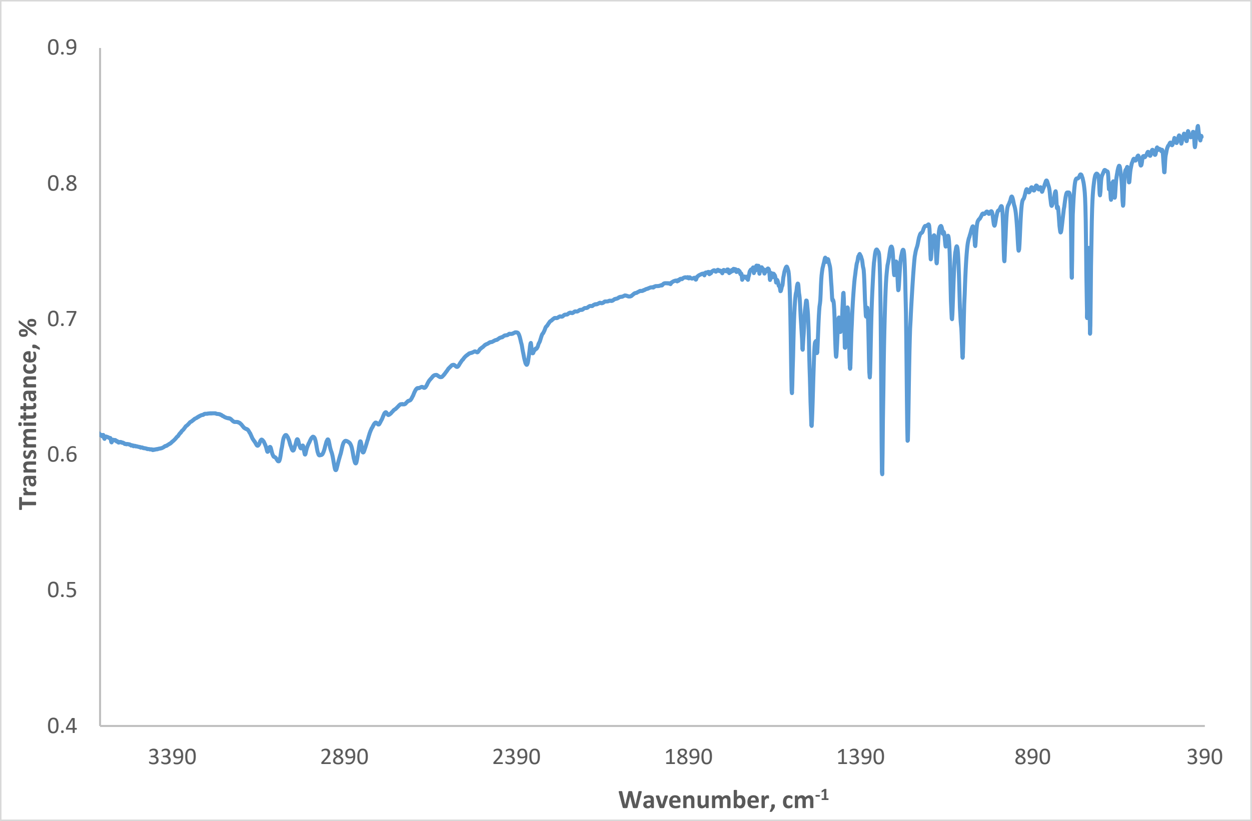

Figure S57 FTIR spectrum of compound **5b**, KBr tablet, cm^{-1}

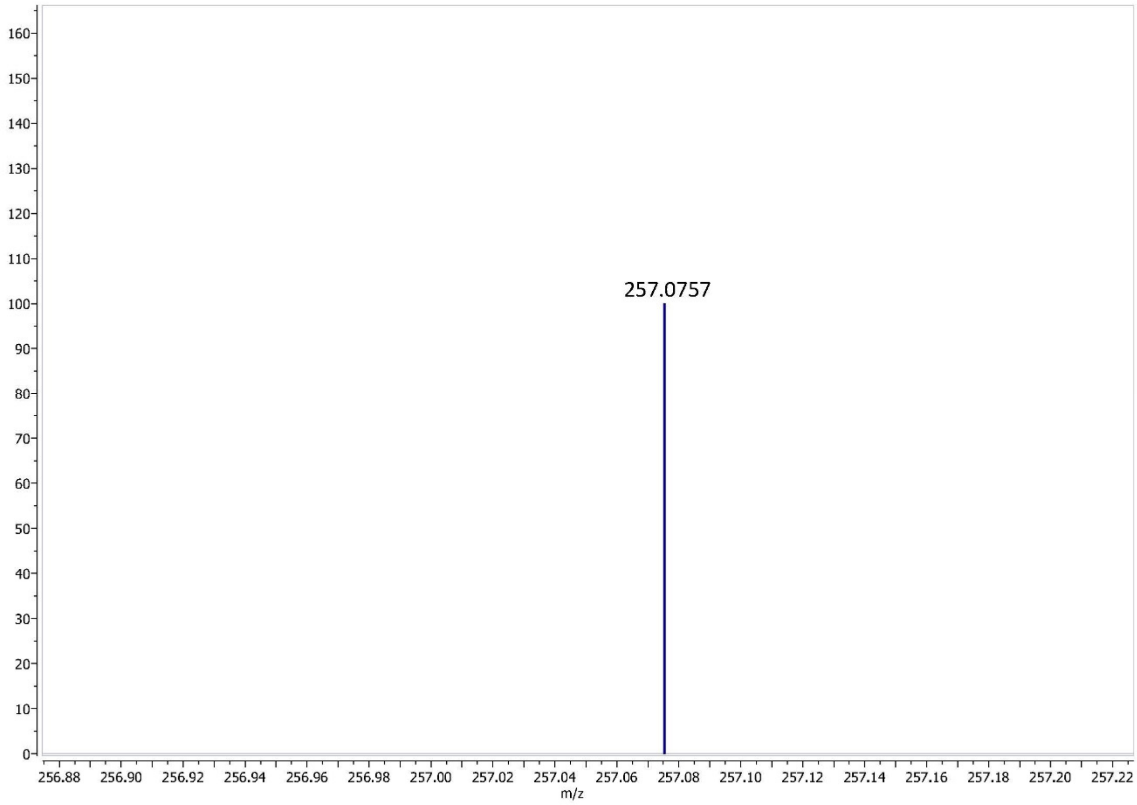

Figure S58 ESI-HRMS spectrum of compound **5b**, negative mode

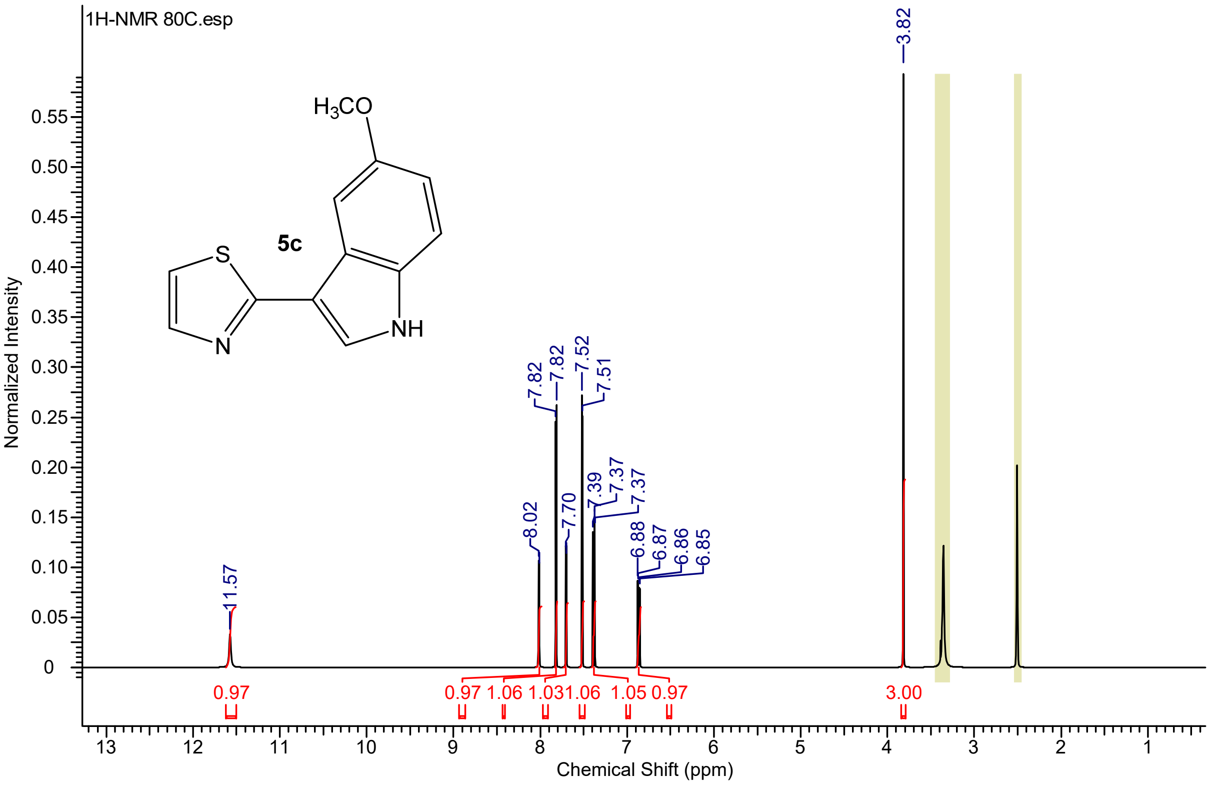

Figure S59 ^{1}H-NMR spectrum of **5c** - DMSO-d_6, r.t., 400 MHz

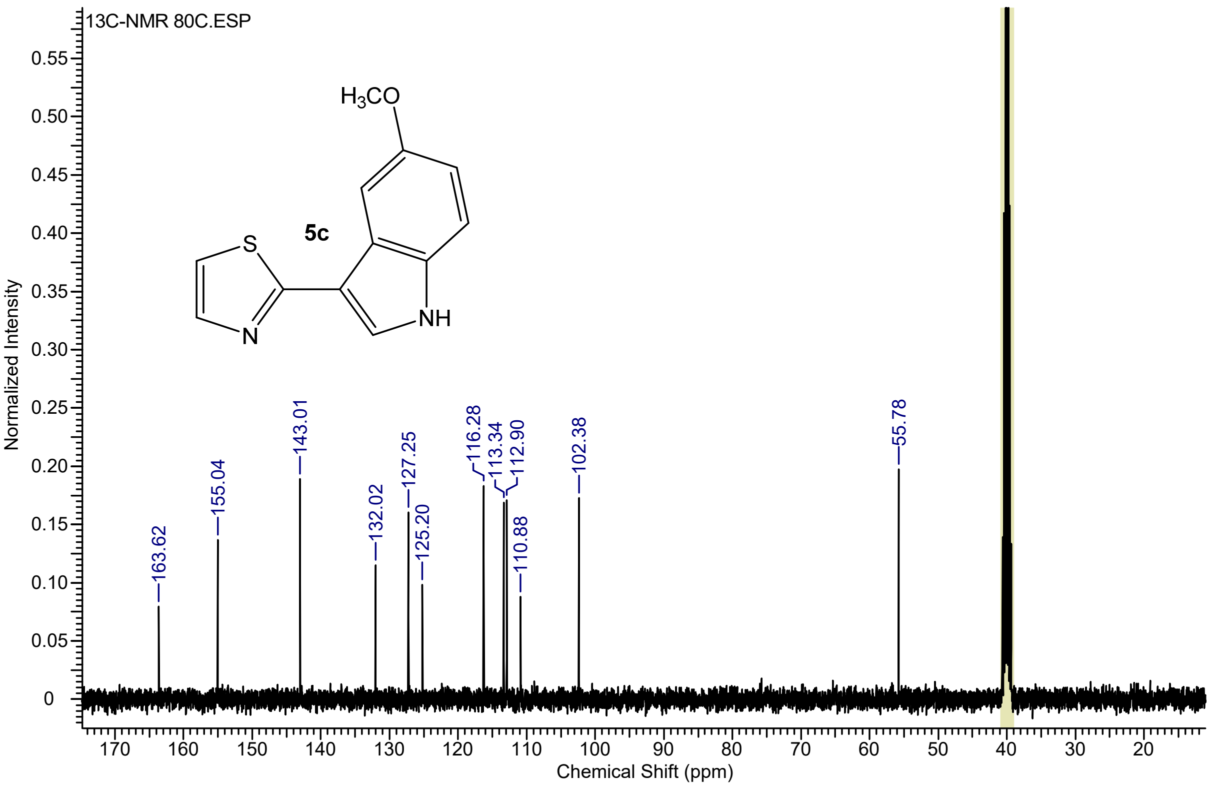

Figure S60 ^{13}C{^{1}H}-NMR spectrum of **5c** - DMSO-d_6, r.t., 100 MHz

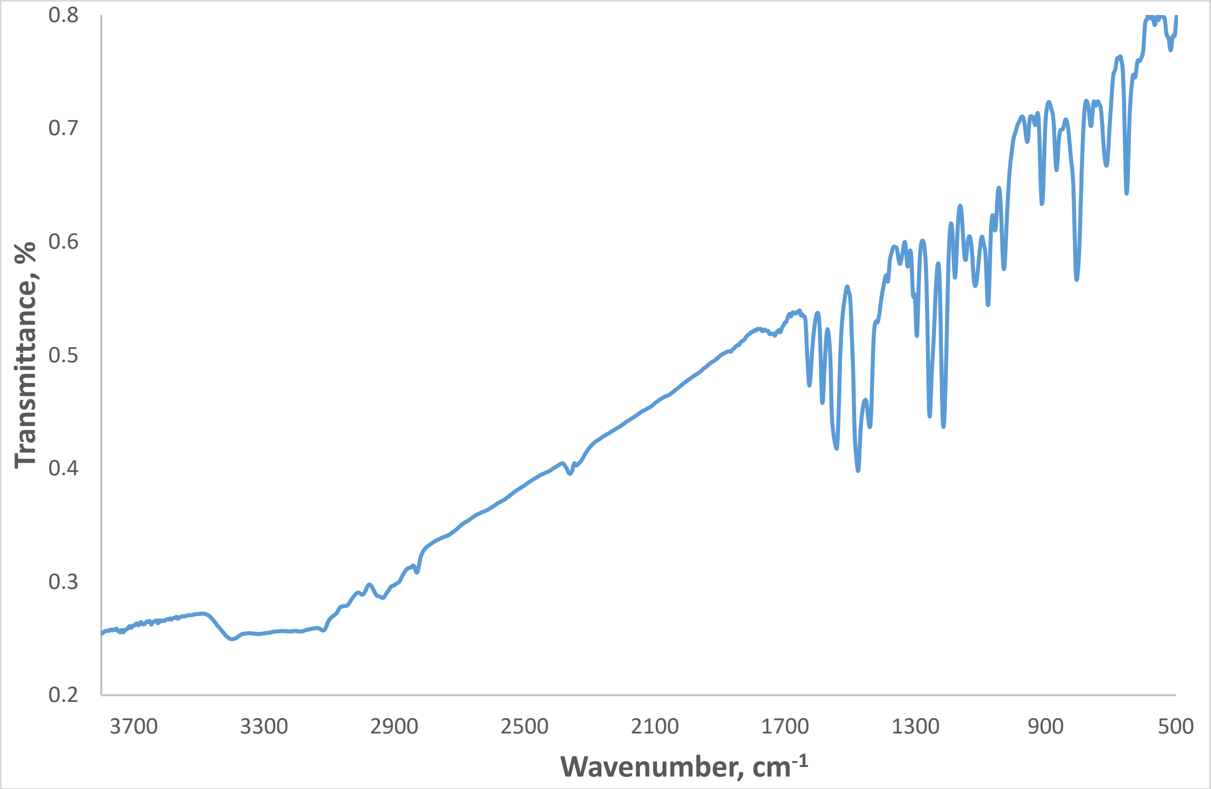

Figure S61 FTIR spectrum of compound **5c**, KBr tablet, cm^{-1}

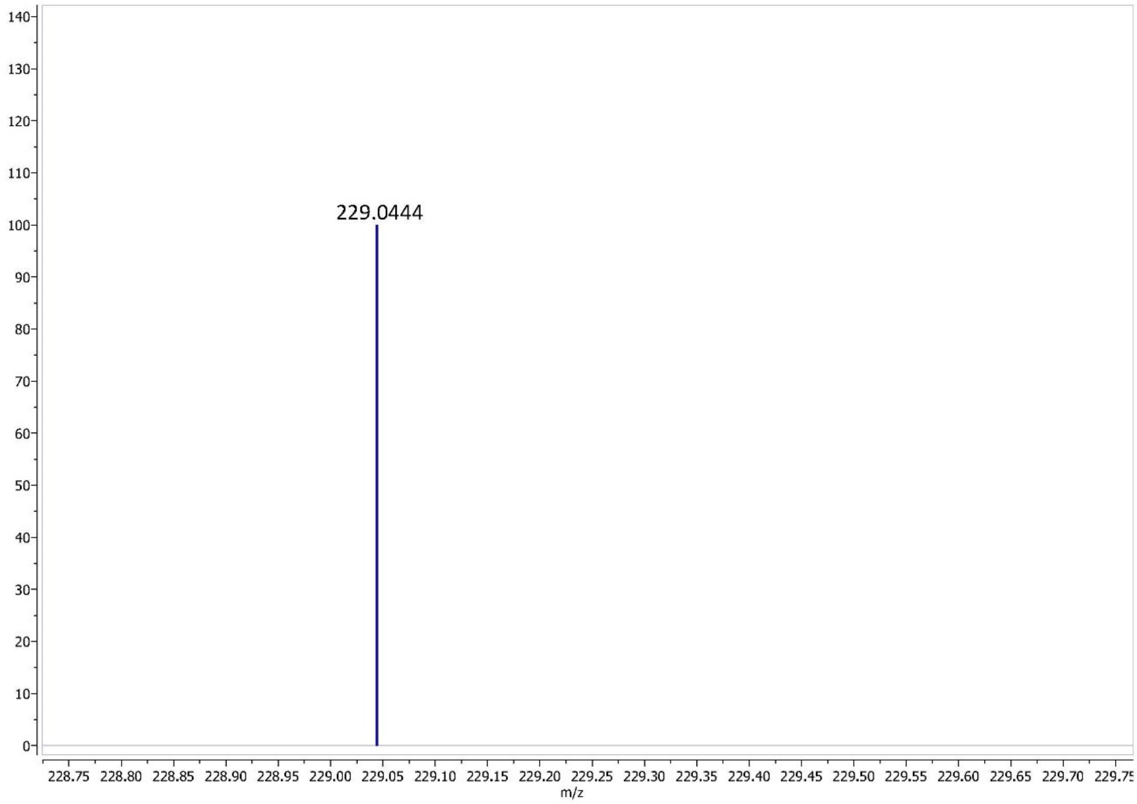

Figure S62 ESI-HRMS spectrum of compound **5c**, negative mode

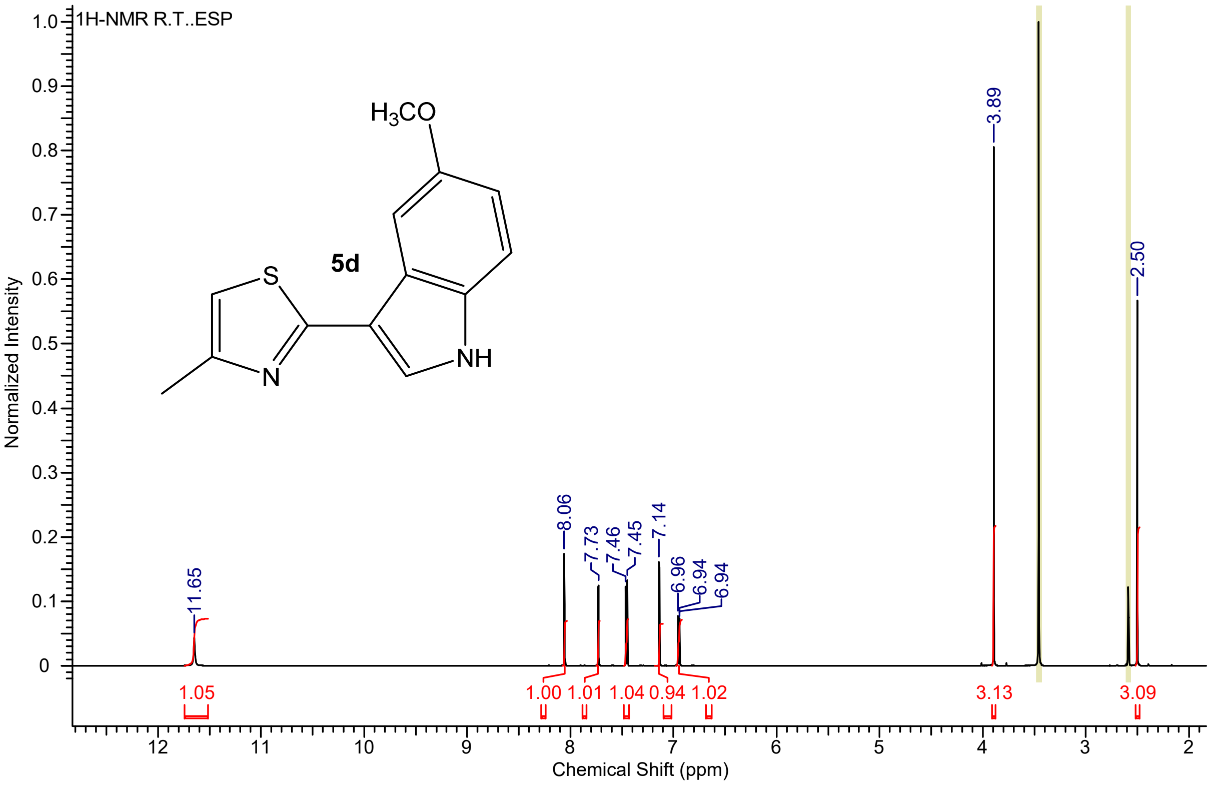

Figure S63 ^{1}H-NMR spectrum of **5d** - DMSO-d_6, r.t., 600 MHz

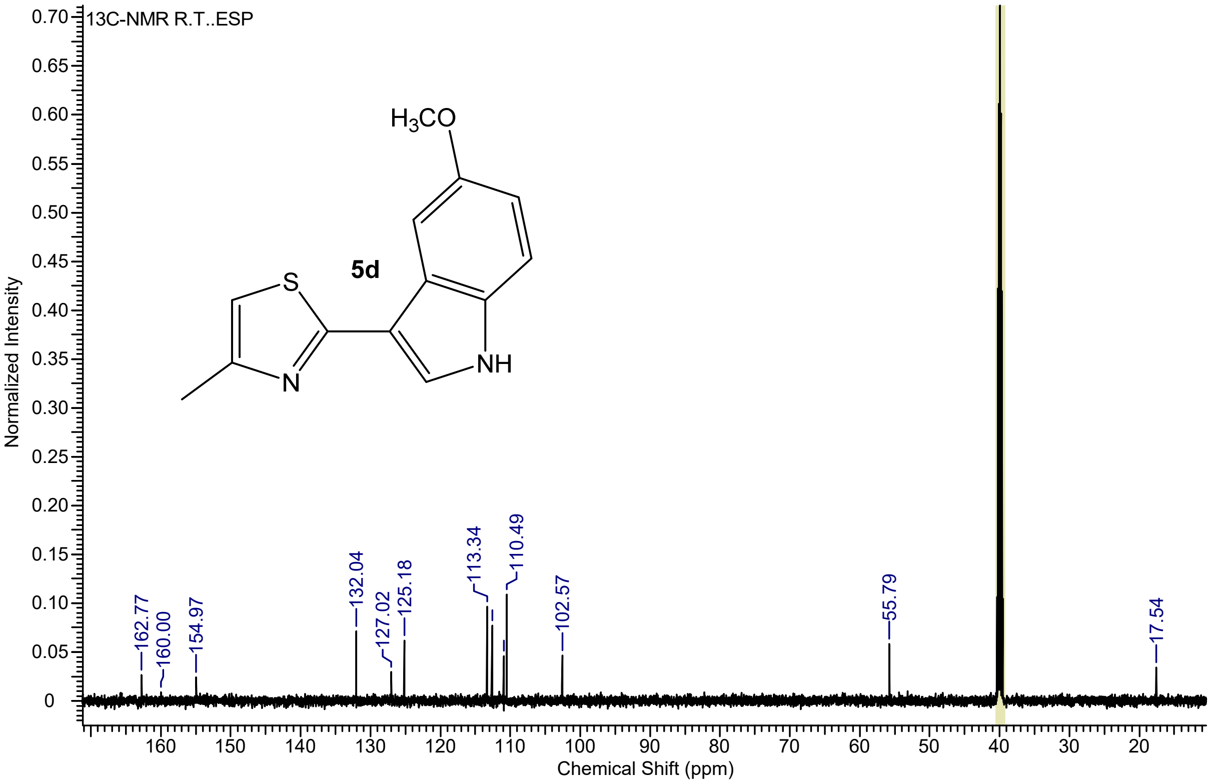

Figure S64 ^{13}C{^{1}H}-NMR spectrum of **5d** - DMSO-d_6, r.t., 150 MHz

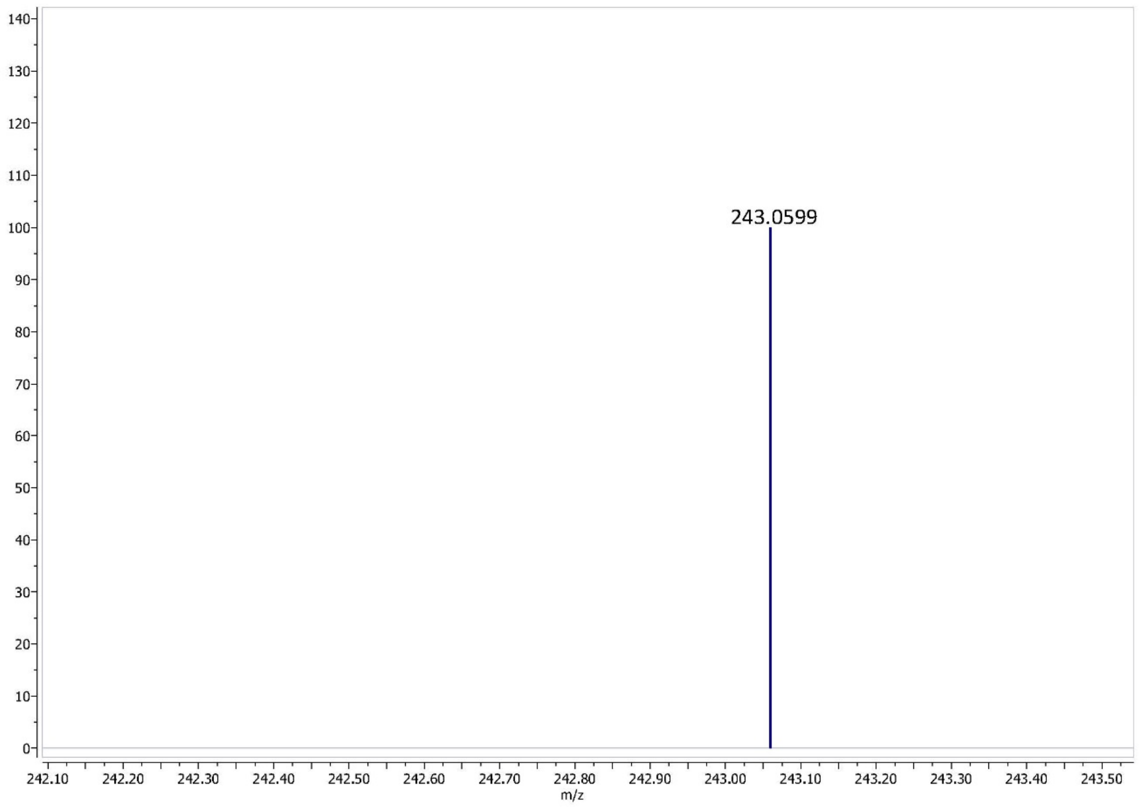

Figure S65 ESI-HRMS spectrum of compound **5d**, negative mode

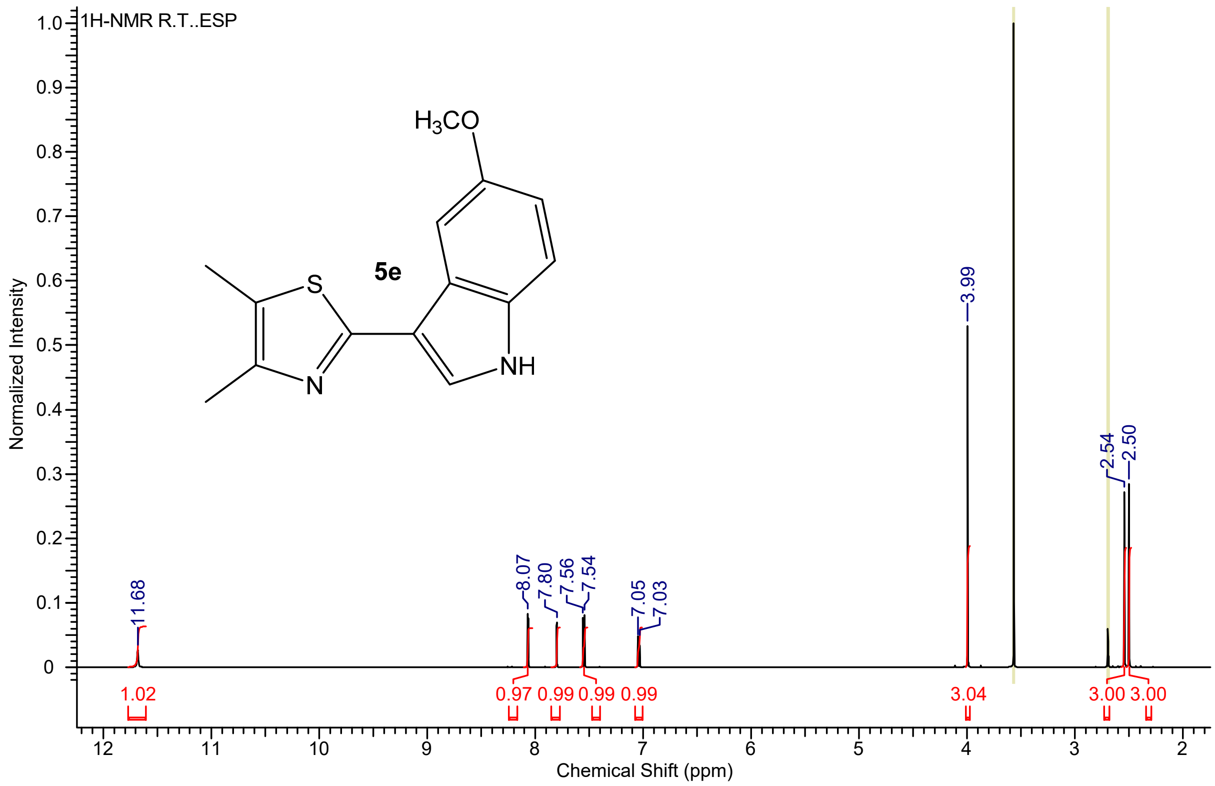

Figure S66 ^{1}H-NMR spectrum of **5e** - DMSO-d$_6$, r.t., 600 MHz

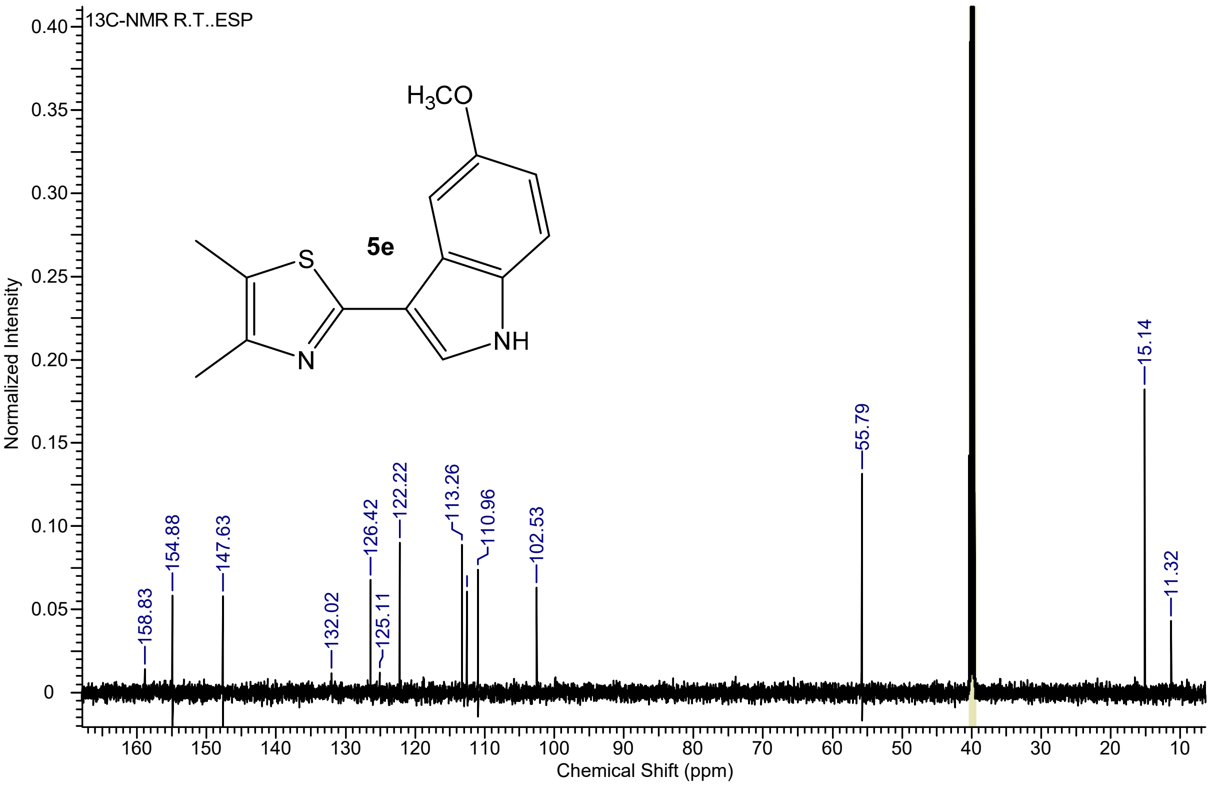

Figure S67 $^{13}C\{^1H\}$-NMR spectrum of **5e** - DMSO-d_6, r.t., 150 MHz

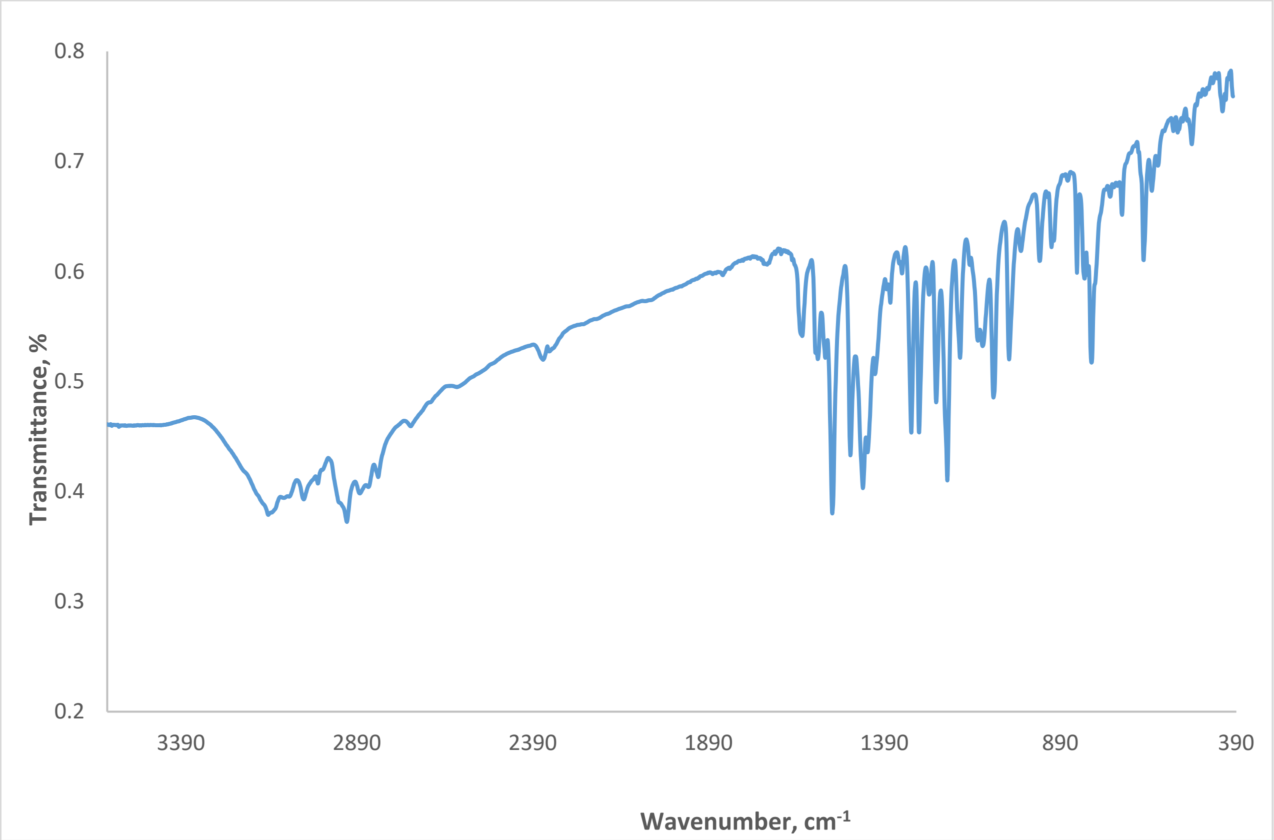

Figure S68 FTIR spectrum of compound **5e**, KBr tablet, cm^{-1}

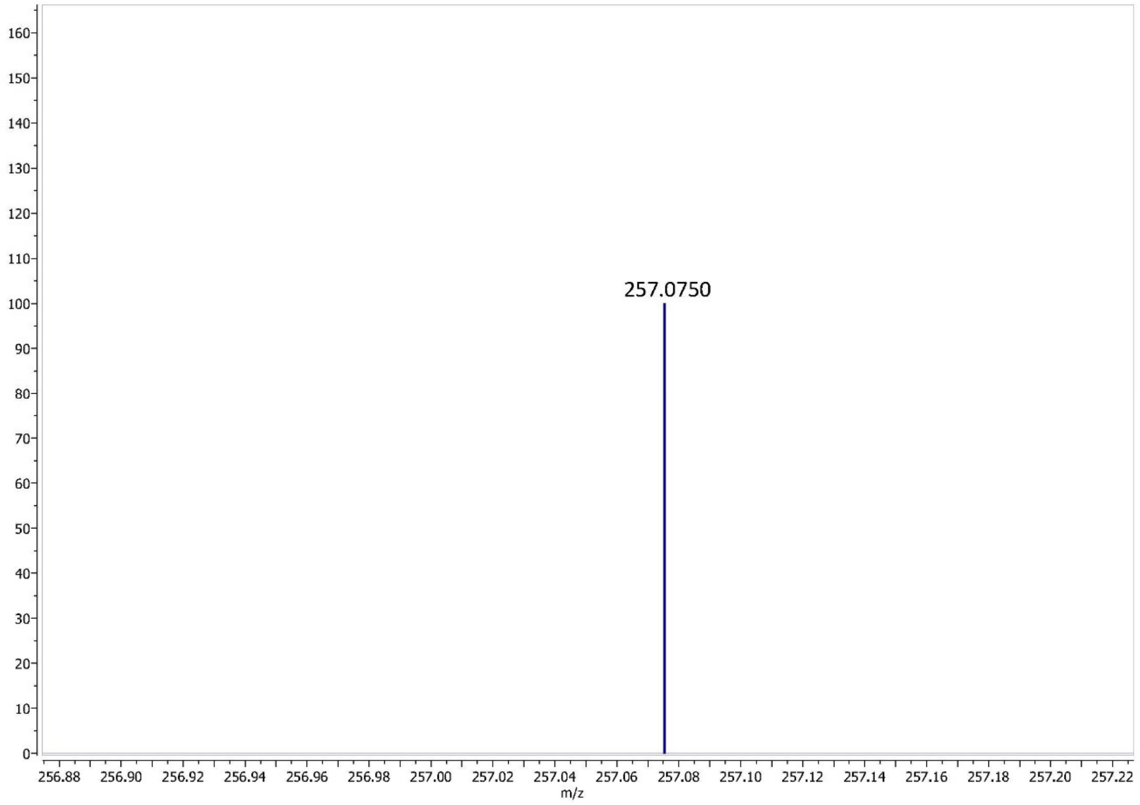

Figure S69 ESI-HRMS spectrum of compound **5e**, negative mode

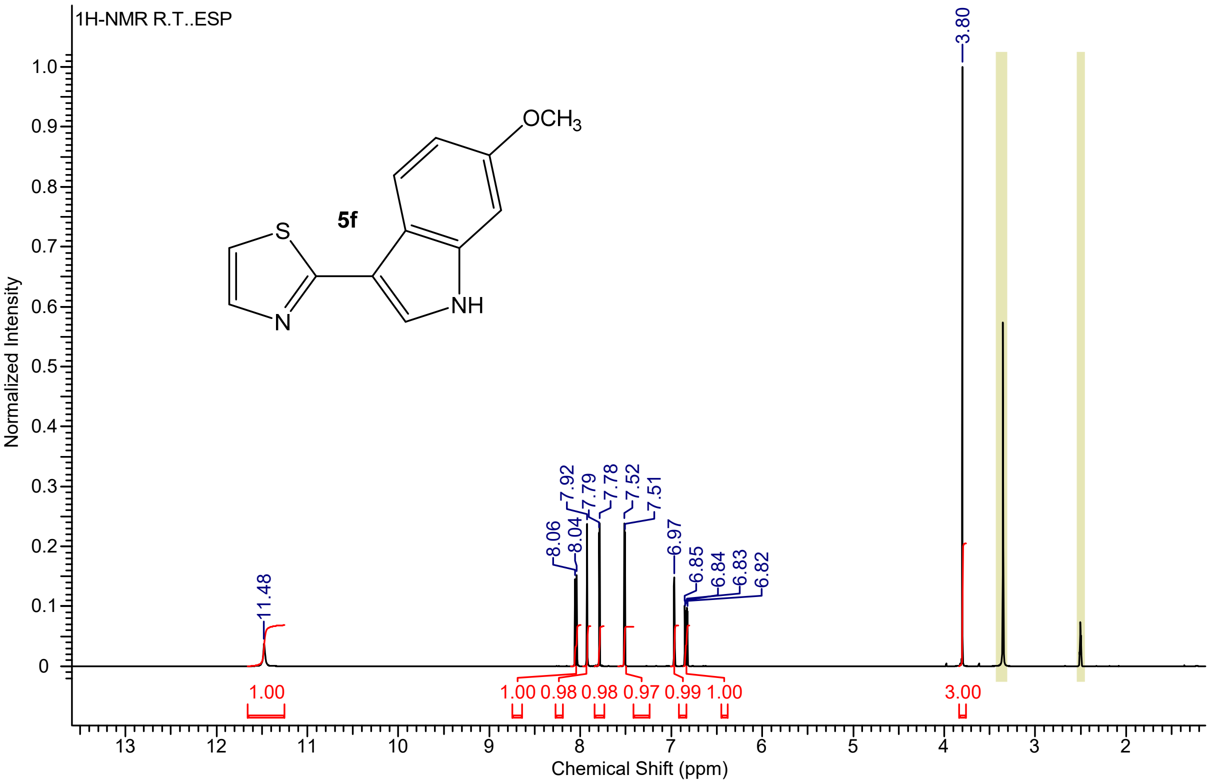

Figure S70 ^{1}H-NMR spectrum of **5f** - DMSO-d$_6$, r.t., 400 MHz

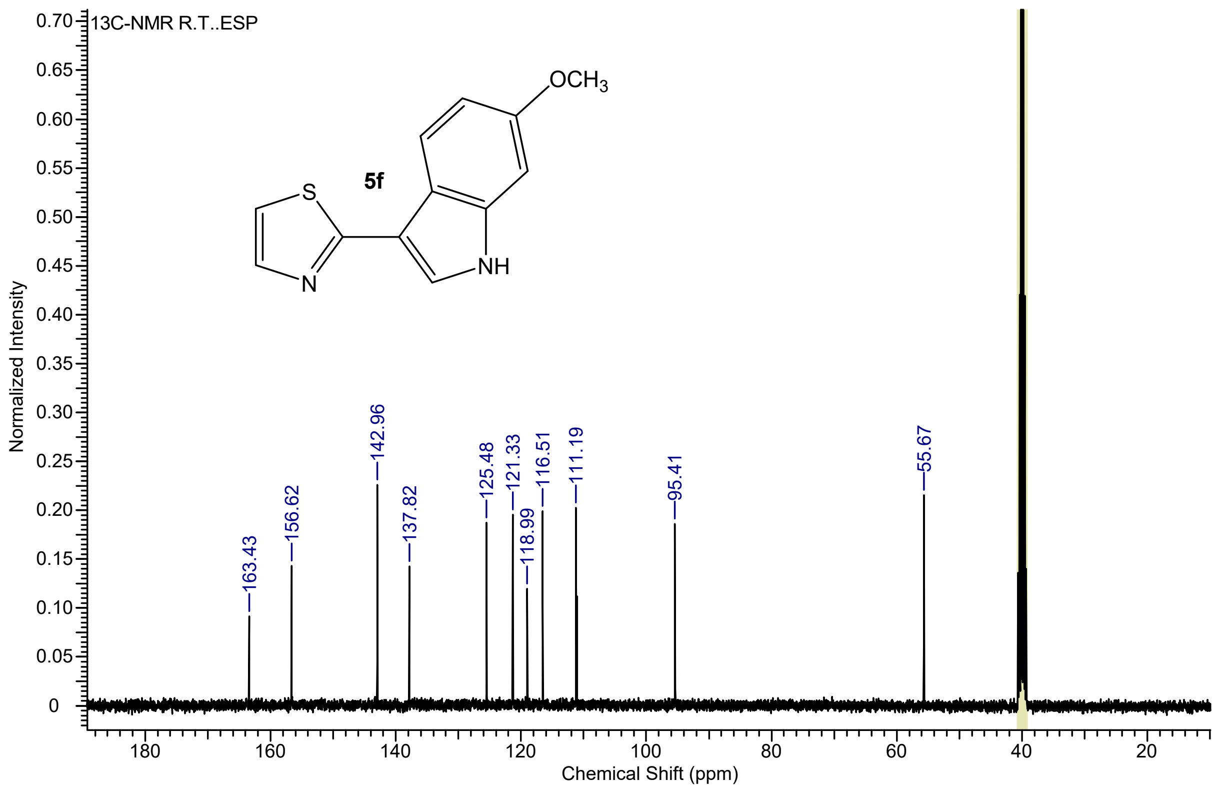

Figure S71 $^{13}C\{^{1}H\}$-NMR spectrum of **5f** - DMSO-d$_6$, r.t., 100 MHz

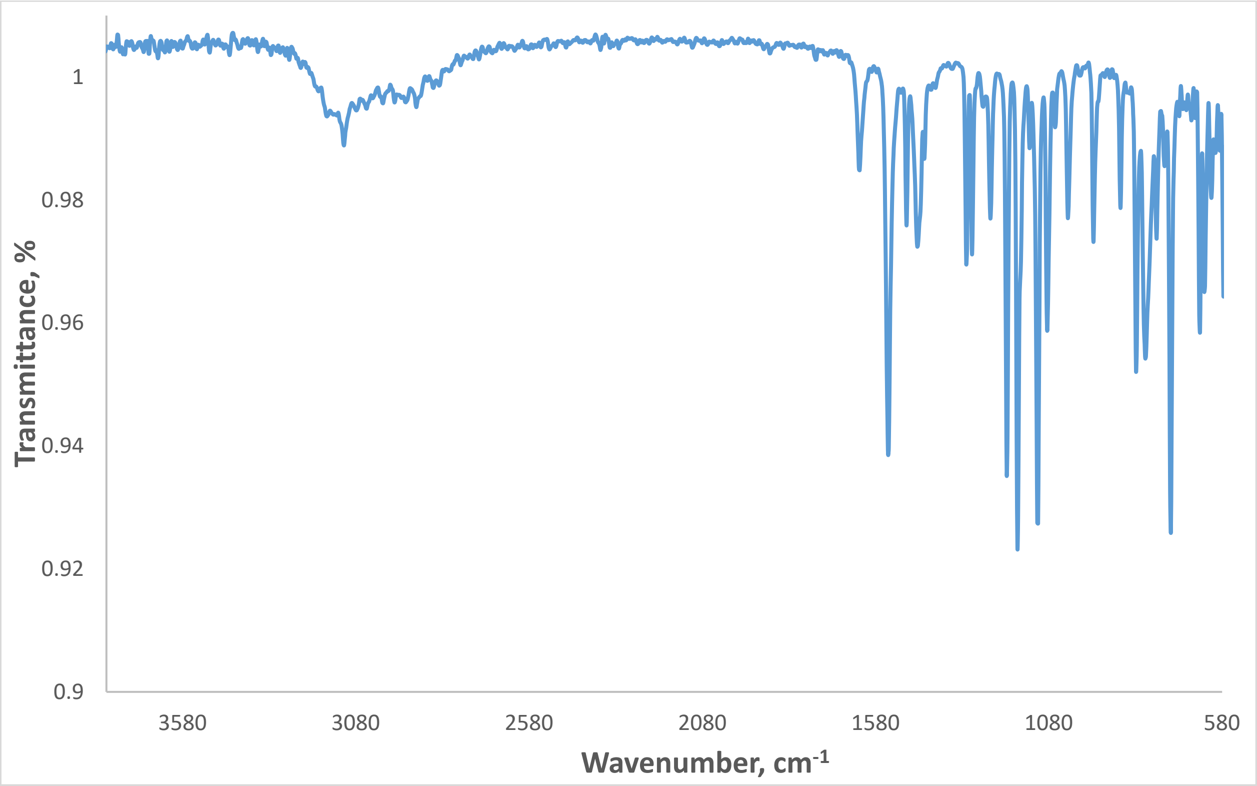

Figure S72 ATR-FTIR spectrum of compound **5f**, cm^{-1}

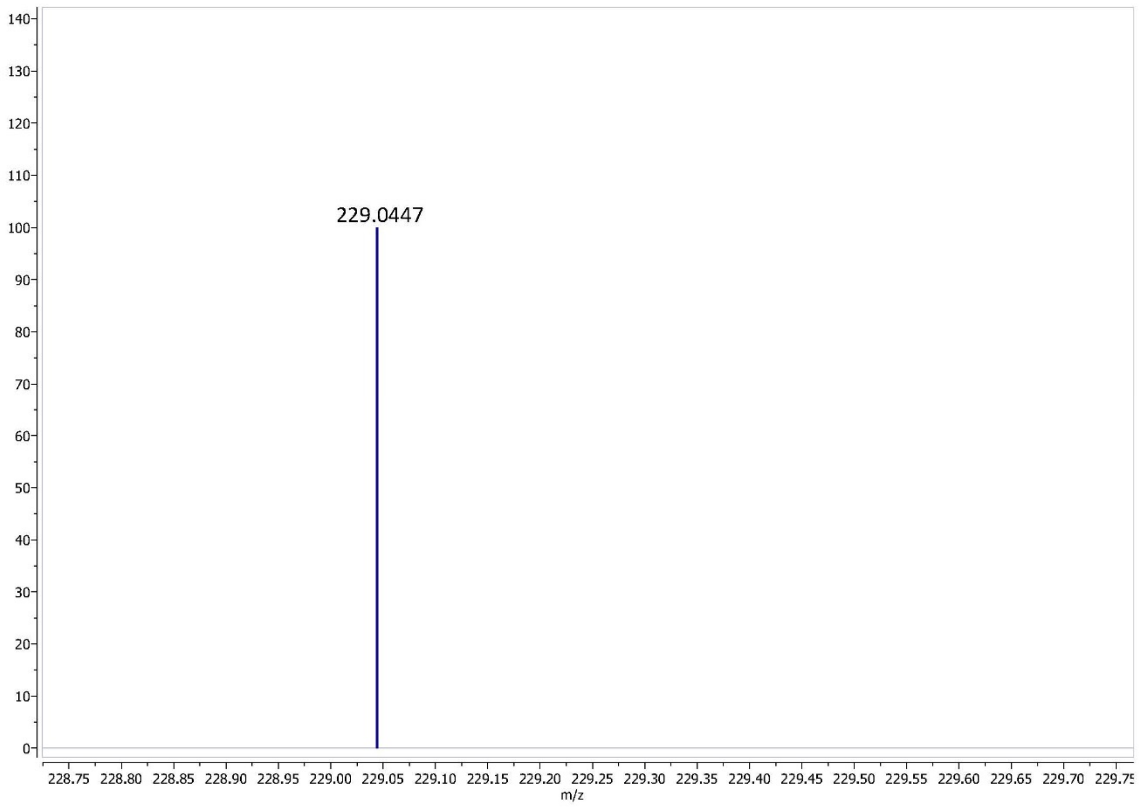

Figure S73 ESI-HRMS spectrum of compound **5f**, negative mode

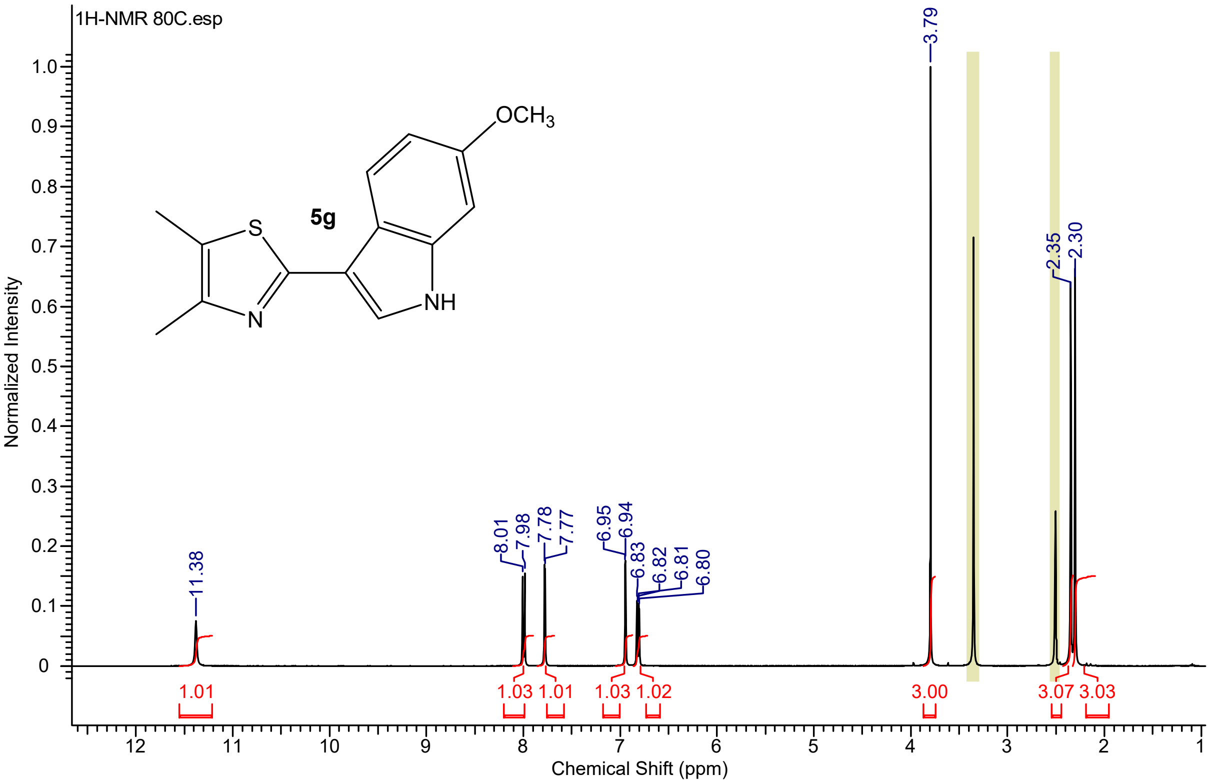

Figure S74 ¹H-NMR spectrum of **5g** - DMSO-d₆, r.t., 400 MHz

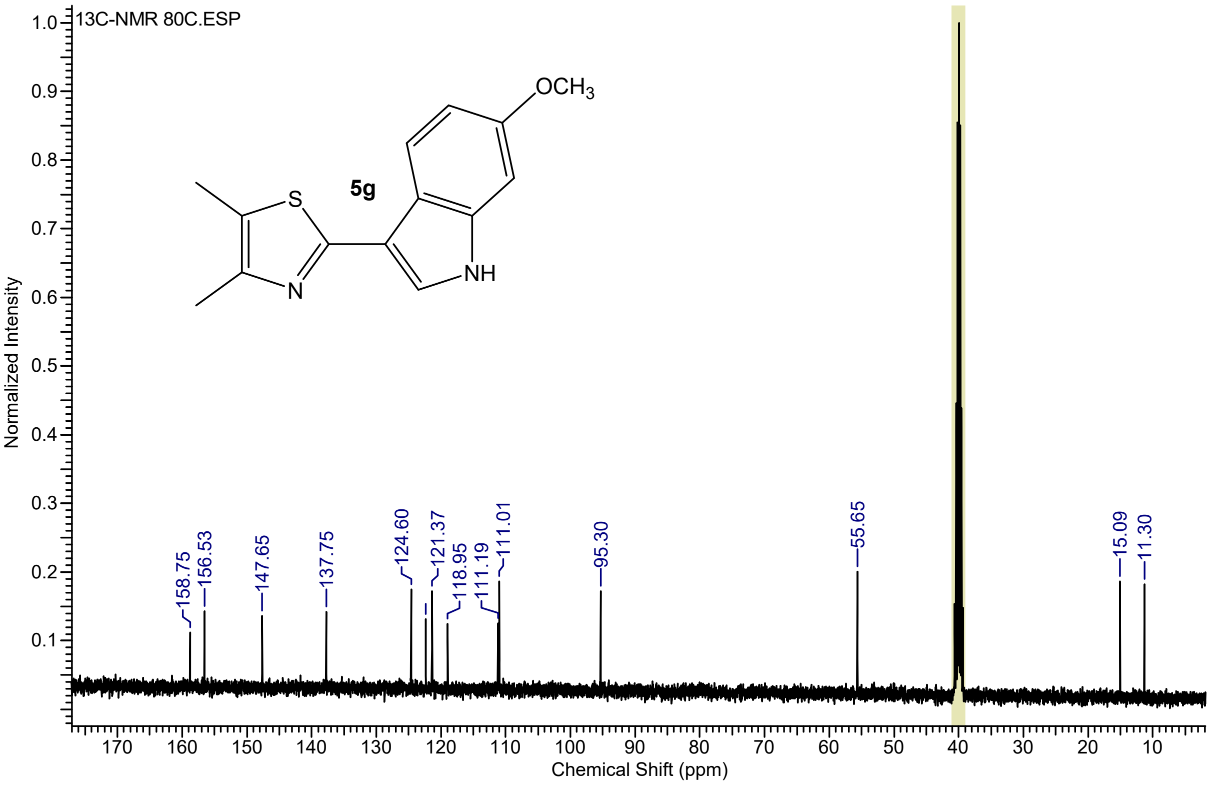

Figure S75 $^{13}C\{^{1}H\}$-NMR spectrum of **5g** - DMSO-d$_6$, r.t., 100 MHz

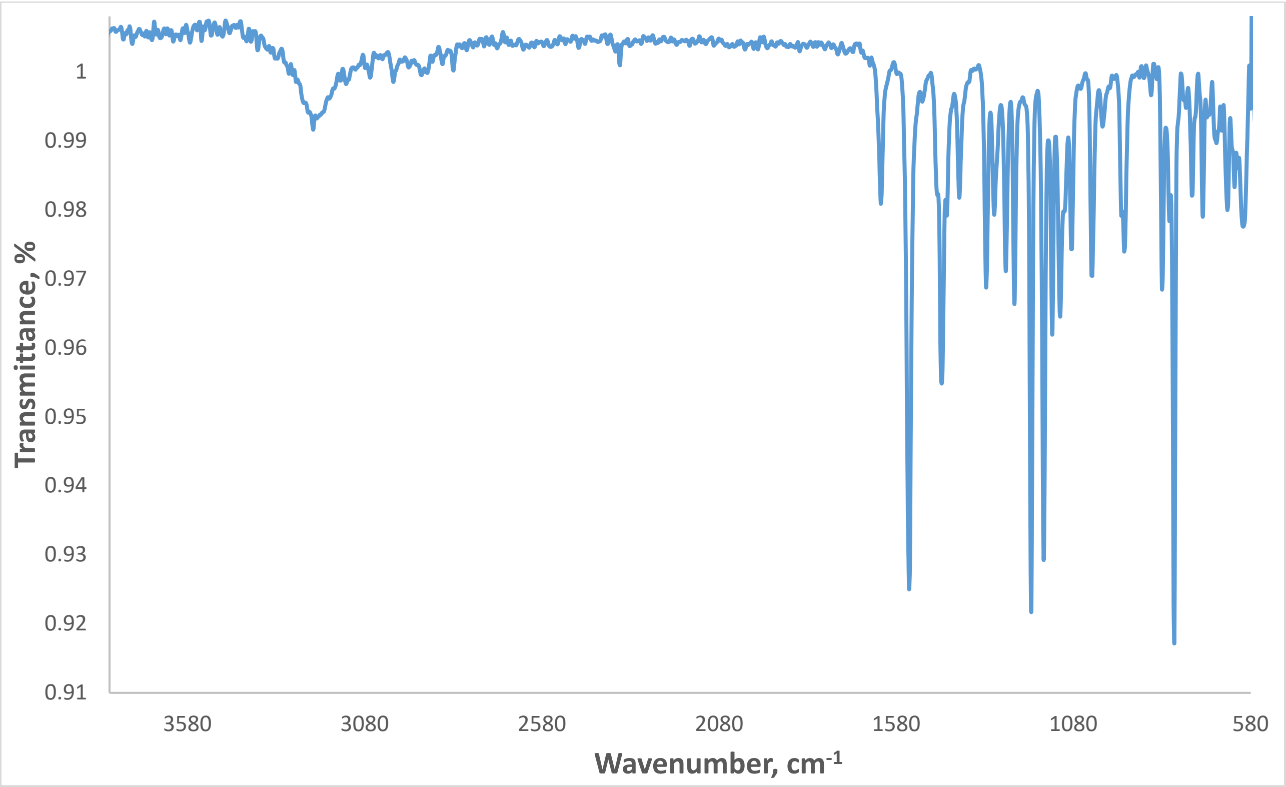

Figure S76 ATR-FTIR spectrum of compound **5g**, cm^{-1}

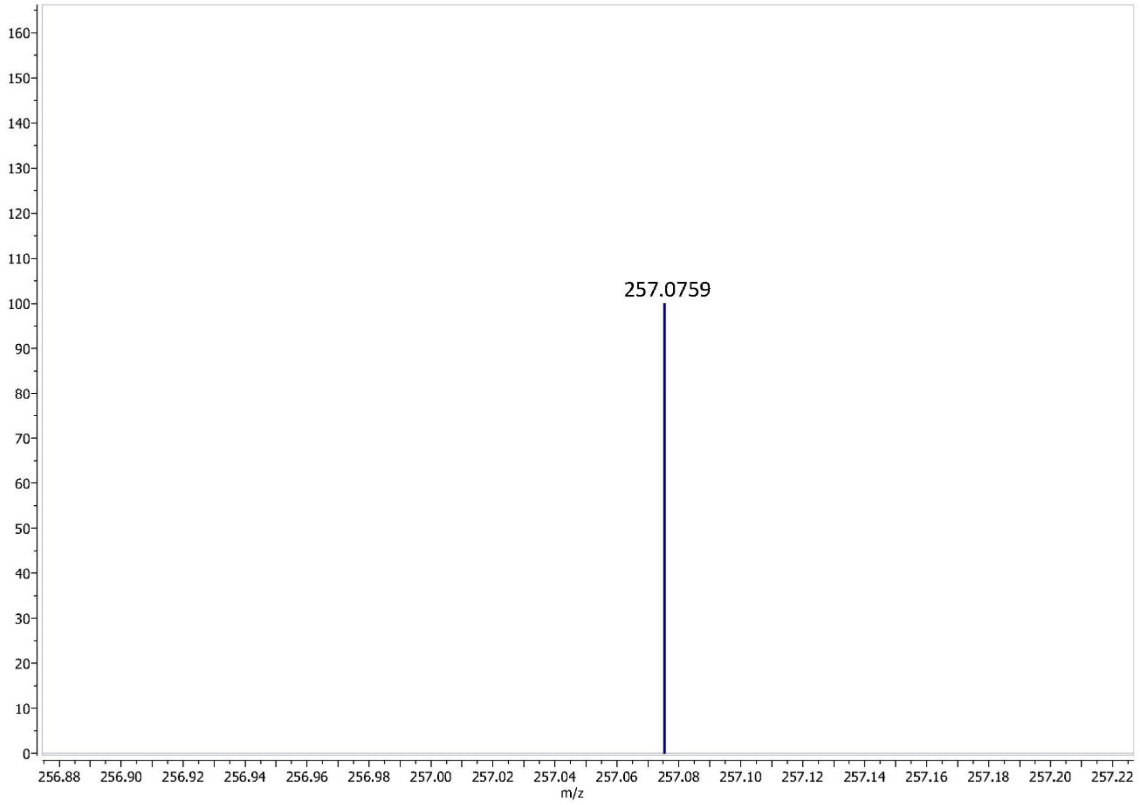

Figure S77 ESI-HRMS spectrum of compound **5g**, negative mode

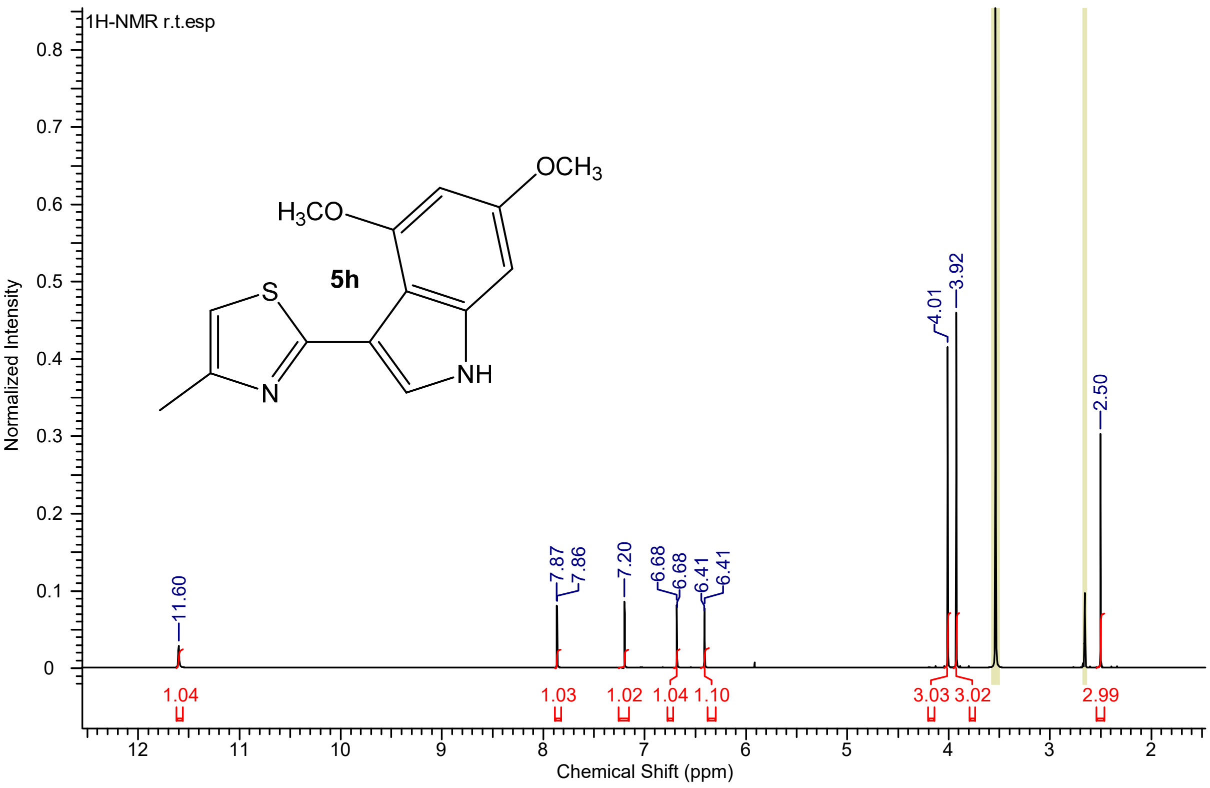

Figure S78 ^{1}H-NMR spectrum of **5h** - DMSO-d_6, r.t., 600 MHz

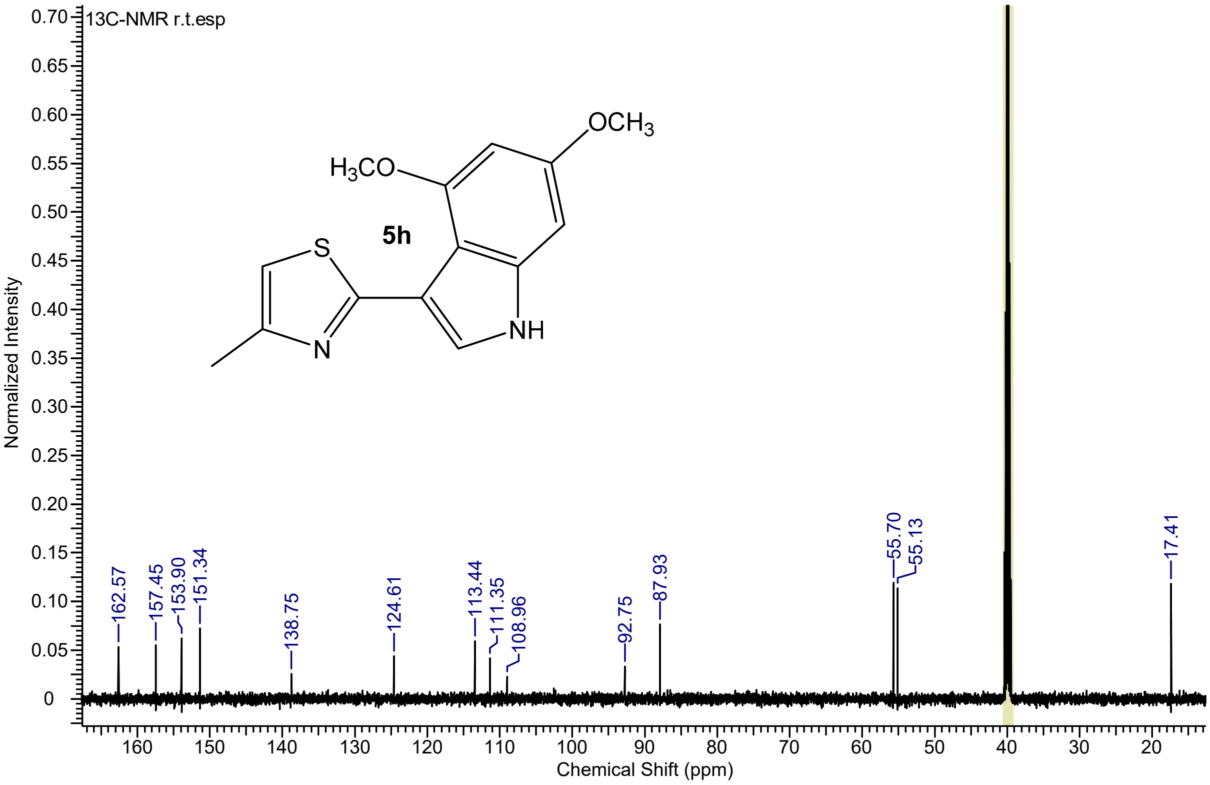

Figure S79 $^{13}C\{^{1}H\}$-NMR spectrum of **5h** - DMSO-d$_6$, r.t., 150 MHz

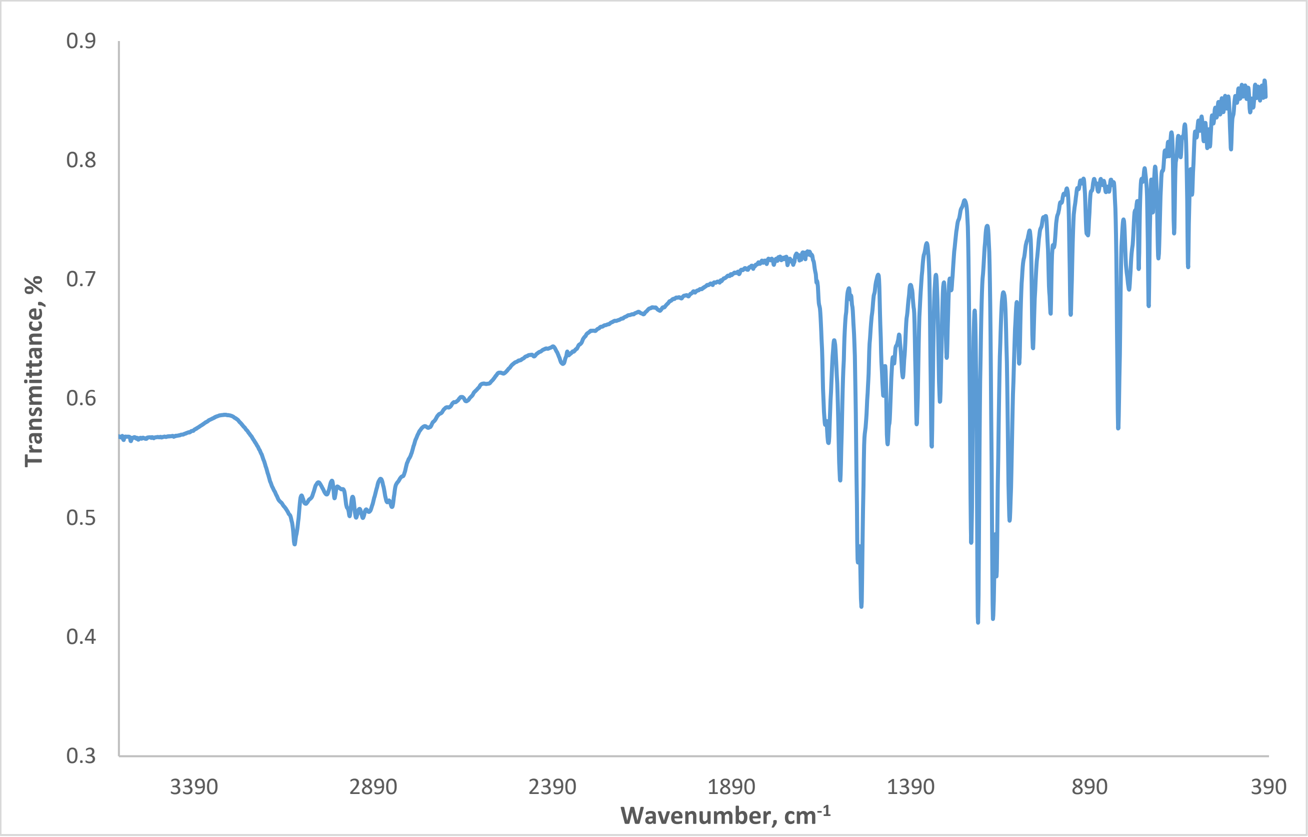

Figure S80 FTIR spectrum of compound **5h**, KBr tablet, cm^{-1}

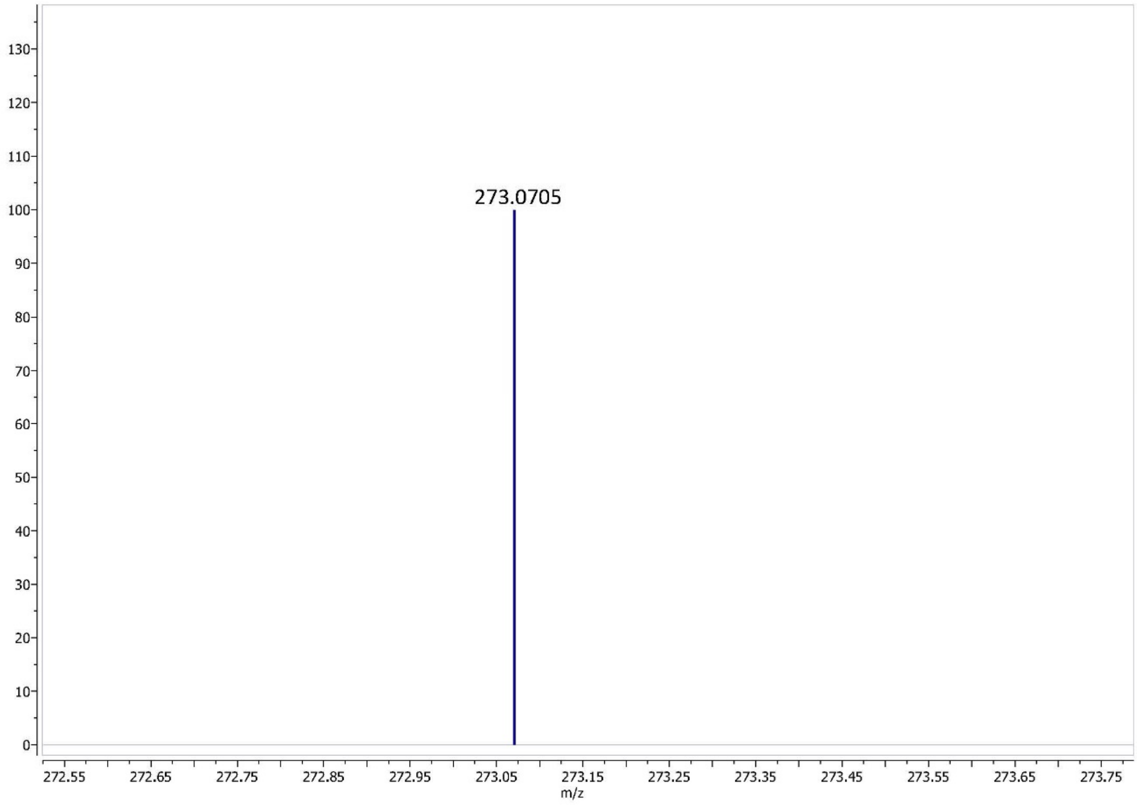

Figure S81 ESI-HRMS spectrum of compound **5h**, negative mode

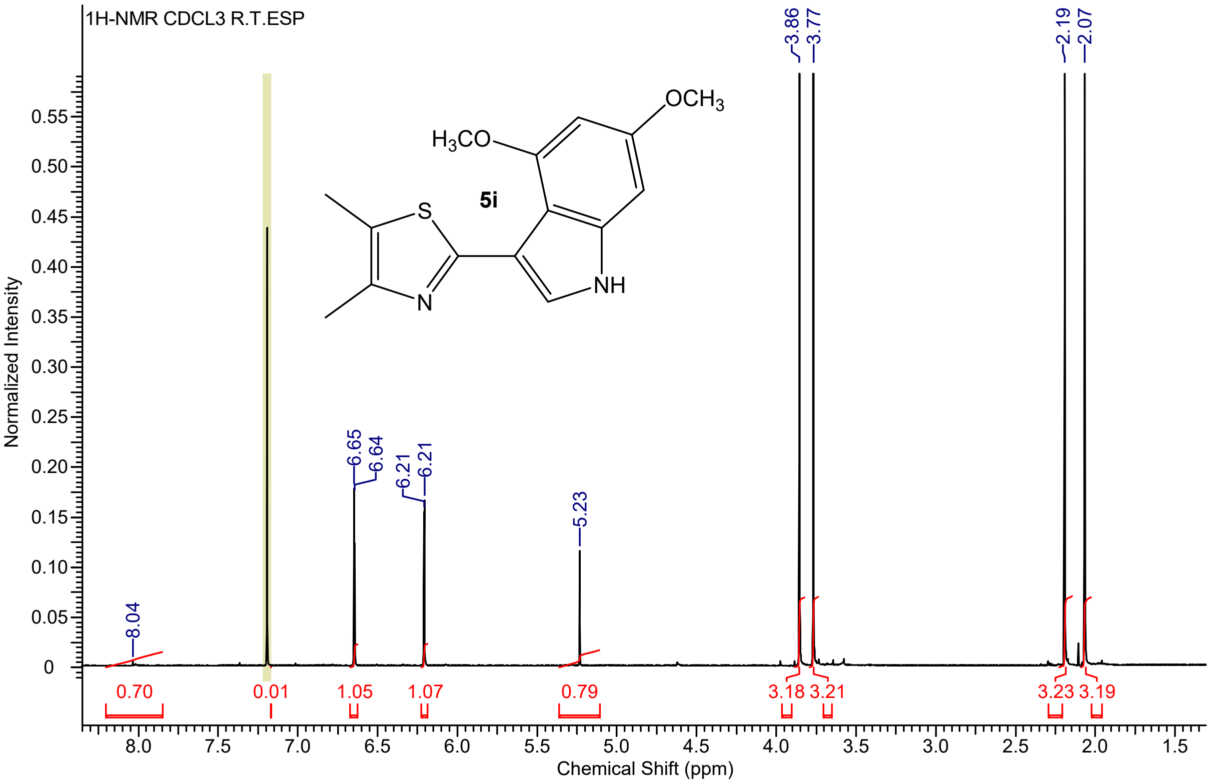

Figure S82 ^{1}H-NMR spectrum of **5i** - $CDCl_3$, r.t., 600 MHz

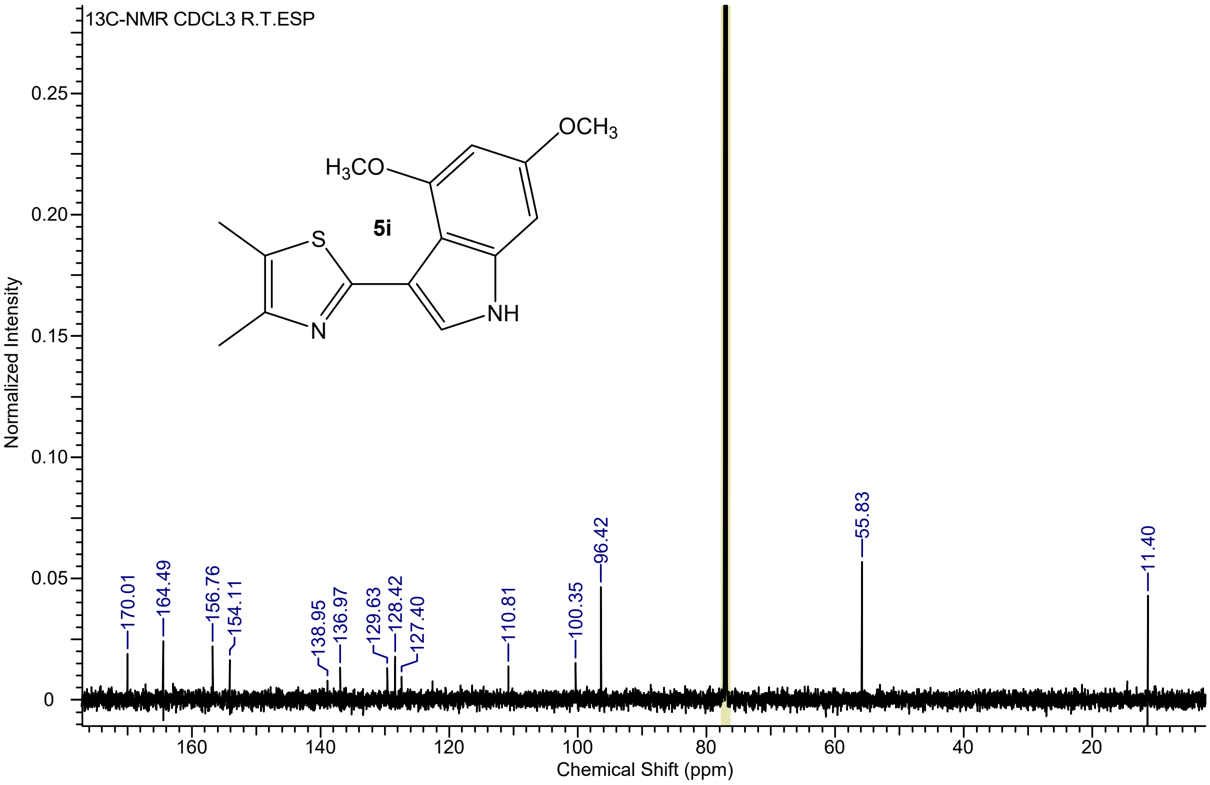

Figure S83 $^{13}C\{^{1}H\}$-NMR spectrum of **5i** - $CDCl_3$, r.t., 150 MHz

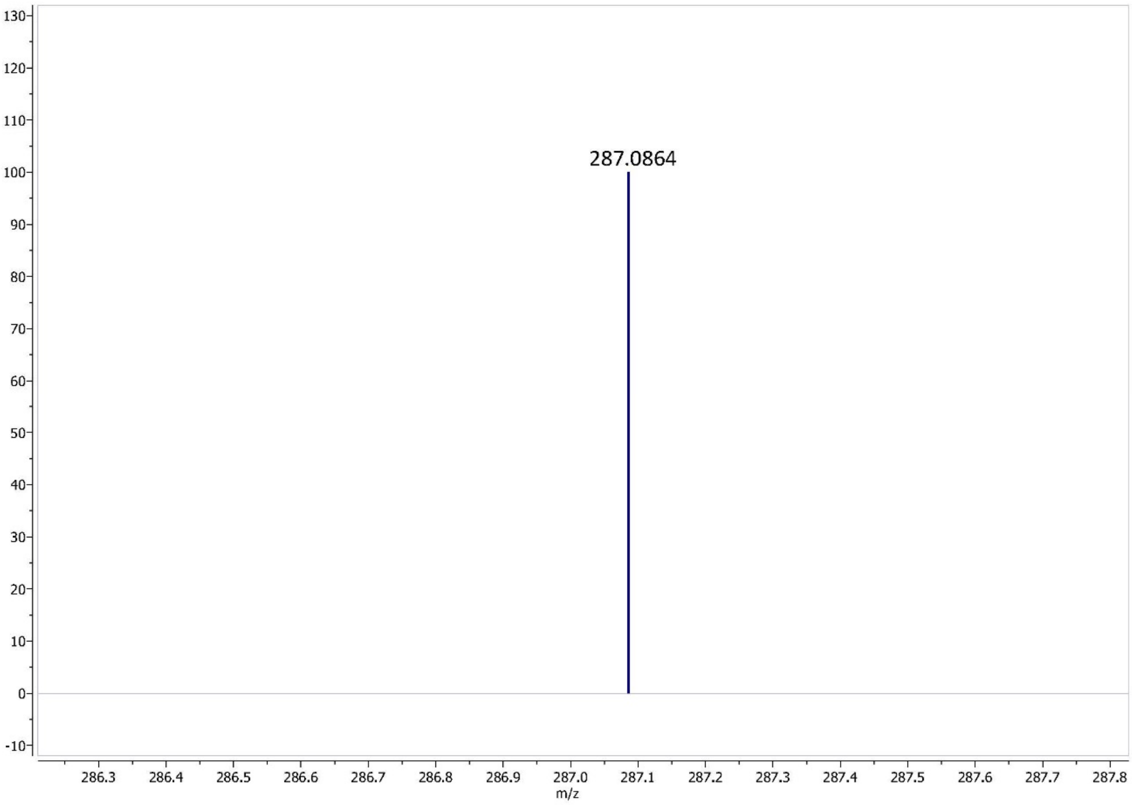

Figure S84 ESI-HRMS spectrum of compound **5i**, negative mode

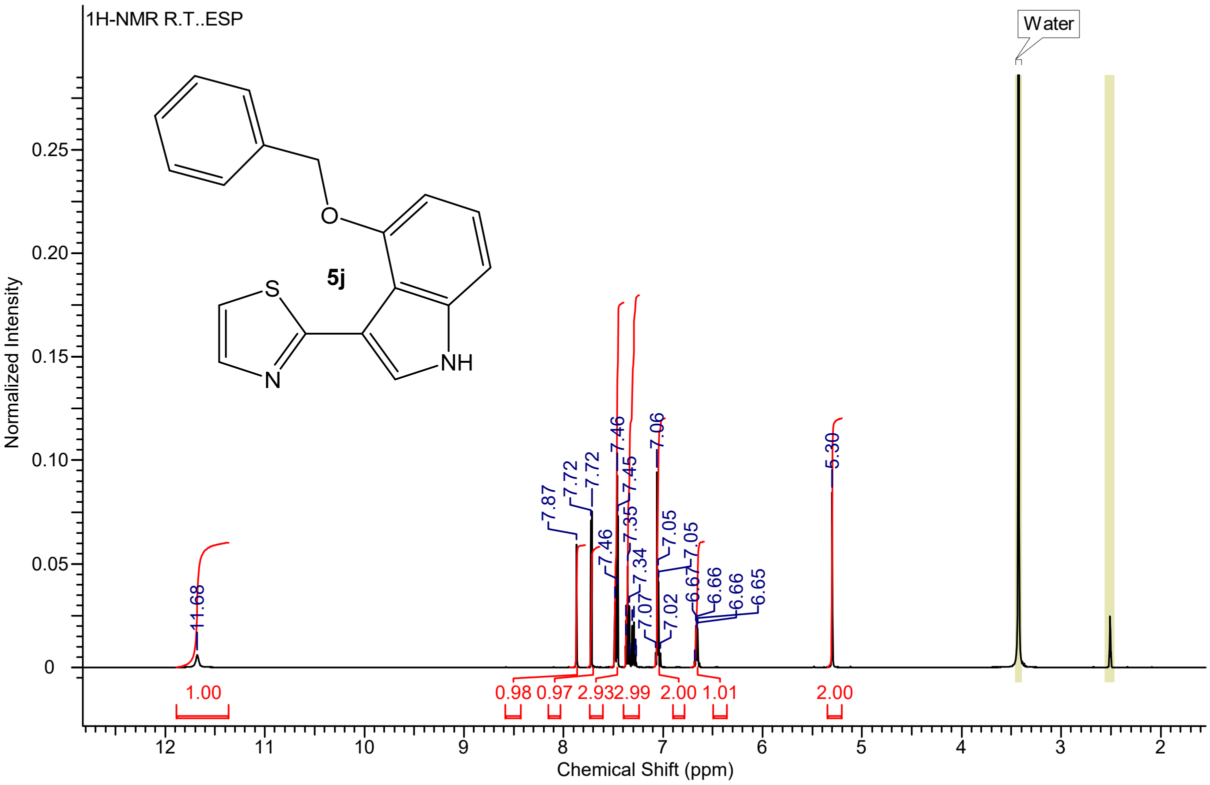

Figure S85 ^{1}H-NMR spectrum of **5j** - DMSO-d6, r.t., 400 MHz

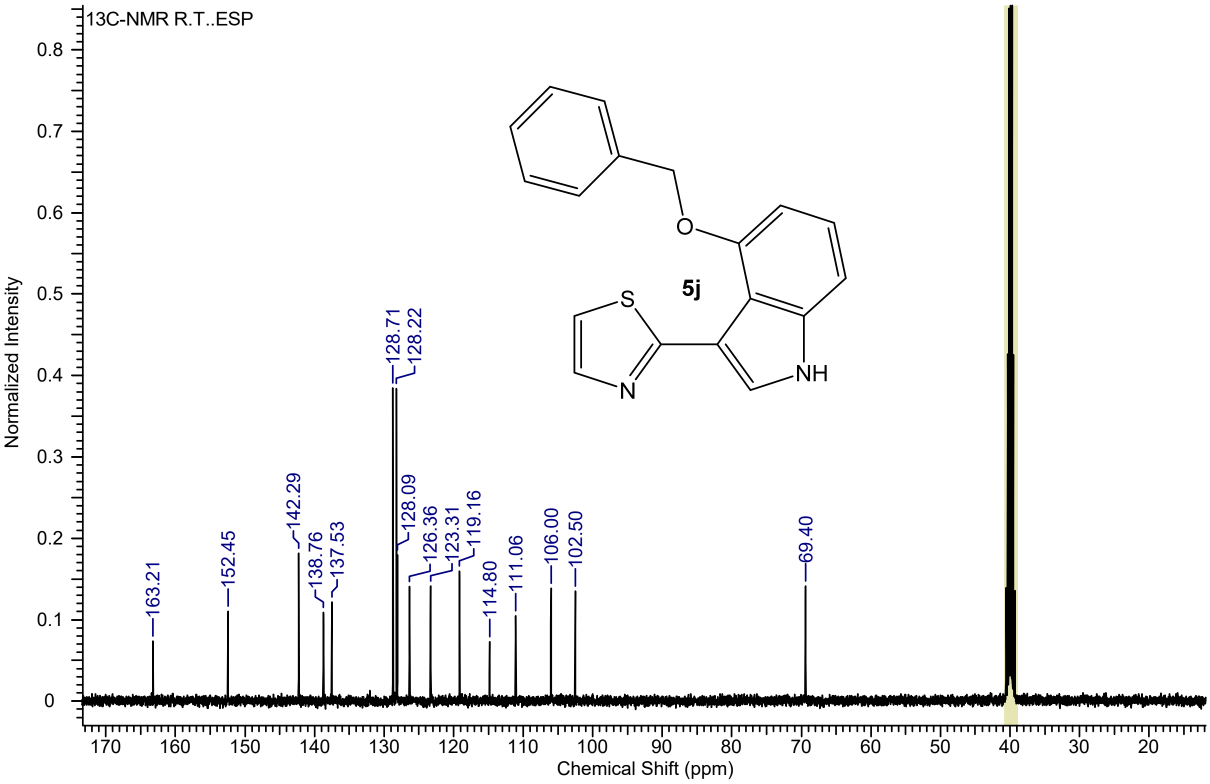

Figure S86 ^{13}C{^{1}H}-NMR spectrum of **5j** - DMSO-d6, r.t., 100 MHz

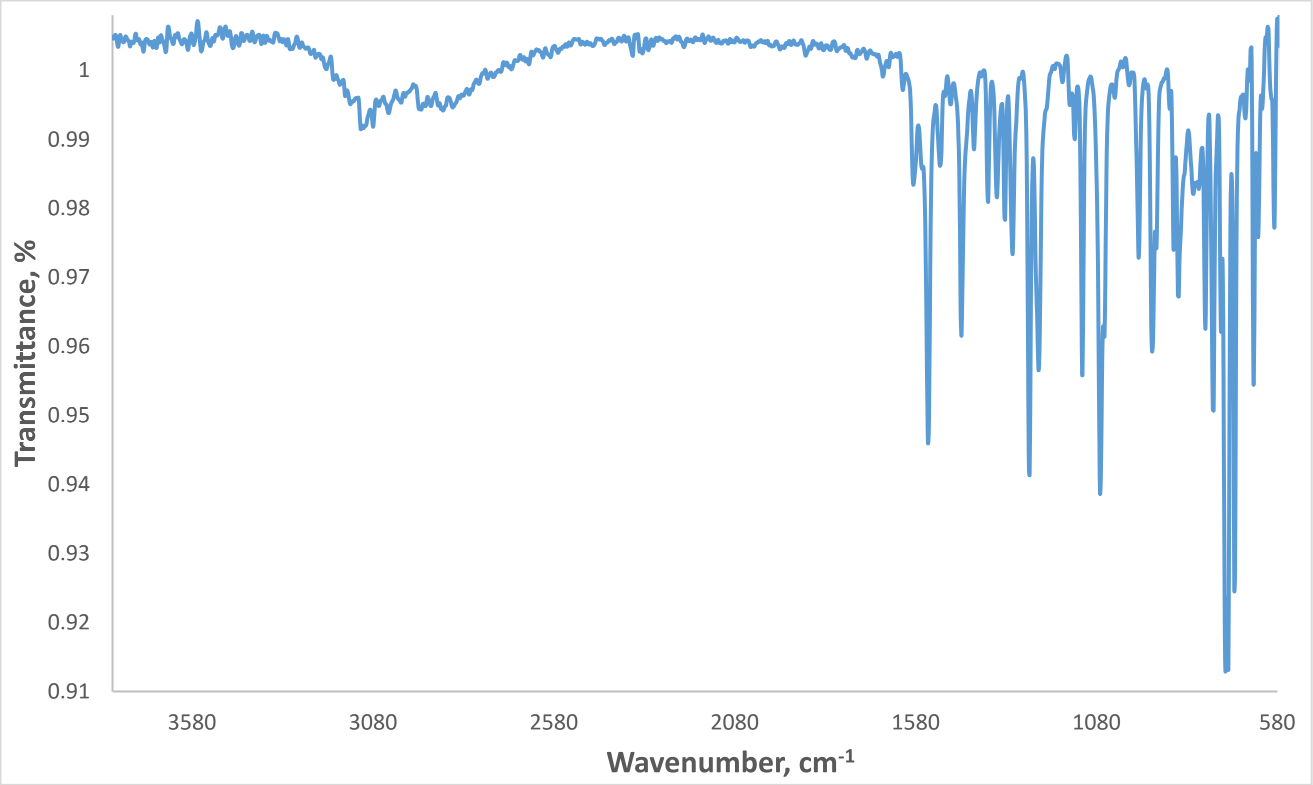

Figure S87 ATR-FTIR spectrum of compound **5j**, cm^{-1}

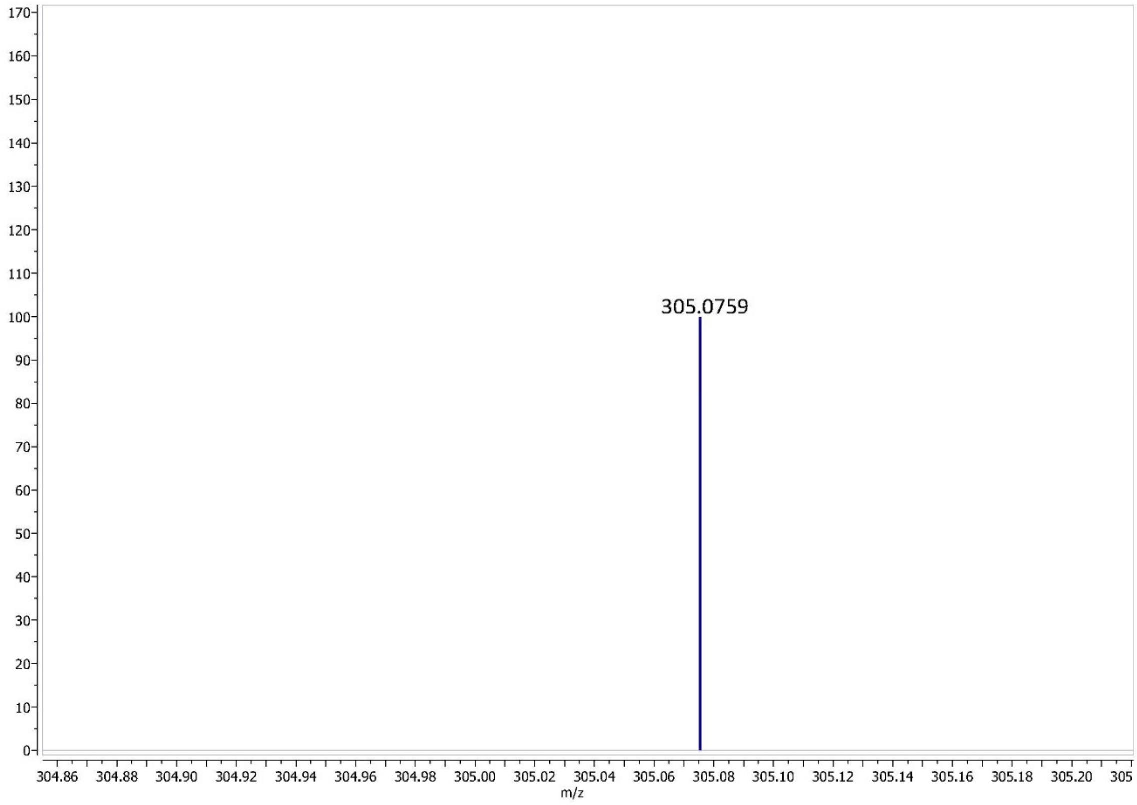

Figure S88 ESI-HRMS spectrum of compound **5j**, negative mode

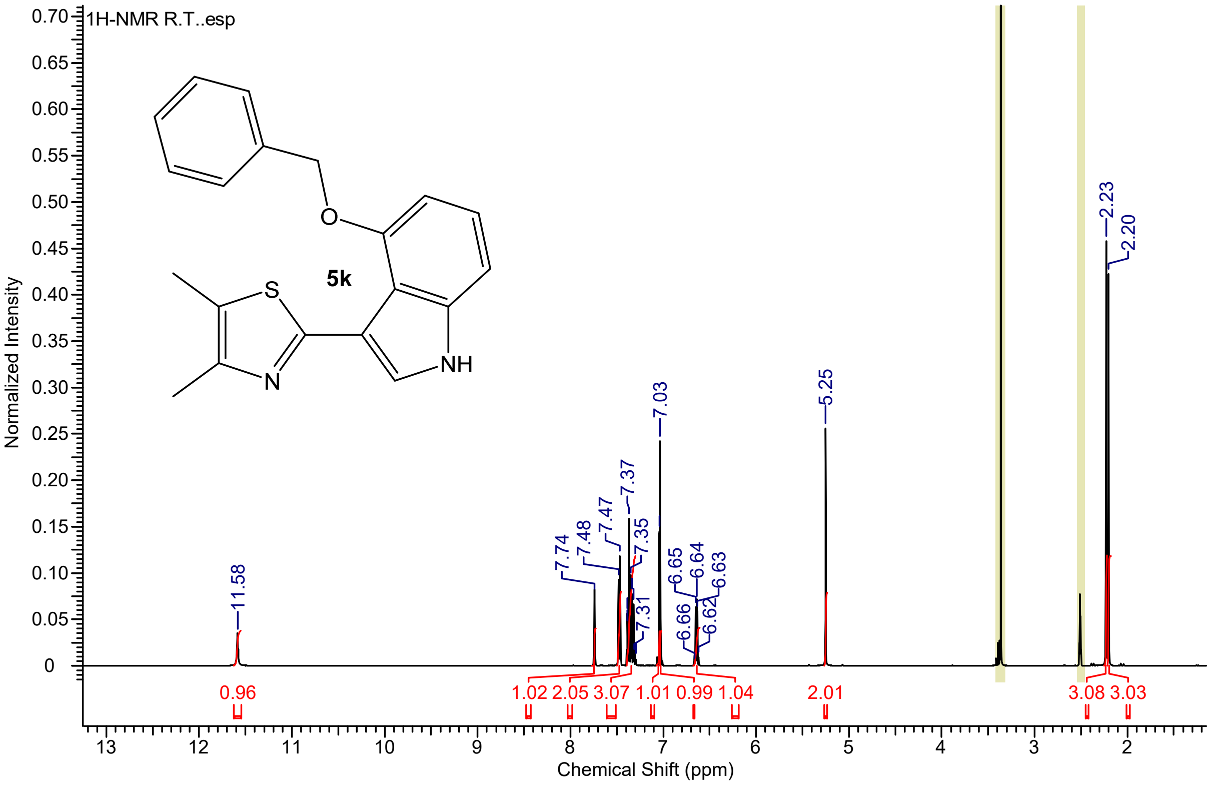

Figure S89 ^{1}H-NMR spectrum of **5k** - DMSO-d$_6$, r.t., 400 MHz

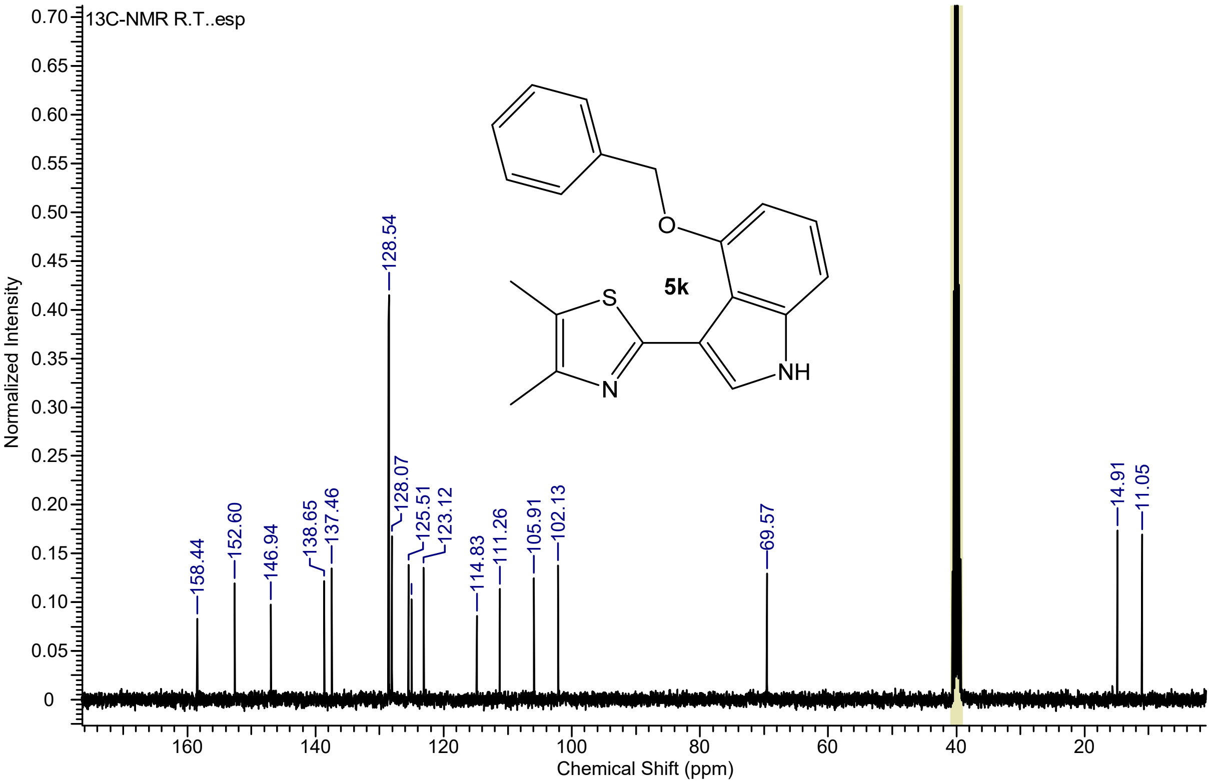

Figure S90 ^{13}C{^{1}H}-NMR spectrum of **5k** - DMSO-d$_6$, r.t., 100 MHz

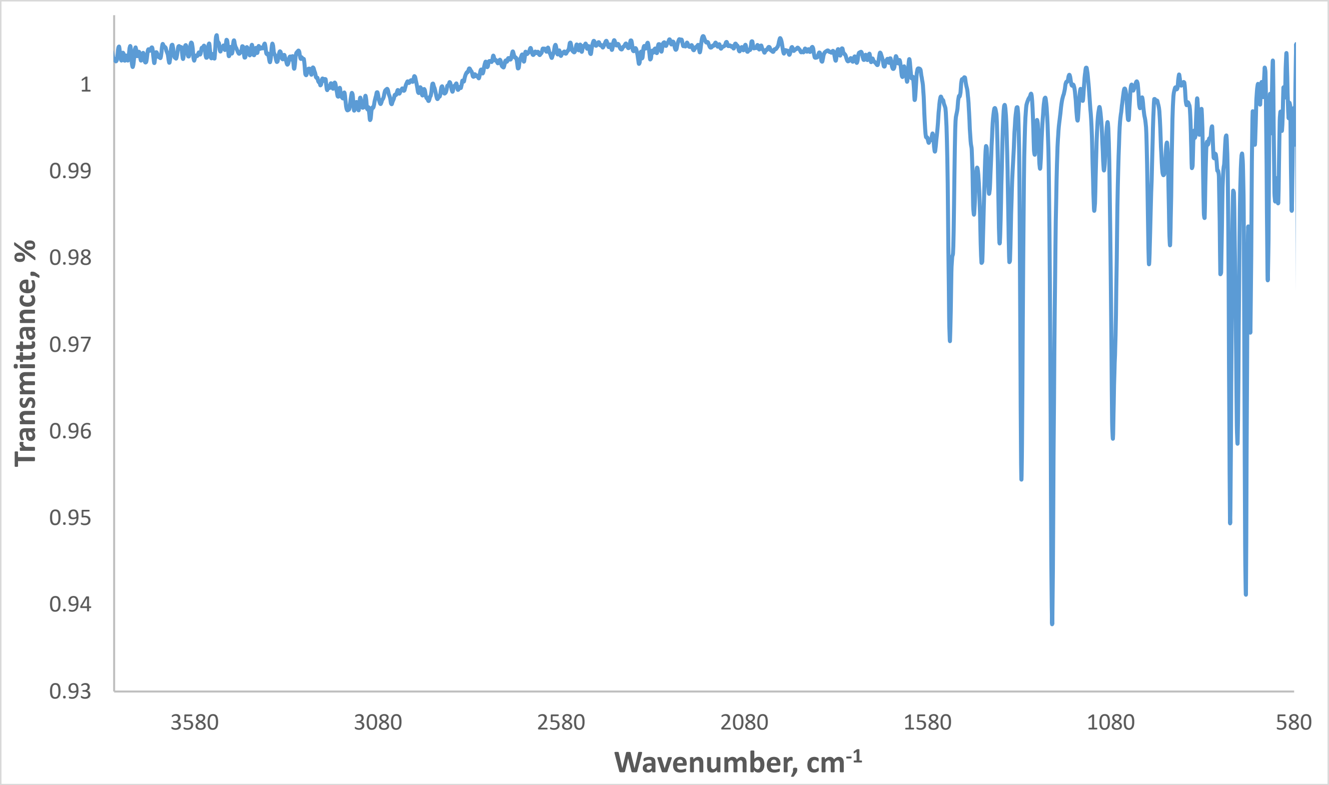

Figure S91 ATR-FTIR spectrum of compound **5k**, cm^{-1}

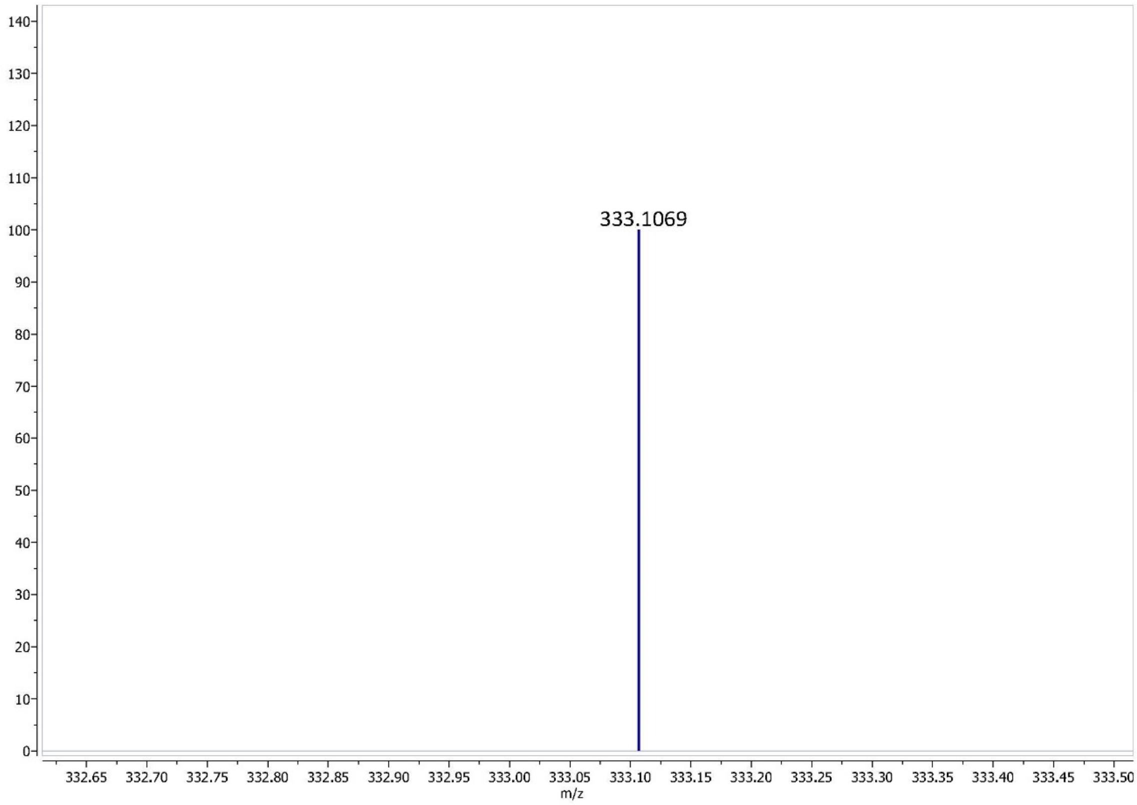

Figure S92 ESI-HRMS spectrum of compound **5k**, negative mode

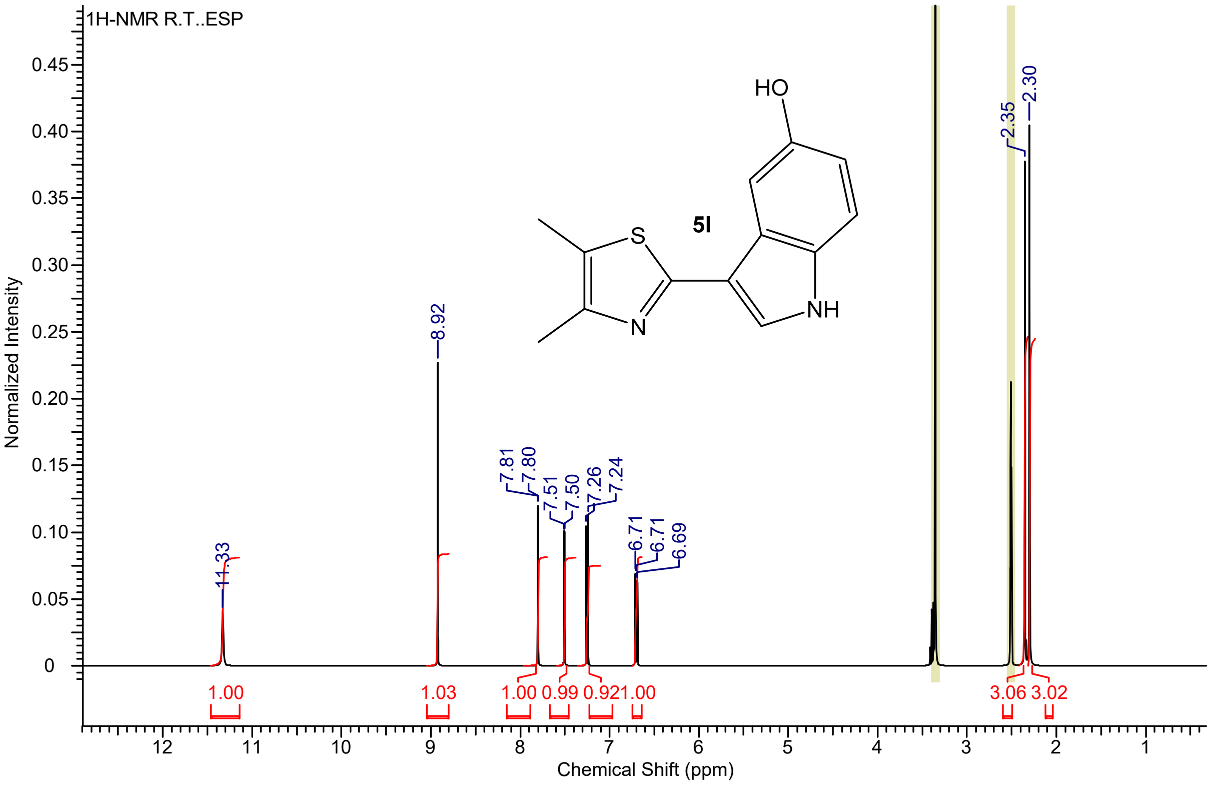

Figure S93 ^{1}H-NMR spectrum of **5l** - DMSO-d6, r.t., 400 MHz

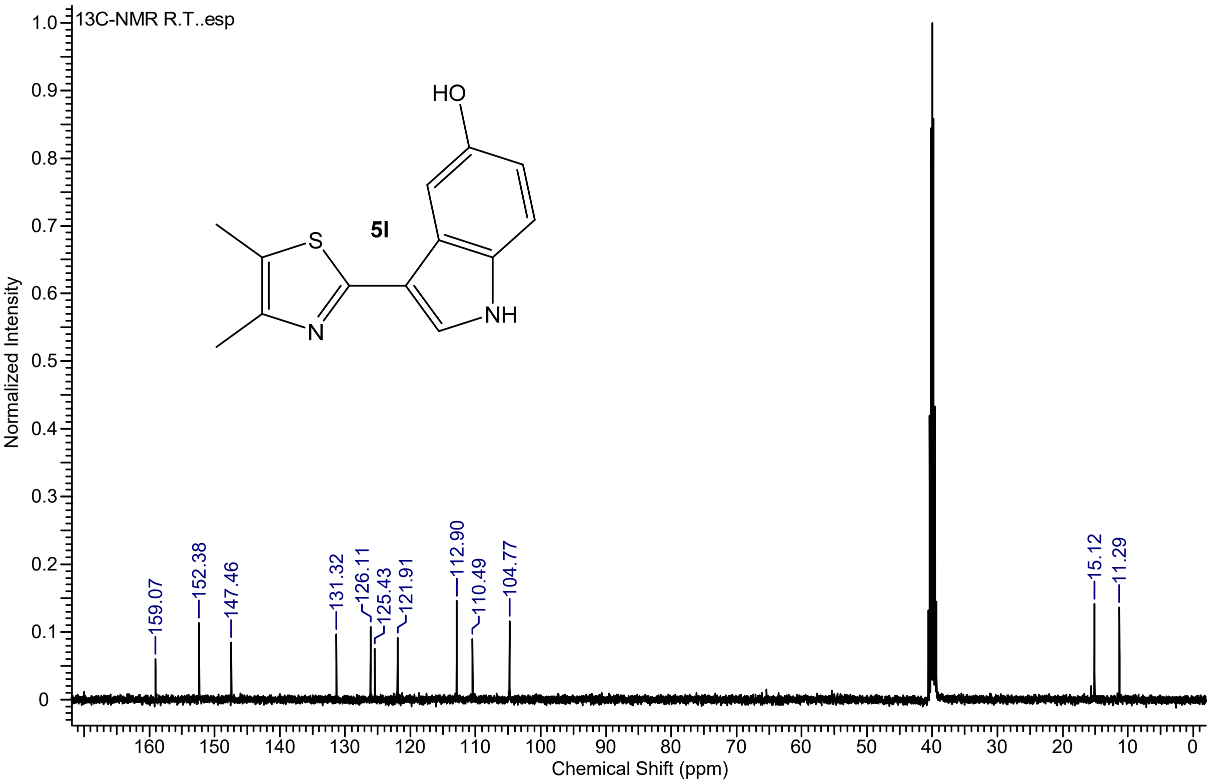

Figure S94 ^{13}C{^{1}H}-NMR spectrum of **5l** - DMSO-d6, r.t., 100 MHz

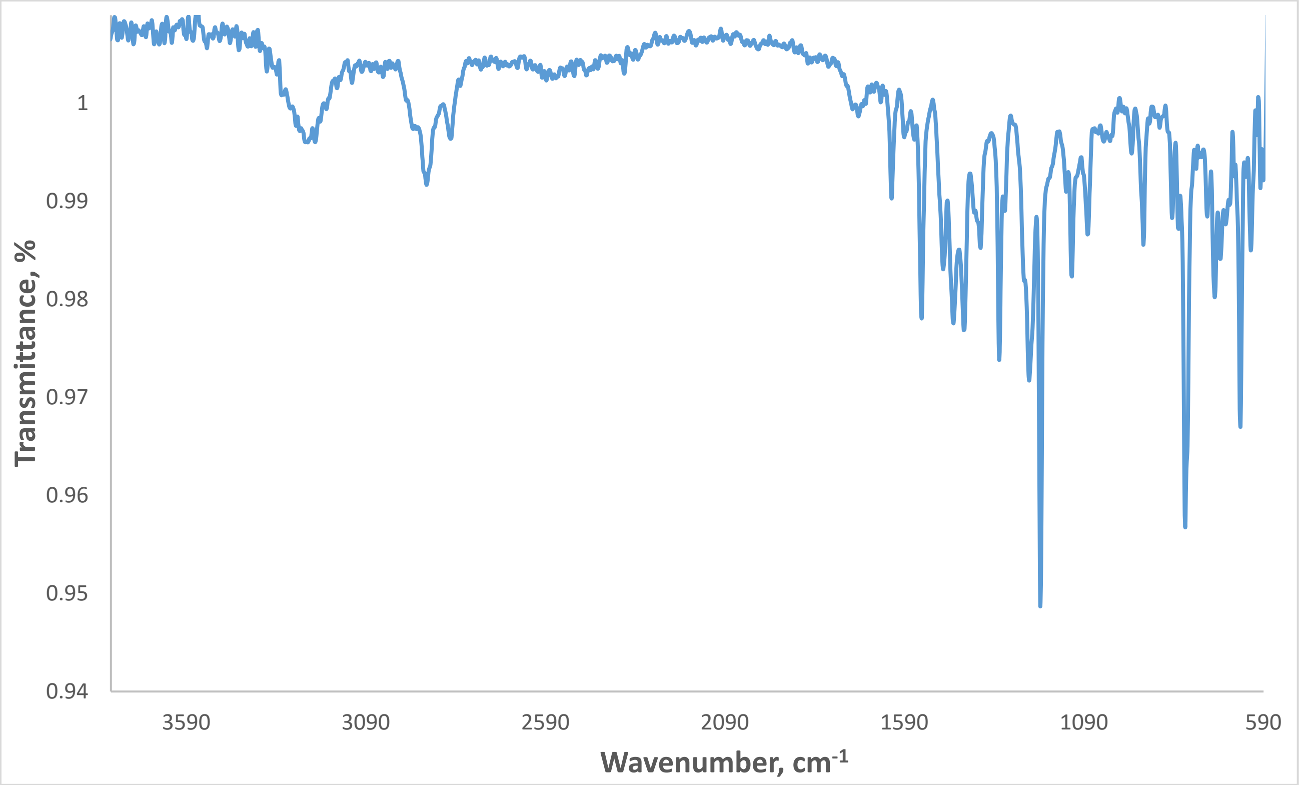

Figure S95 ATR-FTIR spectrum of compound **5l**, cm^{-1}

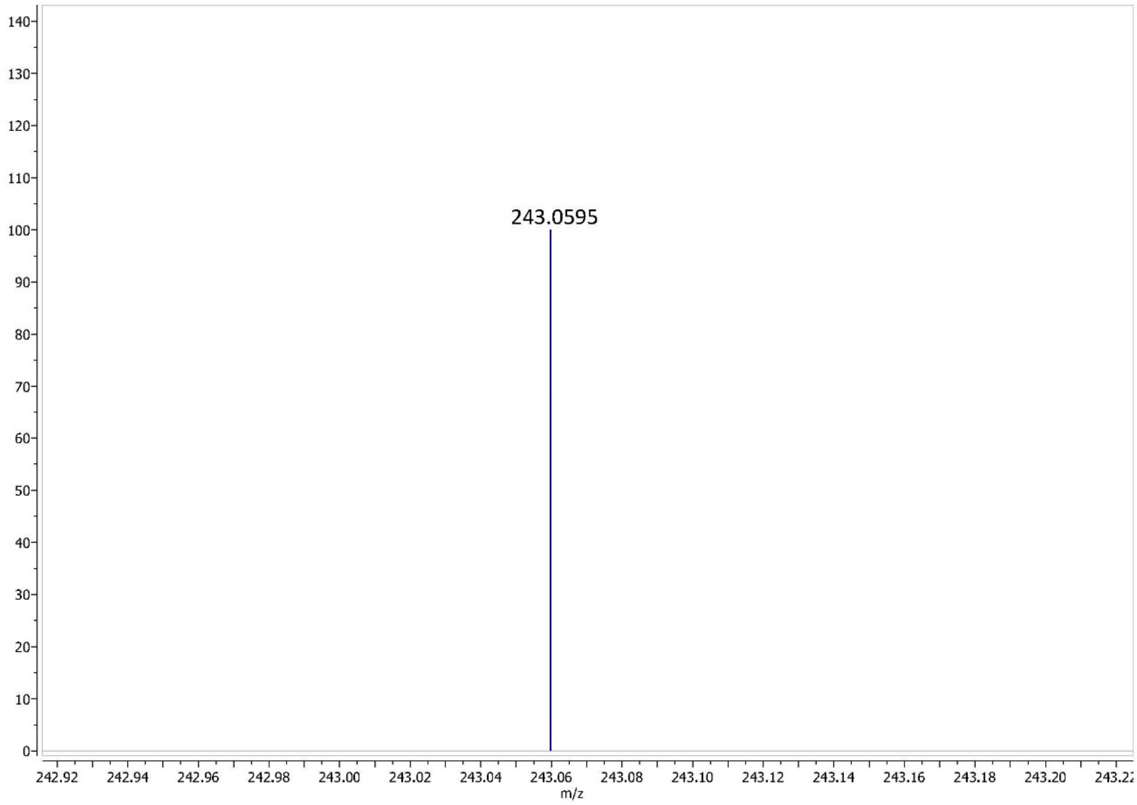

Figure S96 ESI-HRMS spectrum of compound **5l**, negative mode

Table S1 Structures and abbreviations of not synthesized compounds

Compound	Structure, Julia language
**p1**	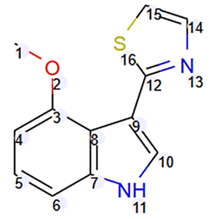
**p8**	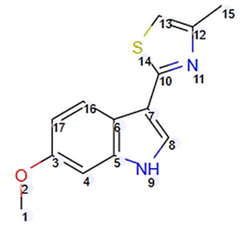
**p10**	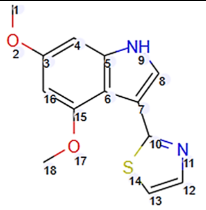
**p14**	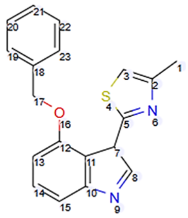
**p16**	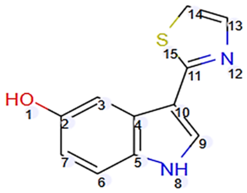
**p17**	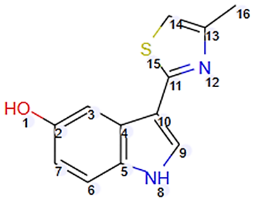

Table S2. Lipinski Rule of Five (Julia language – Molecular Graph package).

Compound	HBA	HBD	HBA + HBD	NRotB
**p1**	3	1	4	2
**p2**	3	1	4	2
**p3**	3	1	4	2
**p4**	3	1	4	2
**p5**	3	1	4	2
**p6**	3	1	4	2
**p7**	3	1	4	2
**p8**	3	1	4	2
**p9**	3	1	4	2
**p10**	4	1	5	3
**p11**	4	1	5	3
**p12**	4	1	5	3
**p13**	3	1	4	4
**p14**	3	0	3	4
**p15**	3	0	3	4
**p16**	3	2	5	1
**p17**	3	2	5	1
**p18**	3	2	5	1

Table S3. Atomic hybridization assessment, implemented with Julia language – Molecular Graph package.

Compound	Number of π-electrons	*sp*	sp^2	sp^3	Number of unpaired electron
**p1**	12	0	12	4	16
**p2**	12	0	12	5	17
**p3**	12	0	12	6	18
**p4**	12	0	12	4	16
**p5**	12	0	12	5	17
**p6**	12	0	12	6	18
**p7**	12	0	12	4	16
**p8**	12	0	12	5	17
**p9**	12	0	12	6	18
**p10**	12	0	12	6	18
**p11**	12	0	12	6	19
**p12**	12	0	12	8	20
**p13**	18	0	18	4	22
**p14**	18	0	18	5	23
**p15**	18	0	18	6	24
**p16**	12	0	12	3	15
**p17**	12	0	12	4	16
**p18**	12	0	12	5	17

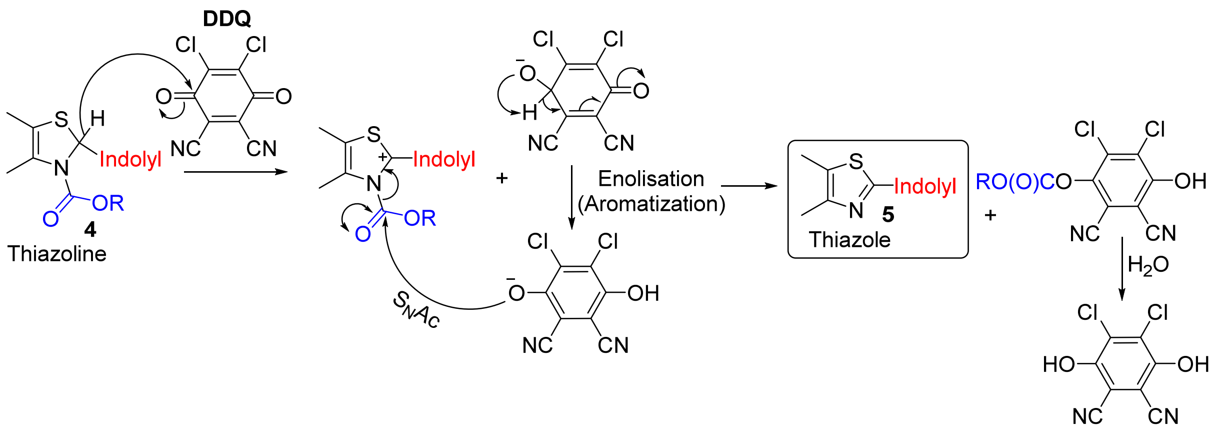

Scheme S1 Proposed mechanism of the oxidative rearomatization with DDQ (2,3-dichloro-5,6-dicyano-1,4-benzoquinone) for the synthesis of oxy-camalexins
